# Integrative analysis of epigenetics data identifies gene-specific regulatory elements

**DOI:** 10.1093/nar/gkab798

**Published:** 2021-09-11

**Authors:** Florian Schmidt, Alexander Marx, Nina Baumgarten, Marie Hebel, Martin Wegner, Manuel Kaulich, Matthias S Leisegang, Ralf P Brandes, Jonathan Göke, Jilles Vreeken, Marcel H Schulz

**Affiliations:** Cluster of Excellence for Multimodal Computing and Interaction, Saarland University, Saarland Informatics Campus, 66123 Saarbrücken, Germany; Max Planck Institute for Informatics, Saarland Informatics Campus, 66123 Saarbrücken, Germany; Graduate School of Computer Science, Saarland Informatics Campus, 66123 Saarbrücken, Germany; Laboratory of Systems Biology and Data Analytics, Genome Institute of Singapore, 60 Biopolis Street, 138672 Singapore, Singapore; Cluster of Excellence for Multimodal Computing and Interaction, Saarland University, Saarland Informatics Campus, 66123 Saarbrücken, Germany; Max Planck Institute for Informatics, Saarland Informatics Campus, 66123 Saarbrücken, Germany; Graduate School of Computer Science, Saarland Informatics Campus, 66123 Saarbrücken, Germany; International Max Planck Research School for Computer Science, Saarland Informatics Campus, 66123 Saarbrücken, Germany; Institute for Cardiovascular Regeneration, Goethe University, 60590 Frankfurt am Main, Germany; German Center for Cardiovascular Research (DZHK), Partner site RheinMain, 60590 Frankfurt am Main, Germany; Institute of Biochemistry II, Goethe University Frankfurt - Medical Faculty, University Hospital, 60590 Frankfurt am Main, Germany; Institute of Biochemistry II, Goethe University Frankfurt - Medical Faculty, University Hospital, 60590 Frankfurt am Main, Germany; Institute of Biochemistry II, Goethe University Frankfurt - Medical Faculty, University Hospital, 60590 Frankfurt am Main, Germany; Frankfurt Cancer Institute, Goethe University, 60590 Frankfurt am Main, Germany; German Center for Cardiovascular Research (DZHK), Partner site RheinMain, 60590 Frankfurt am Main, Germany; Institute for Cardiovascular Physiology, Goethe University, 60590 Frankfurt am Main, Germany; German Center for Cardiovascular Research (DZHK), Partner site RheinMain, 60590 Frankfurt am Main, Germany; Institute for Cardiovascular Physiology, Goethe University, 60590 Frankfurt am Main, Germany; Laboratory of Computational Transcriptomics, Genome Institute of Singapore, 60 Biopolis Street, 138672 Singapore, Singapore; CISPA Helmholtz Center for Information Security, Saarland Informatics Campus, 66123 Saarbrücken, Germany; Cluster of Excellence for Multimodal Computing and Interaction, Saarland University, Saarland Informatics Campus, 66123 Saarbrücken, Germany; Max Planck Institute for Informatics, Saarland Informatics Campus, 66123 Saarbrücken, Germany; Cluster of Excellence for Multimodal Computing and Interaction, Saarland University, Saarland Informatics Campus, 66123 Saarbrücken, Germany; Max Planck Institute for Informatics, Saarland Informatics Campus, 66123 Saarbrücken, Germany; Institute for Cardiovascular Regeneration, Goethe University, 60590 Frankfurt am Main, Germany; German Center for Cardiovascular Research (DZHK), Partner site RheinMain, 60590 Frankfurt am Main, Germany

## Abstract

Understanding how epigenetic variation in non-coding regions is involved in distal gene-expression regulation is an important problem. Regulatory regions can be associated to genes using large-scale datasets of epigenetic and expression data. However, for regions of complex epigenomic signals and enhancers that regulate many genes, it is difficult to understand these associations. We present StitchIt, an approach to dissect epigenetic variation in a gene-specific manner for the detection of regulatory elements (REMs) without relying on peak calls in individual samples. StitchIt segments epigenetic signal tracks over many samples to generate the location and the target genes of a REM simultaneously. We show that this approach leads to a more accurate and refined REM detection compared to standard methods even on heterogeneous datasets, which are challenging to model. Also, StitchIt REMs are highly enriched in experimentally determined chromatin interactions and expression quantitative trait loci. We validated several newly predicted REMs using CRISPR-Cas9 experiments, thereby demonstrating the reliability of StitchIt. StitchIt is able to dissect regulation in superenhancers and predicts thousands of putative REMs that go unnoticed using peak-based approaches suggesting that a large part of the *regulome* might be uncharted water.

## INTRODUCTION

Elucidating the diversity of transcriptional regulation is a prevalent problem in computational biology. While there is a plethora of mechanisms involved in regulating transcription (1), especially the binding of Transcription Factors (TFs) to regulatory elements (REMs) such as *Promoters*, *Enhancers* and *Repressors* has been shown to be essential for orchestrating cellular development and identity (2,3). Importantly, enhancers have been closely linked to several diseases and recent research suggests that enhancers might be therapeutic targets (3,4).

In order to describe how REMs might influence their target genes in a systematic way, two models have been proposed: the scanning model and the looping model (3,5). According to the scanning model, a REM is usually affecting a gene that is located in close genomic distance, whereas in the looping model, REMs can influence a gene that is located several kilobases (kb) away from the actual regulatory site via chromatin looping. Because biological evidence has been found for both models, it is likely that both do occur *in-vivo* (6,7).

To elucidate regulatory function, two main problems need to be solved: Firstly, REMs, need to be identified genome wide and secondly, they need to be assigned to their target genes. The first problem, identifying REMs genome wide, has been addressed by international projects, e.g. Blueprint and Roadmap. There, REMs were identified using DNase1-Hypersensitive Sites (DHS), i.e. sites of accessible chromatin (8,9), via distinct patterns of Histone Modifications (HMs), i.e. the co-occurrence of H3K27ac and H3K4me1 while H3K4me3 is absent (10), or via TF-ChIP-seq experiments of TFs such as EP300 (11). Typically, such data sets are analysed with peak calling algorithms. Although, there is a plethora of peak callers available, designed for ChIP-seq (12) and chromatin accessibility data (13), peak callers still have several limitations. For instance, the selection of the cut-off to determine peaks over background is not trivial, and also cell cycle stage (14) or cell numbers (15) can prevent the accurate detection of truly enriched regions. Furthermore, it is often not clear what level of enrichment is needed such that a region can be seen as biologically active (16). Besides, as illustrated in Supplementary Figure S1, integrating peak calls across several diverse samples is not straightforward (17). However, an integrated set of peaks is required if machine learning approaches should be utilized to associate a defined set of candidate REMs to potential target genes across many samples. Note that automated integration of replicates, as offered e.g. in the peak caller JAMM (18), is not designed for such an application. It is rather meant to provide stable, reproducible peak calls across replicates of the same cell-type or tissue.

In addition to the efforts taken by Blueprint, Roadmap and other IHEC members, putative enhancers were identified in the Fantom5 consortium via the identification of distinct bidirectional expression patterns in CAGE (Cap Analysis of Gene-Expression) data (19).

Overall, many different ways have been proposed to identify putative REMs using distinct chromatin signatures. Nevertheless, the problem of linking those regions to the genes they regulate is still not straightforward to solve. In literature, especially in instances were only few replicates are available, putative REMs are often linked to their nearest gene according to genomic distance (20), or aggregated using window based approaches (21–23). However, several studies suggest that especially enhancers and repressors do not regulate their nearest gene but may influence more distant genes (19,24–26). On top of that, REMs are highly tissue-specific (27), suggesting that a purely distance based detection of REMs is error prone.

Yao *et al.* (3) describe two approaches attempting to overcome these limitations: (i) methods based on physical interaction, i.e. capture Hi-C (28), or Chromatin Interaction Analysis by Paired-End Tag sequencing (ChIA-PET) (29) and (ii) methods based on associating gene-expression to the activity of REMs, e.g. using DNase1-seq (9,24), or HM abundance (30). Further, Hi-C data can be combined with open-chromatin and histone ChIP-seq data to predict enhancer-gene interactions (31).

While methods based on physical interaction are laborious, time consuming and experimentally challenging, e.g. in terms of providing a sufficient resolution of long-range contacts (32), association based methods are predestined to use the plethora of available epigenetics data to link REMs to their target genes: Using machine learning, Cao *et al.* propose to integrate predicted REMs into cell-type specific interaction networks (33), similar to Hait *et al.*, who also provide regulatory-maps derived from statistical associations between the activity of REMs and target gene-expression (24). Shooshtari *et al.* combined chromatin accessibility data with Genome-Wide Association Studies (GWAS) to better pinpoint regulatory events in autoimmune and inflammatory diseases (34). In the Fantom5 consortium, putative REMs have been linked to their target genes by associating enhancer activity to gene-expression (19). Gonzales *et al.* use a nearest gene linkage of DHSs in an iterative manner within gene-expression models to link REMs to their target genes (20).

**Figure 1.**
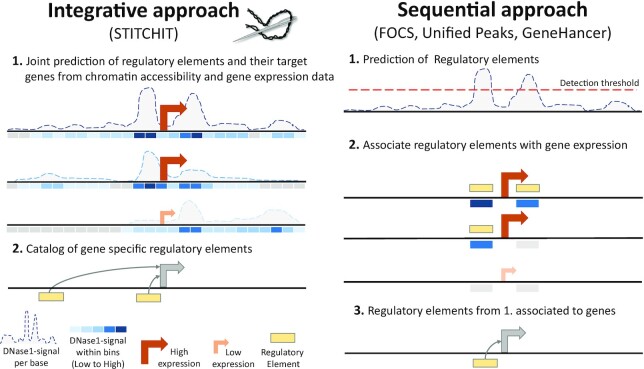
Comparison of REM inference approaches. (right) Current methods solve the problem of linking REMs to target genes with a sequential approach (e.g. FOCS). Firstly, a catalogue of putative REMs is defined using peak calling. Secondly, signal in REMs in a window around a gene is associated to the expression of the same gene. The final associations are reported (3). (left) STITCHIT combines the step of defining REMs and their association to target genes into a joint prediction problem (1) and generates a catalogue of gene-specific REMs (2.). Thereby REMs that zoom in on the epigenomic region that is associated with gene-expression are generated. This allows to detect more subtle REMs that are missed by sequential approaches that apply strict thresholds on the initial signal.

Here we present StitchIt, a novel method to identify and to link REMs to their target genes. Unlike conventional approaches, that are either using peaks or literature curated sets to identify candidate REMs, StitchIt solves the problems of identifying and linking REMs to genes simultaneously instead of solving two independent sub-problems (Figure 1). Applying StitchIt to two large datasets obtained from Blueprint and Roadmap shows that our peak-free strategy outperforms the state of the art REM inference and linkage methods in various quality control experiments. Using CRISPR-Cas9 experiments, performed in an unseen cell-line, we were further able to validate the regulatory role of novel REMs detected by StitchIt.

## MATERIALS AND METHODS

### Preprocessing

Paired DNase1-seq and RNA-seq data was downloaded for 110 Roadmap samples. Upon granted access, we obtained 56 paired DNase1-seq and RNA-seq samples from Blueprint. An overview on sample numbers and tissue/cell-type diversity is provided in Table 1. Supplementary Table S1 lists all data accession numbers.

**Table 1.** Overview on the data used in this study

	Blueprint	Roadmap
#Paired samples	56	110
#Different cell-types	13	33
Primary cells only	Yes	No

Paired samples are required as they are expected to have a better correlation between chromatin structure and gene-expression, because both samples originate from the same donor. Details on data processing as well as used command calls are provided in Supplementary Section 1.

Further, we obtained H3K27ac, H3K4me1 and H3K4me3 data in *wig* format from the Blueprint data portal for four samples (C0011IH1, S00C0JH1, S00XUNH1, C0010KH1, see Supplementary Table S1). Also, we downloaded REMs contained in the GeneHancer database from the GeneLoc website (35).

### Overall workflow and conceptual idea

Conceptually, we pursue the idea to identify regions in large genomic intervals around a gene *g* of interest that can be associated to the expression variation of gene *g* across many samples. To identify these regions, we utilize paired epigenetics and gene-expression data. The StitchIt algorithm uses the actual signal of the epigenetics data to highlight segments of the data showing signal variation that can be used to separate samples according to the target genes expression. Thus, the peak-calling step can be omitted and the two tasks of identifying regulatory sites and their linkage to targets are solved simultaneously. To refine the list of putative REMs identified by StitchIt, we apply a regression approach that is detailed below. This allows us to judge the explanatory power of the found regions for gene-expression and to assess the significance of each identified region. The workflow of the proposed methodology is depicted in Figure 1, details are provided in Supplementary Figure S2.

### Discretization of gene-expression data

In this work, we used the Probability of Expression (POE) method to discretize gene-expression data (36). Briefly, POE determines for each gene a discrete expression state *c* ∈ *C* by fitting a mixture model composed of either two classes (*c*_1_ (expressed) versus *c*_2_ (not expressed)) or three classes (*c*_1_(less expressed than baseline), *c*_2_(baseline expression), *c*_3_(higher expressed than baseline)), depending on which model achieves a higher likelihood. While there is a R-implementation of POE available, we had to adapt it for compatibility reasons. The updated R-Code is provided in Supplementary Section 2.

### The STITCHIT algorithm

In the following, we are given a dataset *D*_*g*_ with *m* rows, corresponding to the samples, and *n* columns representing the epigenetic signal at base pair resolution around the target gene *g*. Further, to each row, we assign a class label, indicating whether the corresponding sample is associated with a high, medium or low expression value (*C* = 0, 1, 2). Note that also a two-level classification was used here (*C* = 0, 1), depending on the results of the POE method (36) (cf. Supplementary Section 2). The algorithm is implemented such that any number of distinct class labels, not exceeding the number of samples, (|*C*| ≤ *m*) can be used. With *C*_*k*_ we relate to all rows to which we assigned class label *k* ∈ *C*.

A segment *s* has a start point *i* and an end point *j*, where 1 ≤ *i* ≤ *j* ≤ *n*. We call *S*_*g*_ a segmentation of *D*_*g*_, if it contains a set of non-overlapping segments that covers the whole range from 1 to *n*. The two trivial cases would be a segmentation consisting of only a single segment with start point *i* = 1 and end point *j* = *n* or the segmentation containing *n* segments, where each segment only contains a single column, *i.e*. a single base. The former would contain no information about the class labels, while the latter would consist of a large set of noisy segments which result in bad features for the learning step that is based on the segmentation. Our goal is to provide a small set of robust features for the learning step. We achieve this by joining adjacent base pairs to segments, such that the variance between the epigenetic signals of base pairs that are contained in a segment is low, w.r.t. the class label. The optimal segmentation according to the score we define below, finds a trade-off between the number of segments and the variance.

To score a segmentation, we propose an information theoretic score based on the Minimum Description Length (MDL) principle (37). MDL is a practical instantiation to Kolmogorov complexity (38) and thus belongs to the class of compression-based scores. Formally, given a model class $\mathcal {M}$, MDL identifies the best model $M \in \mathcal {M}$ for data *D* as the one minimizing

(1) $$\begin{equation*} L(D,M) = L(M) + L(D \mid M) , \end{equation*}$$

where *L*(*M*) is the length in bits of the description of the model *M*, and *L*(*D*∣*M*) is the length in bits of the description of the data *D* given *M*. This is known as two-part, or crude MDL. In essence, we try to find the simplest model that can explain the data well. We follow the convention that all logarithms are base two, since the length of the encoding relates to bits, and define 0log 0 = 0. In this work, we use MDL to balance our segmentation between having too few segments and running at risk of missing structure in the data and finding too many segments, which contain spurious information and make the post-processing infeasible.

From now on, we consider the model class of segmentations $\mathcal {S}$ from which we want to find the optimal segmentation $S_g^{{\it opt}}$, that is

(2) $$\begin{equation*} S_g^{{\it opt}} = {\rm arg\, min}_{S_g \in \mathcal {S}} L(S_g) + L(D_g \mid S_g) . \end{equation*}$$

In particular, we encode a segmentation *S*_*g*_ as follows:

(3) $$\begin{eqnarray*} L(S_g) &=& L_{\mathbb {N}}(|S_g|) + |S_g| |C| \log \left(\frac{| \max - \min |}{\tau } \right) \nonumber \\ && +\, \log { \binom{n-1}{|S_g|-1}} , \end{eqnarray*}$$

where |*S*_*g*_| denotes the number of segments, $L_{\mathbb {N}}$ is the universal prior for integer numbers (37), |*C*| is the number of class labels, *max* refers to the maximum value observed in the data, *min* refers to the minimum value observed in the data and τ ≤ 1 is the data resolution. The τ parameter is used to fix a certain precision up to which we record the data. This is necessary to fairly compare models when dealing with floating point numbers.

First, we encode the number of segments, then for each segment per category the associated mean value by assuming it lies between the minimum and the maximum value in the data and last the complexity to select |*S*_*g*_| segments from possible *n* segments.

To encode the data given a segmentation, we simply sum over the costs per segment

(4) $$\begin{equation*} L(D_g \mid S_g) = \sum _{s \in S_g} \sum _{k \in C} \frac{1}{|C_k|} L(D_g \mid s,k) , \end{equation*}$$

where |*C*_*k*_| corresponds to the number of rows associated with class label *k*. Here, *costs* or *encoding costs* refers to the code length per segment. The longer the encoded length, the higher the costs of encoding a segment.

To encode the costs for a specific segment and the data associated with class *k*, we encode the error assuming a Gaussian distribution. Using $\hat{\sigma }^2$ as the sample variance over the data corresponding to segment *s* and class label *k*, we get (compare (37))

(5) $$\begin{eqnarray*} L(D_g \mid s, k) &=& \frac{|s||C_k|}{2} \left( \frac{1}{\ln (2)} + \log (2 \pi \hat{\sigma }^2) \right) \nonumber \\ && +\, |s||C_k| \log \tau , \end{eqnarray*}$$

with |*s*| being the length of the segment. Note that the epigenetic data is not discrete, but continuous. To model the epigenetic signal probabilistically, we assume that those data points that fall within a single segment are Gaussian distributed. That is, to specify the model for the data of one class in one segment, we need to specify the mean and the variance. Specifically, the squared loss penalizes data points that are further away from the mean more than for example measuring the absolute error. Consequently, the more deviating the mean and variance of two adjacent segments are, the more costly and hence the less likely it would be to merge these together into one segment.

To find the optimal segmentation $S_g^{{\it opt}}$, we use dynamic programming (39). In essence, we start with a segmentation containing only a single segment. Then we iteratively compute the best segmentation containing *i* segments based on the best segmentation containing *i* − 1 segments for *i* ∈ {2, …, *n*}. Lastly, we select $S_g^{{\it opt}}$ among the optimal segmentations for each possible number of segments. The runtime complexity of this algorithm is $\mathcal {O} (n^2)$. By selecting a minimum segment size of β and partitioning the search space into *l* chunks, we can run each chunk in parallel and the total runtime complexity reduces to $\mathcal {O} (\frac{n^2}{l\beta ^2})$. In our experiments, we use β = 10 and set *l* to $\lceil \frac{n}{5000} \rceil$, which makes the algorithm feasible to be applied on large genomic intervals. Here, we have considered 25*kb* upstream of a genes’ Transcription Start Site (TSS) and 25 kb downstream of a genes’ Transcription Termination Site (TTS).

An example is provided in Supplementary Section 3.

### Selection of candidate regulatory elements

Those segments that are associated to the observed expression changes need to be extracted from $S_g^{{\it opt}}$. Thus, for all segments $s \in S_g^{{\it opt}}$ we compute both Pearson and Spearman correlation between the epigenetic signal in *s* across all samples *m* and the continuous expression values of the target gene *g*. We select all segments that achieve a correlation value (Spearman (default), or Pearson) with a significance threshold of *P* ≤ 0.05. We apply the same filtering to the alternative methods introduced below.

### Refinement of selected regions using linear regression

StitchIt provides for all selected segments $s \in S_g^{opt}$ a matrix *X* holding the epigenetic signal within these regions. The *m* rows of *X* denote the samples, the *n* columns refer to the regions selected by StitchIt. To further refine the suggested regions for a distinct gene *g*, we first train a linear model using elastic net regularization, as implemented in the glmnet R-package (40). Here, we are utilizing the DNase1-seq signal within candidate REMs (*X*) to predict the expression of *g*, stored in *y*. The grouping effect results in a sparse regression coefficient vector. However, correlated features, i.e. regions that jointly regulate gene *g*, which is an expected scenario in this application, will be maintained. This is achieved by combining both the Ridge and the Lasso regularizers:

(6) $$\begin{equation*} \hat{\beta }=\underset{\beta }{\arg \, min} ||y-X\beta ||^2 + \lambda [\alpha ||\beta ||^2 + (1-\alpha )||\beta ||] . \end{equation*}$$

Here, *β* represents the feature coefficient vector, $\hat{\beta }$ the estimated regression coefficients, and λ controls the total amount of regularization. Both the input matrix *X* and the response vector *y* are log-transformed, with a pseudo-count of 1, centered and normalized. The parameter α, which is optimized in a grid search from 0.0 to 1.0 with a step-size of 0.01 controls the trade-off between Ridge and Lasso penalty.

As previously performed by Schmidt *et al.* (41), model performance is assessed in terms of Pearson and Spearman correlation as well as using the mean squared error (MSE) between predicted and measured gene-expression. Specifically, the performance of the linear model is assessed on an hold-out test dataset in a ten-fold outer Monte Carlo cross-validation procedure, where $80\%$ of the data are randomly selected as training data and $20\%$ as test data. The parameter λ is fitted in a six-fold inner cross-validation using the *cv.glmnet* procedure. The parameters’ final value is determined according to the minimum cross-validation error, which is computed as the average MSE on the inner folds (*lambda.min*).

Significance of the correlation between predicted and measured gene-expression is corrected using the Benjamini-Yekutieli correction (42), which is designed to account for dependency between the tests (24). Only models with a *q*-value ≤0.05 are considered for interpretation of the selected regions. For those models, we refer to all features with a median non-zero regression coefficient across the outer folds by *X*_*NZ*_.

In a second learning step, similar to Hait *et al.* (24), we train an Ordinary Least Squares model (OLS) on the pre-selected features *X*_*NZ*_ predicting *y* and report the regression coefficients β_*OLS*_ as well as the *P*-values per feature for downstream analysis:

(7) $$\begin{equation*} y=X_{NZ}\beta _{OLS}. \end{equation*}$$

The OLS model allows for a simple comparison of regression coefficients β_*OLS*_ across genes, as there is no bias introduced by the regularization, and provides a straight forward way to compare individual regions. Note that the OLS model is not used to judge model performance. Model performance is exclusively assessed using the cross-validation procedure described above. All regions and model coefficients used for interpretation and validation are obtained from the OLS models (Supplementary Figure S3a).

### Nested execution of STITCHIT inside a Monte Carlo cross-validation procedure

In addition to the aforementioned pipeline that uses the same data set for the execution of StitchIt and as input for the linear models to refine the REM selection, we devise a nested Monte Carlo cross-validation strategy that considers $80\%$ of the data to generate the set of candidate REMs $S_g^{opt}$ for gene *g*. The exact same $80\%$ of the data are subsequently used to fit the elastic net model as described above. The performance of the elastic net model is then evaluated on the $20\%$ of unseen data, which have not been used in generating the set of candidate REMs $S_g^{opt}$. To obtain a robust performance estimate this Monte Carlo cross-validation is repeated 10 times per gene *g*. A graphical overview on the nested execution of StitchIt is provided in Supplementary Figure S3b.

### Down-sampling of training data

To perform down-sampling experiments we use a nested cross-validation strategy using $20\%$ of the complete Roadmap data set for model testing and down-sampled versions of the remaining $80\%$ for training. Specifically, from these $80\%$ of the data, we generate down-sampled sets considering $40\%$, $50\%$, $60\%$, $70\%$, $80\%$, $90\%$, and $100\%$ of the data points for model training. For each gene, we repeat this process 10 times in a Monte Carlo fashion randomly selecting the test and training samples. StitchIt and REM refinement are performed as described above.

### Alternative approaches to identify and to link REMs to genes

We compare the REMs identified with StitchIt ($\mathcal {S}$) to those obtained with three alternative approaches (Supplementary Figure S4): (i) an unsupervised, window based aggregation of DHSs per gene and per sample, (ii) taking the union of DHSs across all samples (UnifiedPeaks) and (iii) considering known REMs from the GeneHancer database. Command line arguments along with further details on how to produce the respective scores are provided in Supplementary Section 4. We applied exactly the same linear regression paradigm for approaches (ii) and (iii) as described above for the regions identified with StitchIt. The unsupervised linkage (1) is not considered for interpretation purposes.

#### Unsupervised integration of peaks per sample

Similar to work by others (20,41), we determine for each gene *g* in each sample *i* considering a predefined window $w$ how many DHSs are located within this window $c^g_i$, how long the accessible regions $l^g_i$ are and we aggregate the signal intensity within the selected DHSs $s^g_i$. The contribution of each DHS *p* is also weighted by its distance *dist*(*p*, *g*) to the TSS of gene *g* following an exponential decay. Details are provided in Supplementary Section 4.

#### Unified peaks

Here, we generate consortia specific aggregations of all DHSs called with JAMM. Overlapping sites are merged using the BEDTools*merge* command. Thereby, we obtain a set of regions representing all accessible sites within one dataset. Using the *bigwig* files generated with DEEPTools and the *libBigWig* library (https://zenodo.org/record/45278), we compute the DNase1-seq signal within the merged peaks for each sample. Next, we test for all candidate peaks within a distinct window $w$, here $w$ = 25 kb upstream of a genes TSS and downstream of its TTS, whether there is a significant spearman correlation (*P* ≤ 0.05) between the DNase1-seq signal within the peak and the expression of the gene. All merged peaks passing this test ($\mathcal {U}$) are considered for the regression model described above. We refer to this as the UnifiedPeaks approach.

This approach is conceptually similar to the peak aggregation approaches pursued by Hait *et al.* (24) and Shooshtari *et al.* (34).

#### GeneHancer

For all REMs obtained from the GeneHancer database, we calculate the sample specific DNase1-seq signal within each region for each gene, using the *libBigWig* library. Note that a window or distance cut-off is not required here since each region is already assigned to its putative target gene. Considering that the GeneHancer database is comprised of REMs originating from many different sources identified with a plethora of assays and molecular signatures, we perform the same correlation based test as above to identify a subset ($\mathcal {G}$) of regions with sufficient correlation between the DNase1-seq signal and the gene-expression of the respective target gene.

### Validation of putative regulatory regions

#### Overlap with the Ensembl Regulatory Build and OCHROdb

We used the terms: Open chromatin, Promoter, Promoter Flanking Region, TF binding site and Enhancer from the Ensembl Regulatory Build (ERB) (43) (release 86), to compare predicted REMs to an established regulatory annotation of the genome. To further refine the analysis, we compare REMs not overlapping any ERB terms with the DHSs contained in OCHROdb (44), a manually curated database of reproducible DHSs across replicates and various consortia within IHEC.

#### Chromatin accessibility and regulatory relevance of previously unknown REMs

We further assessed the DNase1 signal within REMs overlapping any of the ERB terms or the OCHROdb (labelled as *known*) and those not overlapping these elements (labelled as *unknown*). We compared their DNase1 signal against 10 000 randomly chosen genomic regions using the BEDTools*shuffle* command excluding the original positions.

Furthermore, we investigated whether the top REM per gene is a *known* or an *unknown* REM. Also, we performed a simple enrichment test for each gene, using the gset function from the gsEasy package considering the sorted list of REMs (by absolute regression coefficient) and the label of each REM (*known*, *unknown*).

#### Overlap with histone modification data

We selected the top 10 000 StitchIt REMs, ranked by their OLS *P*-values. Additionally, we have randomly chosen 10 000 StitchIt REMs from the entire set and, as a baseline, obtained 10 000 random regions of similar size using the BEDTools*shuffle* command excluding the original positions. Next, we obtained the H3K27ac, H3K4me3 and H3K4me1 signal for four Blueprint samples (see Data) in 1*kb* windows centered in the middle of the candidate REMs and visualized the data in r. Furthermore, we obtained the top 10 000 StitchIt REMs for each class of labels used in the Regulatory build and assessed the H3K27ac signal within those REMs.

#### Overlap with GeneHancer

Using BEDTools*intersect* we computed the overlap between all candidate regulatory sites identified with StitchIt with all unique entries contained in the GeneHancer database that are within the searched 25 kb search window and downstream of each gene (193 298 distinct regions). The same is done for regions based on the UnifiedPeaks approach, thereby assessing how many known REMs from GeneHancer can be recovered.

#### GWAS hits

We collected 103 121 unique GWAS sites from the European cohort contained in the EMBL-EBI GWAS Catalog (45). For these SNPs we determined 1 020 896 proxy SNPs using the precomputed data of the European population provided by SNiPA (46). The collection of the SNPs from the EMBL-EBI GWAS Catalog combined with the proxy SNPs is denoted as $\mathcal {M}$. For all gathered SNPs we looked up their Minor Allele Frequency (MAF) provided by the dbSNP database (47) (build 154). Next, we computed 100 randomised SNP sets. Therefore we sampled for each set $|\mathcal {M}|$-many SNPs from the dbSNP database, while maintaining the MAF distribution of $\mathcal {M}$. The sets of random SNPs are denoted as $\mathcal {A}=\lbrace \mathcal {A}_1,...,\mathcal {A}_{100}\rbrace$.

Next, we computed three different measures to characterize the overlap between STITCHIT REMs ($\mathcal {S}$) and our GWAS catalog ($\mathcal {M}$): (i) $|\mathcal {S} \cap \mathcal {M}|$, denoting how many overlaps occur between any $m \in \mathcal {M}$ and any candidate StitchIt REM $s \in \mathcal {S}$ and $|\mathcal {S} \cap \mathcal {A}|$, denoting the expected number of such overlaps using the random SNP sets $\mathcal {A}$; (ii) $|\lbrace m: m \in \lbrace \mathcal {S} \cap \mathcal {M}\rbrace \rbrace |$, denoting the number of unique GWAS loci $m \in \mathcal {M}$ overlapping with any candidate StitchIt REM $s \in \mathcal {S}$ and $|\lbrace a : a \in \lbrace \mathcal {S} \cap \mathcal {A}\rbrace \rbrace |$, denoting the expected number of unique SNPs; (iii) $|\lbrace s : s \in \lbrace \mathcal {S} \cap \mathcal {M}\rbrace \rbrace |$, denoting the number of unique StitchIt REMs overlapping any $m \in \mathcal {M}$ and $|\lbrace s: s \in \lbrace \mathcal {S} \cap \mathcal {A}\rbrace \rbrace |$, denoting the expected number of unique REMs.

#### Generation of a REM background model

We generated REM background sets specific for StitchIt, UnifiedPeaks and GeneHancer matching the number and length of REMs per gene. Here, we follow the established assumption that REMs are more likely to be placed close to the TSS of their target gene than far away from it. For each gene, we generated as many REMs upstream and downstream of the TSS as present in the original REM sets. We computed REM positions using the rexp function sampling from an exponential distribution with a rate parameter of 7.

#### eQTL analysis

We obtained uniformly reprocessed BLUEPRINT eQTLs $\mathcal {B}$, including three different primary cell types, and GTEx version 8 eQTLs $\mathcal {T}$, including 49 different tissues from EMBL’s eQTL catalogue (48) (Supplementary Table S10).

We count how many REMs overlap eQTLs that are assigned to the same gene as the REM, in other words, we compared the gene-locus assignment from all $b \in \mathcal {B}$ and $t \in \mathcal {T}$ with our predicted REMs $\mathcal {R}=\lbrace \mathcal {S},\mathcal {U},\mathcal {B}\rbrace$ and obtained the number of REMs with correct overlaps $O_\mathcal {R}^\mathcal {B}$ and $O_\mathcal {R}^\mathcal {T}$, respectively. To assess the significance of this overlap, we compared it to the REM background models based on exponential decay described above, denoted by $\mathcal {E}_i$ with *i* ∈ [1, 10], approximating the expected overlap denoted by $E_\mathcal {R}^\mathcal {B}$ and $E_\mathcal {R}^\mathcal {T}$, respectively. The observed over expected ratio $OE_\mathcal {R}^\mathcal {B}$ and $OE_\mathcal {R}^\mathcal {T}$ can be computed by

(8) $$\begin{eqnarray*} OE_\mathcal {R}^\mathcal {B}=\frac{O_\mathcal {R}^\mathcal {B}}{\underset{i \in [1,10]}{\mathrm{mean}}(\mathcal {E}_i^\mathcal {B})}, \end{eqnarray*}$$

(9) $$\begin{eqnarray*} OE_\mathcal {R}^\mathcal {T}=\frac{O_\mathcal {R}^\mathcal {T}}{\underset{i \in [1,10]}{\mathrm{mean}}(\mathcal {E}_i^\mathcal {T})}. \end{eqnarray*}$$

#### ChIA-PET and Capture Hi-C data

ChIA-PET data $\mathcal {P}$ for K562 and MCF-7 targeting the RNA polymerase II was downloaded from the 4DGenome database (49) and lifted to *hg38* using the UCSC liftover tool. The ChIA-PET data sets contain 64 773 and 65 269 interactions, respectively. Promoter capture Hi-C data $\mathcal {C}$ for GM12878 was obtained from Mifsud *et al.* (50) and also lifted to *hg38*. The GM12878 Promoter Capture Hi-C data set contains 88 568 interactions. In addition, we obtained Promoter Capture Hi-C data from Javierre *et al.* (51), which was generated in scope of the Blueprint project and hence matching well to our Blueprint data set. The Blueprint Promoter Capture Hi-C data set contains 51,142 interactions. The chromatin interaction data allows us to calculate how many REMs $\mathcal {R}=\lbrace \mathcal {S},\mathcal {U},\mathcal {B}\rbrace$ and target gene interactions match the chromatin contacts captured by the ChIA-PET $\mathcal {P}$ or Promoter Capture Hi-C $\mathcal {C}$ data. To match chromatin interaction data to our suggested REMs, we consider the entire gene-body of the linked gene as the second coordinate. We consider the entire gene-body to (i) easily cover interactions to alternative transcription start sites and (ii) to account for regulatory interactions within the gene body as reported before (20). We count an overlap as valid if either the gene or the coordinate of the associated REM overlaps one coordinate of the chromatin interaction and the second coordinate of the interaction site overlaps the remaining coordinate of the association.Valid overlaps are denoted as $O_\mathcal {R}^\mathcal {P}$ and $O_\mathcal {R}^\mathcal {C}$, respectively. As for the eQTL analysis, we calculate an expected number of overlaps using the method specific REM background sets denoted as $E_\mathcal {R}^\mathcal {P}$ and $E_\mathcal {R}^\mathcal {C}$, respectively. The observed over expected ratio $OE_\mathcal {R}^\mathcal {P}$ and $OE_\mathcal {R}^\mathcal {C}$ can be computed as

(10) $$\begin{eqnarray*} OE_\mathcal {R}^\mathcal {P}=\frac{O_\mathcal {R}^\mathcal {P}}{\underset{i \in [1,10]}{\mathrm{mean}}(\mathcal {E}_i^\mathcal {P})}, \end{eqnarray*}$$

(11) $$\begin{eqnarray*} OE_\mathcal {R}^\mathcal {C}&=\frac{O_\mathcal {R}^\mathcal {C}}{\underset{i \in [1,10]}{\mathrm{mean}}(\mathcal {E}_i^\mathcal {C})}. \end{eqnarray*}$$

To assess both the distance of (not) supported REMs to the TSS of their target gene as well as the regression coefficient of (not) supported REMs, we decided to only consider REMs that are likely to be active in the cell lines used to generate the confirmation capture data as this would be a more meaningful comparison. Here, we define a REM as active if it has a non-zero DNase1-seq signal. To do so, we used DNase1-seq data for K562 (ENCFF971AHO) and MCF7 (ENCFF924FJR) to complement the ChIA-PET data, and DNase1-seq data for GM12878 (ENCFF743ULW) to complement the Capture Hi-C data.

### Analysis of additive enhancers

Anderson *et al.* defined redundant enhancers as REMs that have a contribution to the model of at least 0.2 and that are highly correlated (Pearson correlation > 0.7) with any other of the nine enhancers they considered in their model. They observed that with an increasing number of redundant enhancers, the maximum expression of their target genes increases, thus they call those enhancers *additive* enhancers (52). Here, as our setup is different, e.g. we are not limited to ten enhancers per model, we compute the Spearman correlation between all enhancers that pass the elastic net regularization and are used in the OLS model. We consider these enhancers as redundant if their Spearman correlation is >0.8. Due to the possible zero inflation of the read data, we use Spearman instead of Pearson correlation. The maximum expression of the related genes is assessed for genes in groups considering genes with [0,1], [2,3], [4,5] and [6[ redundant enhancers, respectively.

### Comparison against REMs determined by FOCS

We obtained promoter enhancer interaction (PEIs) predictions computed by Focs from the methods website at http://acgt.cs.tau.ac.il/focs/download.html and downloaded files using data for Roadmap (Roadmap Epigenomics Enhancer-Promoter links with annotations), as these are the PEIs most comparable to StitchIt data. As Focs predictions are only available for *hg19*, we used the USCS liftover tool to convert them to *hg38*. Specifically, we converted both promoter and enhancer coordinates. Next we concatenated regulatory information from the PEI lists for promoters and enhancers per gene to obtain a REM format comparable to that of StitchIt. This resulted in 105 379 Focs REMs for Roadmap data. Using these lists we repeated the validation experiments described above regarding the overlap with gRNAs, eQTLS from the ExSNP database, GWAS hits and ChIA-PET data.

### Characterization of repressors and multi target REMs

To identify REMs targeting multiple genes and to characterize the nature of the regulatory influence, we merged overlapping REMs using the BEDTools*merge* command generating a set of *Union REMs*, called CREMs. For these union sets, we used the BEDTools*intersect* command to determine which REMs target exactly one and which target more than one gene (multi target). For multi target REMs, we determined whether they constantly have a positive, negative or both associations. We tested whether the observed trends depend on the number of target genes or on the absolute value of the regression coefficients. Additionally, we randomly shuffled the OLS regression coefficients assigned to the REMs ten times to generate a background distribution. To perform motif enrichment, as described in the next section, we extract the sequence of those REMs that have exclusively either a positive or a negative association.

### Motif enrichment analysis

To identify key TFs within a REM sequence set of activators and repressors (r.f. the previous section), we performed a motif enrichment analysis. Therefore, we downloaded the binding motifs of 515 human TFs from the JASPAR database (53). We used TRAP (54) to compute for each sequence and each TF a TF-affinity value, which is defined as the sum over all binding site probabilities of a given TF for a sequence. In addition, we created a background sequence set consisting of randomly picked genomic regions, which are not overlapping with the original REM sequences, are of the same length and from the same chromosome as the original REM sequences. We also applied TRAP on this background sequence set. Based on these TF-affinities, we performed a one-sided MannP–Whitney test to identify TFs, which are enriched over all REM sequences in comparison to the background sequence set. We adjusted the resulting *P*-values (using Benjamini–Hochberg procedure) and considered all TFs as significant with an adjusted *P*-value smaller or equal than 0.001.

### Splitting peaks through STITCHIT

From the overlap between UnifiedPeaks ($\mathcal {U}$) and StitchIt ($\mathcal {S}$) regions it can be computed into how many StitchIt segments *s* a peak $p \in \mathcal {U}$ is split into. We refer to the instance that *p* is divided into several segments *s* as a *split event*. The *degree of a split event* denotes the number of StitchIt segments *s* a peak $p \in \mathcal {U}$ is segmented into. Within this counting procedure we also impose that any *s* overlapping *p* needs to be linked to a different gene *g* than *p*, while any StitchIt segment *s* can be assigned to the same target gene *g*′ as long as *g*′ ≠ *g*. In addition, we quantify how many *split events* are supported by conformation data. To this end, for each *split event*, we assess how many StitchIt segments overlap a matching genomic contact obtained from ChIA-Pet or Capture Hi-C data. If all StitchIt regions are supported, we call a split *fully supported*, if not all but at least one region is supported we call it *partially supported*. To ensure that UPs are not split into different REMs due to over fitting of the StitchIt model, we also counted the number of times a peak $p \in \mathcal {U}$ is split into multiple StitchIt segments *s* that are linked to the same gene *g*. Also, we computed the median length of peaks *p* involved in split events, separately for different split event degrees.

As an orthogonal way of validating split events, we computed the overlap of REMs involved in split events to superenhancers contained in the superenhancer database (SEdb) (55). Specifically, we calculate a ratio score:

(12) $$\begin{equation*} r=\frac{S_{SEdb}}{|SEdb|}, \end{equation*}$$

where *S*_*SEdb*_ denotes the number of distinct StitchIt REMs overlapping an entry of the SEdb and |*SEdb*| refers to the total number of entries in the SEdb. As the SEdb contained overlapping elements, we used BEDTools*merge* to unify overlapping entries resulting in a total of |*SEdb*| = 142 637 SE elements, which are used for the overlap computation. We compared *r* to a background score

(13) $$\begin{equation*} r^{\prime }=\frac{1}{10}\sum \limits _{i=[1,10]}\frac{S_{SEdb}^{\prime }}{|SEdb|}, \end{equation*}$$

where $S_{SEdb}^{\prime }$ is based on ten random shufflings of the original REMs throughout the genome maintaining the distribution per chromosome.

### CRISPR-Cas9 experiments to validate REMs suggested by STITCHIT

Here, we describe a general approach for the experimental design of targeted CRISPR-Cas9 experiments using our REMs. Using ENCODE DNase1-seq data for Human Umbilical Vein Endothelial Cells (HUVECs) (*ENCSR000EOQ*) we compute the activity of predicted REMs for each gene using the StitchIt C++ module *REMSELECT*, which is part of the github repository. Given a custom bigWig file and predicted REMs for a gene as input to *REMSELECT*, it generates a tabular overview of REM position, regression coefficient, chromatin accessibility readout, OLS *P*-value and a combined score multiplying the regression coefficient with the signal abundance in the respective REMs from the bigWig file. This score allows us to rank REMs simultaneously by the predicted REM relevance and the activity of the REMs in the cell type/ cell line of interest. Based on our REM activity score, gene-expression of the target genes in HUVECs, existing H3K27ac signal (*ENCSR000ALB*) within REMs and our ability to find gRNAs for a CRISPR-Cas9 experiment, we decided to validate REMs for three genes: KLF2 (A), NOS3 (B) and AC020916 (C).

We designed paired gRNAs to achieve a genomic deletion for one REM per gene. In a first step, we used a webtool (56,57), which is based on the Azimuth2.0 algorithm to determine gRNAs within a 200 bp range around the REMs of the considered genes. Next, we applied *Cas-OFFinder* (58) to eliminate the gRNAs with any off-targets. From the remaining ones, we choose for each gene one gRNA pair that cuts out the corresponding REM most precisely. Supplementary Table S2 shows the position of the considered REMs, the locations of the gRNA binding sites and the position of the deleted genomic region per gene. Upon gRNA design, circular plasmids harboring two gRNAs per REM for each gene (A–C) and Cas9 were synthesised with the 3Cs method (59) on plasmid p0023.dna. Successful synthesis was verified by Sanger sequencing. Four viruses were generated with plasmids (A, B, C) and an empty control plasmid p23 following the protocol of Wegner *et al.* (59). Titer was determined in puro-sensitive RPE1 cells without Cas9 (59) (A: 1 × 10^6^, B: 3 × 10^6^, C: 1 × 10^6^, Control: 2 × 10^5^). At D0 400,000 (66 000 cells per well) HUVECs from LONZA were seeded on a 6-well dish using EGM medium from PELOBiotech (Cat. PB-SH-100-2199, PB-BH-100-9806). Cells were transduced on D1 with virus and MOI=1 (polybrene 8μ*g*/*ml*) in three independent replicates for each sample. At D3 cells were washed 5× with PBS and harvested after 48h. RNA was purified using Rneasy Plus Mini Kit Cat. No. 74134 (Qiagen) according to protocol. The High-Capacity cDNA reverse transcription Kit 4368814 (Life Technologies) has been used to generate cDNA according to protocol. PCR was performed as shown in Supplementary Table S3, PCR-Primers are provided in Supplementary Table S4. We loaded 50 μl PCR sample and 10 μl loading dye on $1.5\%$ agarose gel. RNA levels were quantified using Biorad Image Lab Software (see Supplementary Figures S5–S7, and Supplementary Table S5). Statistical significance between control and knock-out samples is assessed using a one-sided *t*-test.

### Availability of data and materials

We have implemented the StitchIt algorithm, the UnifiedPeaks approach, and a linking using previously defined regions (e.g. from GeneHancer) using C++. Each linkage method, except for the unsupervised peak linkage(www.github.com/schulzlab/TEPIC (60)), is available as a separate executable in the StitchIt repository: www.github.com/schulzlab/STITCHIT. The code can be easily build using cmake (version ≥3.1) and requires a C++11 compiler supporting *openmp* for parallel execution of StitchIt. We have thoroughly tested StitchIt using googletest. Raw data (Supplementary Table S9) can be downloaded from the ENCODE data portal for Roadmap data. To gain access to raw data files from Blueprint, a data access application needs to be submitted. Files generated within this study are available at Zenodo (https://zenodo.org/record/4077842). The repository includes not only all processed files, but also the predictions of REMs computed by StitchIt, the UnifiedPeaks, and the GeneHancer approach. The genome annotation file from GenCode (61) as well as the candidate REMs from the GeneHancer database are included in the StitchIt repository at www.github.com/schulzlab/STITCHIT.

Additionally, we provide a publicly available and user-friendly web server, called EpiRegio to query the predicted REMs of StitchIt. For the results presented on EpiRegio, StitchIt was applied to the Roadmap and Blueprint data, as mentioned before. To take even distant REMs into account, per gene a window of 100 000 bp upstream of a gene’s TSS, the entire gene body and 100 000 bp downstream of a gene’s TTS are considered. EpiRegio allows to search for REMs, which are associated to a set of genes or overlap with a given genomic region. The web server is available at https://epiregio.de/ (62).

**Supplementary Material**: Supplementary Section 1 contains details on data processing. Supplementary Section 2 holds a more detailed description of the Poe algorithm. In Section 3, details on the StitchIt algorithm are provided. Details on related methods to link regulatory elements to genes are shown in Section 4. Additional Figures and Tables are listed in Supplementary Section 5.

**Supplementary Excel Sheet**: The excel sheet contains Supplementary Table S7 with information on the intersection between STITCHIT and GWAS hits.

## RESULTS AND DISCUSSION

### A novel method for the gene-specific identification of regulatory sites

We present StitchIt, a novel segmentation based method to identify gene-specific REMs. Unlike other approaches (24,33), StitchIt solves the problem of defining regulatory elements and identifying their target genes in an integrative, joint approach and not in a sequential manner. It is a peak-calling free approach interpreting the epigenetic signal in relation to the expression of a distinct gene *g*. Basically, StitchIt solves a classification problem by segmenting open-chromatin signal in a large genomic area around the query gene *g*. The resulting segmentation highlights regions exhibiting epigenetic signal variance, which is linked to the expression of the analysed gene (Figure 1, Supplementary Figure S2). Thereby, StitchIt can be used to look at aspects of gene regulation in a gene-specific manner, and can therefore stimulate novel biological investigations. Here, we apply StitchIt to a collection of paired, uniformly reprocessed DNase1-seq and RNA-seq samples from Blueprint and Roadmap to determine gene-specific REMs. These datasets are very different, e.g. the Blueprint dataset is rather homogeneous representing a wide spectrum of the haematopoietic lineage and the Roadmap dataset is a large, highly heterogeneous dataset, see 1. Thus, these two datasets are ideal to test the capabilities of StitchIt, which we did in various validation and application scenarios.

StitchIt has two main parameters that influence performance and runtime: the *segment-size* and the *resolution*. We have tested several values for both parameters and have set the segment-size to 5000 and the resolution to 10 (Supplementary Figure S8) as these parameters yield a good trade-off between performance, assessed in terms of gene-expression prediction performance, and runtime. An additional parameter that is to be specified is the size of the considered genomic region up- and downstream of a gene. This parameter influences whether distal associations can be discovered and influences the runtime of the tool. We have conducted runtime experiments (Supplementary Figure S9) and found that even with a window size of 0.5MB (excluding the size of the genes) REMs can be learned in about 10 min per gene. As regulatory interactions typically arise within topological associated domains (63), this is also a feasible value in practice, especially for analyses focusing only on a few distinct genes. For all results presented here, we consider an extension of 25 kb upstream of a gene’s TSS and downstream of a gene’s TTS (see Methods), as we are focused on the comparison with other methods and on the illustration of the novelty of the approach.

### StitchIt leads to gene-specific regulatory regions derived from gene-expression prediction models

In order to understand, whether the integrative prediction approach of StitchIt outperforms previous methods, we did a number of comparisons. However, the comparison with previous sequential methods is not straightforward, as StitchIt defines REMs in a gene-specific manner. Thus the prediction of REM location and its target gene are coupled. In the two following sections we first investigate the regions from the perspective of the target gene, and then validate the interactions using external data.

We compared StitchIt to two sequential approaches using the same data sets. The first is denoted UnifiedPeaks, and resembles the standard approach that researchers would consider, defining REMs based on peak calls over many samples (see Materials and Methods). The second is a literature based approach using the GeneHancer database, which provides a list of candidate regulatory elements for each gene. For those approaches, we analyse the suggested REMs from a biological perspective, and also characterize the gene-expression prediction models and the inferred REMs from a technical perspective.

**Figure 2.**
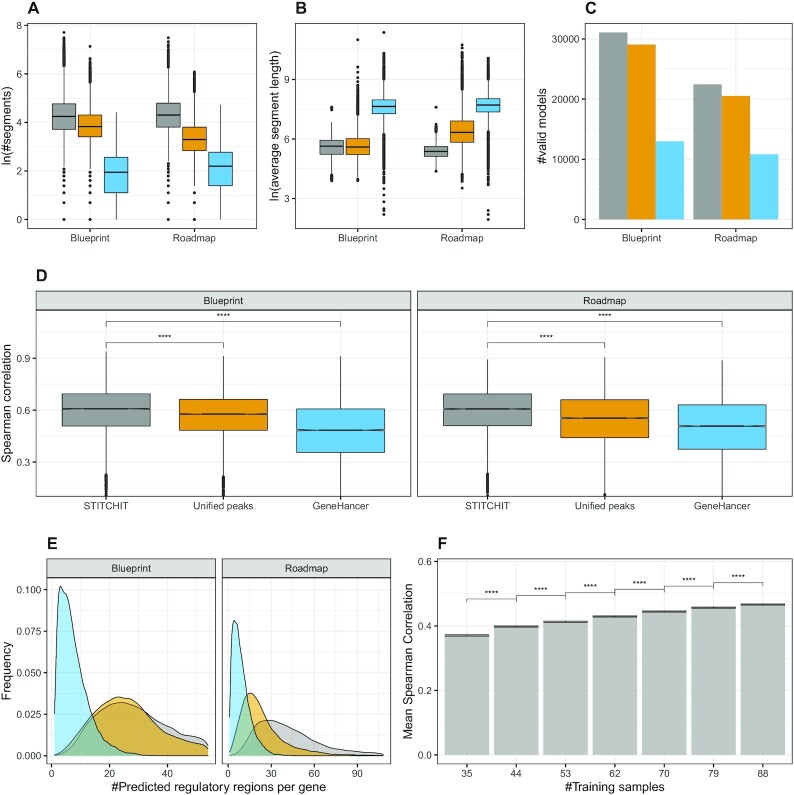
(**A**) The natural logarithm of the number of segments selected by StitchIt, UnifiedPeaks, and GeneHancer is shown for each dataset respectively, whereas in (**B**), the average length of the selected segments is depicted. (**C**) The number of learned models is shown, separately per consortia and method. (**D**) Boxplots showing Spearman correlation between predicted and measured gene-expression using linear regression with elastic net penalty considering all regions identified by StitchIt, the UnifiedPeaks approach, GeneHancer, and individual peak aggregation respectively for Blueprint and Roadmap data. Within StitchIt, UnifiedPeaks, and GeneHancer Spearman correlation was used for the initial filtering of candidate regions. Within each consortia, the same set of genes is displayed to allow comparability (Blueprint: 11140, Roadmap: 9102). As indicated by a two-sided *t*-test, StitchIt regions achieve the best model performance (****P ≤ 0.0001). The estimated values for the variances are: 0.018, 0.017, 0.029 (Blueprint), 0.018, 0.024, 0.032 (Roadmap), for StitchIt, UnifiedPeaks and GeneHancer, respectively. (**E**) The density plots delineates the number of predicted REMs per gene, shown separately for the used datasets and tested methods. Note that, due to the design of the linear model, the maximum number of predicted REMs is capped by the number of samples used for model training. (**F**) Considering $80\%$ of the entire Roadmap data set, we performed down-sampling experiments training 10 models for each gene with a different number of training samples, evaluated on the remaining $20\%$ of the data. According to a two-sided *t*-test (****P ≤ 0.0001), the performance drop is significant for each reduction of training samples.

As illustrated in Figure 2A, both StitchIt and UnifiedPeaks identify more candidate regions per gene than GeneHancer. In Supplementary Figure S10c, it is illustrated how many REMs are retained by the filtering steps performed in the regression pipeline. Simultaneously, the regions retrieved by StitchIt and UnifiedPeaks are shorter than those extracted from GeneHancer (Figure 2B). The same observation is made using Pearson correlation as a measure to filter candidate regions (Supplementary Figure S10b). This suggests that although StitchIt predicts more individual segments, the total genomic space covered by those must not be larger than that of UnifiedPeaks regions. As shown in Supplementary Table S6, the UnifiedPeaks regions indeed cover a larger fraction of the genome than StitchIt and GeneHancer regions.

Figure 2C depicts the number of genes for which a model could be learned per consortia and linkage method. StitchIt and UnifiedPeaks segments lead to more statistically significant models than GeneHancer segments. Also, StitchIt finds slightly more significant models than UnifiedPeaks.

In Figure 2D, the Spearman correlation of elastic net models predicting gene-expression from the DNase1-seq signal within the identified REMs is depicted (c.f. Supplementary Figure S11a for other measures). The correlation is computed using a 10-fold outer Monte Carlo cross-validation procedure (see Materials and Methods). To allow for comparability, we only show model performance for genes that are covered by each tested method. Additionally, we have performed benchmarking using a nested execution of StitchIt as explained in Figure S3B to check for inflated performance estimates due to over-fitting and/or the presence of all samples at both feature generation and feature selection steps (see *Overestimation of model performance in predicting gene-expression* for details). As illustrated in Supplementary Figure S10a, using Spearman correlation for the internal filtering leads to a better model performance and was thus used for all experiments in the manuscript.

In Supplementary Figure S12 we also show the performance for a baseline model that uses window based peak aggregation, labelled as *Individual peaks* (see Methods). There, we show for each gene only the best performing model based on either the 5 kb, 50 kb or the *geneBody* window. Across all datasets, we observe that models based on StitchIt regions achieve a significantly better correlation (*P* ≤ 0.0001) than models based on any other approach. This is independent from the correlation measure used for the initial filtering of REMs within StitchIt, UnifiedPeaks, and GeneHancer. In a gene-to-gene comparison (Supplementary Figure S11b, c) StitchIt shows favourable performance, too.

An important difference between GeneHancer compared to both StitchIt and UnifiedPeaks is that the GeneHancer models are relying on a curated database of known regulatory elements. We assessed how many enhancers contained in the entire database of GeneHancer are retrieved using the OLS model. In general, only very few elements are selected (Supplementary Figure S13). For instance, if the database contains 6 enhancers for a gene, on average 3 are chosen by our models. For genes with more enhancers, e.g. 50, about $25\%$ are considered by the model. These differences may be due to the missing tissue specificity of the GeneHancer entries. Further, our results indicate that the supervised generation of REMs as performed in StitchIt outperforms the unsupervised selection considerably, as different window sizes used with the unsupervised approach can not generalize well across different genes (Supplementary Figure S12).

We assessed whether model performance depends on genomic features, such as gene length or the number of isoforms. As shown in Supplementary Figure S14 models for longer genes tend to perform better than those for shorter genes. Consequently, also genes with more than one isoform tend to perform better. In addition, we observe that both mean and standard deviation of gene-expression is linked to model performance: models for genes with both high mean expression and variation perform better than those for only marginally expressed genes.

In Supplementary Figure S15, we sketch the distribution of StitchIt regions around a gene. As expected and supported by chromatin conformation data (ChIA-PET and Promoter Capture Hi-C), we see enrichment at the TSS for all tested methods and a depletion up- and downstream of the TSS. Notably GeneHancer has the highest enrichment at the TSS, which might be due to the strong reliance of GeneHancer on regulatory interactions reported in literature.

The density plots of Figure 2E illustrate the distribution of the total number of REMs predicted per target gene. Our results indicate that StitchIt tends to find more sites per gene than the UnifiedPeaks approach. Furthermore, the distribution for GeneHancer is different compared to that of UnifiedPeaks and StitchIt. While the latter two reach the maximum, depending on the dataset, between 20 and 30 REMs per gene, GeneHancer reaches the optimum at 1–4 predicted sites per gene. Note that due to the architecture of the OLS model, the maximum number of REMs called is capped by the number of samples available in each data set.

To get a better understanding of how many samples are needed to run StitchIt, we performed down-sampling experiments on the Roadmap dataset. Briefly, we considered $80\%$ of the Roadmap data for training and the remaining $20\%$ for testing. From the training set, we generate down-sampled subsets with a step size of $10\%$ starting at $40\%$ of the data (see Methods). As shown in Figure 2F, reducing the number of training data does lead to a significant drop in model performance. Although models could still be fitted with as few as 35 samples, we recommend to use as many samples as possible to avoid over-fitting and to ensure that models can be generalised.

To further investigate the co-regulation of genes by various enhancers, we checked for the occurrence of *additive* enhancers, a term postulated by Anderson *et al.* (52), among all REMs identified with StitchIt. Anderson *et al.* define enhancers as *additive* if they have a strong regulatory contribution and are correlated to other enhancers regulating the same gene. Similar to their finding, we see a trend that genes with many additive enhancers tend to be higher expressed than others (Supplementary Figure S16a). However, only few additive enhancers exist in our data set (Supplementary Figure S16b).

Overall, we observed that especially on large heterogeneous datasets, such as the Roadmap dataset, the peak-independent generation of REMs shows clear advantages over the peak-based strategies. While the Blueprint dataset is composed of primary cells related to the hematopoietic lineage, the Roadmap dataset is more diverse and also comprised of tissue samples. On the more homogeneous Blueprint data, StitchIt and UnifiedPeaks identify almost the same number of segments with similar length. In contrast to that, on Roadmap data, StitchIt selects more, but shorter REMs than UnifiedPeaks (Figure 2A, B). This difference is also reflected by the performance of the gene-expression models (Figure 2D). The most likely explanation for this behavior is that due to the high variance in the Roadmap data, merging peaks introduces a loss of specificity, by removing the information of the exact genomic location of accessible chromatin (Supplementary Figure S1). StitchIt is more suited to resolve the sample and tissue specific variance, therefore obtaining better results on Roadmap data compared to the UnifiedPeaks method.

### Validation of REMs and of regulatory interactions using external data

**Figure 3.**
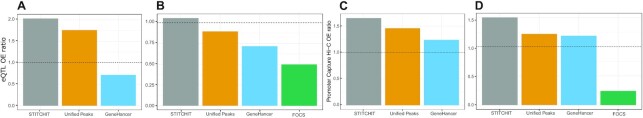
Comparison with eQTL and chromatin conformation capture experiments. Observed over expected (OE) ratios for the number of unique REMs correctly overlapping annotated regions are obtained in comparison to a background dataset for each method, which is matched in REM sizes, lengths and distances to the TSS of genes. OE ratios using GTEx eQTL data (48) are shown for (**A**) Blueprint and (**B**) Roadmap predictions. OE ratios for the number of unique REMs correctly overlapping with Promoter Capture Hi-C data (51) are shown for (**C**) Blueprint and (**D**) Roadmap data.

Expression quantitative trait loci (eQTLs) are distinct genomic loci that are linked to the expression of genes. We obtained eQTL data from the EMBL-eQTL catalog (48) and overlayed it with our predictions by computing how many unique REMs are correctly overlapping with eQTLs (Supplementary Figure S17A, B). As each tested method identified different number of REMs, we generated specific background datasets matching size, length and distance to the TSS of genes and compared that with the real REM collections (Supplementary Figure S17A, B). In Figure 3A, B, we show the Observed over Expected (OE) ratios for eQTL overlaps for Blueprint and Roadmap, respectively (see Materials and Methods). We find that StitchIt achieves the highest OE ratio in terms of overlap with eQTLs compared to any other method. In fact, on Roadmap data, StitchIt is the only method achieving an OE ratio >1. The larger OE ratio strongly suggests not only that StitchIt REMs link to the correct target gene, but also that StitchIt is able to detect more accurate regulatory regions than the competitors.

Another approach to show the reliability of our predictions is to assess the amount of rediscovered interactions from the GeneHancer database. In total, $32\%$ and $36\%$ of GeneHancer interactions are retrieved for Blueprint and Roadmap using StitchIt, respectively. UnifiedPeaks retrieves less than that, i.e. $30\%$ and $34\%$, respectively. While those numbers might seem low in general, it is important to remember that GeneHancer is based on many more (epi)genomic data sets and data types than any of the other methods tested here.

Chromatin conformation capture technologies such as Hi-C have demonstrated the prevalence of long-range regulatory interactions throughout the genome (64).

We compared REMs against several chromatin conformation data sets including Promoter Hi-C Capture data generated in scope of the Blueprint project (51). On this high quality data set, StitchIt achieves the best OE ratio using both the matching Blueprint REMs as well Roadmap REMs (Figure 3C, D, Supplementary Figure S18). In addition to the chromatin conformation data generated on primary cells, we compared the learned interactions to ChIA-PET data for K562 and MCF-7 cells (targeting RNA polymerase II) as well as to Promoter-Capture Hi-C data for GM12878 cells (Supplementary Figure S19A, B). As above, we contrast the number of unique REMs overlapping with experimentally confirmed chromatin interaction to the overlap achieved with random REM sets (Supplementary Figure S19C,D). While the UnifiedPeaks approach performs better than StitchIt and GeneHancer on Blueprint data, StitchIt considerably outperforms the other methods on Roadmap data. Notably, similar to the eQTL analysis, StitchIt is the only method achieving an OE ratio >1.0 when compared to ChIA-PET data using Roadmap REMs.

In an effort to better characterize REMs that are supported by conformation capture data we investigated the distance of REMs to their genes TSS and their absolute regression coefficients. For this analysis, we considered only REMs with a non-zero DNase1 signal in K562, MCF7 or GM12878 cells, matching the cell-lines used for the chromatin conformation capture experiments. In case of StitchIt, ChIA-PET supported interactions have on average larger OLS regression coefficients compared to unsupported interactions in three out of four comparisons, whereas with GeneHancer and UnifiedPeaks this holds for two out of four comparisons. With respect to the distance of REMs to the TSS of their target genes, we find that REMs supported by ChIA-PET data tend to be closer to the TSS than unsupported REMs (Supplementary Figure S20). A similar trend can be observed in eQTL data: supported REMs are closer to the TSS and their OLS coefficients tend to be higher compared to unsupported REMs (excluding StitchIt coefficients for Blueprint data) (Supplementary Figure S21).

For Promoter Capture Hi-C data from GM12878 however, we observe that supported StitchIt REMs tend to be further away from the TSS than unsupported REMs (Supplementary Figure S22A), while there is no significant difference for GeneHancer and UnifiedPeaks. While this is contradicting the ChiA-PET results it might be explainable by the differences in experimental design of two assays. While ChiA-PET contacts are enriched for regions that are in close contact to the RNA-PolII, Promoter capture Hi-C performs this enrichment using promoter containing restriction fragments, hence the obtained interactions will follow a different distribution. We did not find any significant difference with respect to regression coefficients for supported REMs and unsupported REMs in context of Promoter Capture Hi-C data from GM12878, across all tested methods (Supplementary Figure S22B).

We note that the results obtained for GeneHancer REMs in the validation experiments are biased as eQTLs and other data sources have been used in the generation of the GeneHancer database itself and therefore need to be taken with a grain of salt.

### Comparison against REMs identified by FOCS

In addition to comparing StitchIt to GeneHancer and the UnifiedPeaks approach, we performed all validation experiments mentioned before contrasting StitchIt against one of the current state of the art methods to predict promoter-enhancer-interactions (PEIs), Focs (24). Focs uses a regression approach to select the most relevant REMs for a gene out of a candidate list comprising 10 REMs. The gene-specific candidate lists are compiled using a nearest-neighbour approach on external data, e.g. DNase1-hypersensitive sites from Roadmap. We obtained Focs predictions for Roadmap data from the Focs website and considered those predictions for a comparison to StitchIt. Note that Focs predictions are not available for Blueprint data.

As shown in (Figure 3B, D), StitchIt performs favourably compared to Focs. StitchIt REMs show a higher OE ratio with eQTLs than Focs (Figure 3B) and also shows a higher OE ratio with ChIA-PET and Promoter Capture Hi-C elements (Figure 3D). These results demonstrate the general limitations of sequential approaches compared to StitchIt. Due to the initial selection of only 10 predefined REMs per gene, Focs is very limited in elucidating more complex regulatory mechanisms.

### Experimental validation of enhancers suggested by STITCHIT using CRISPR-Cas9 experiments in HUVEC

To further test the reliability of StitchIt, especially with respect to the validity of our predictions in unseen tissues, we performed a CRISPR-Cas9 experiment (three replicates each) targeting three different StitchIt REMs identified for KLF2, NOS3 and AC020916 in HUVECs. Note that this cell type was not used for learning. Further experimental details are provided in the methods section.

**Figure 4.**
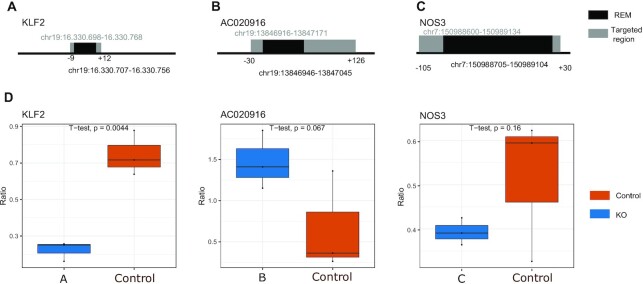
CRISPR-Cas9 based validation of REMS in HUVECs. (**A–C**) Genomic position of the considered REMs (shown in black) and the area targeted by the gRNAs (shown in grey). These REMs are predicted to be involved in the regulation of the genes KLF2 (**A**), AC020916 (**B**) and NOS3 (**C**). (**D**) Comparison between RNA levels for control and CRISPR-Cas9 knock-outs in HUVEC clones (3 replicates). Statistical significance was assessed with a one-sided t-test.

We chose REMs to be tested based on the expression of their target genes in HUVECs, the accessibility of the REMs in HUVECs and the ability to generate appropriate gRNAs. Selecting REMs to be experimentally validated based on both the regression coefficient as well as the activity of REMs is also motivated by the observation that accessible StitchIt REMs supported by ChIA-PET data have a higher regression coefficient than unsupported REMs (Supplementary Figure S20). Figure 4A–C show the genomic location of the considered REMs and the region targeted in the CRISPR experiment. Although all three REMs are included in the GeneHancer database, none of them have been identified using Focs on the Roadmap data sets used above. The tested REMs for NOS3 and AC020916 overlap with a H3K27ac peak in HUVECs. Also, we note that the REM tested for NOS3 has not been detected using the UnifiedPeaks approach.

After quantification of expression (see Supplementary Figures S5–S7) we observe a trend of changed expression patterns in all three genes compared to the controls suggesting a true biological role for the tested REMs. Two of three genes showed significant differential expression after excision of the REM (Figure 4D) (*P*-value ≤ 0.1 with a one-sided *t*-test).

The ability to reliably transfer and apply StitchIt predictions to an unseen cell type indicates the robustness of StitchIt and give rise to numerous interesting applications, where the StitchIt model can be used guide experimental design. Therefore, we decided to leverage the unique features of StitchIt and have additionally generated regulatory maps with a 100 000 bp extension up and downstream of all human genes using StitchIt. We have included those extended regulatory maps in the Zenodo archive as well as in the EpiRegio webserver. To take even distant REMs into account, EpiRegio contains REMS based on a window of 100 000 bp upstream of a gene’s TSS, the entire gene body and 100 000 bp downstream of a gene’s TTS. The webserver is available at www.epiregio.de. (62). Additionally, the above findings strongly suggest that models considering only a narrow area around a gene for REM detection are not sufficient.

### Partitioning of large regulatory elements using STITCHIT

**Figure 5.**
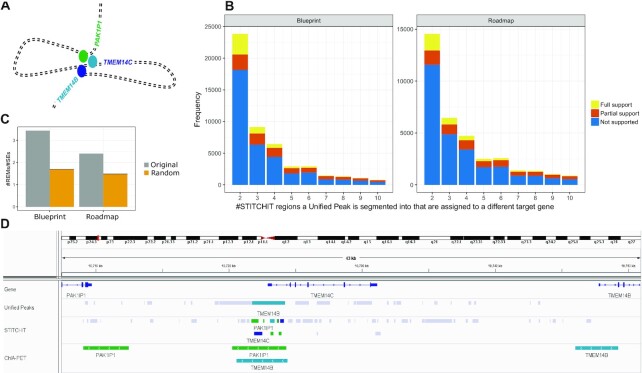
(**A**) Schematic illustration of chromatin folding: Three genes, namely TMEM14B, TMEM14C and PAK1P1 are brought to close spatial proximity via loop formation thereby establishing regulatory interactions between the genes and their enhancer elements (colored bubbles) that together form a cluster of enhancers that is in close genomic proximity to TMEM14C. (**B**) The bar plots indicate on the x-axis the magnitude of a *Split event*, that is the number of differently linked StitchIt segments a peak is split into. The y-axis holds the frequency for the individual counts. The color code indicates whether StitchIt associations are fully supported by conformation data, partially supported or not supported at all. (**C**) The overlap of STITCHIT REMs to the SEdb [18], a database for superenhancers, is shown for all STITCHIT REMs that split a Unified-Peak (grey) and for randomly picked genomic regions. The actual REMs have a higher ratio score, indicating that there are more REMs per superenhancer compared to the random data. (**D**) Example for a *split event* at the *TMEM14C* locus. At the promoter of *TMEM14C*, a peak that is linked to *TMEM14B* is split into several StitchIt segments. These are associated to PAK1P1, *TMEM14C* itself, and *TMEM14B*. All StitchIt associations shown here are supported by ChIA-PET data.

As shown above, the UnifiedPeaks approach produces longer candidate regions than StitchIt. Those larger elements are likely to be clusters of many individual REMs. *In-vivo* such regulatory clusters could arise for instance by chromatin looping, as illustrated in Figure 5 A. Here, we define a *split event* as the occurrence of a peak, detected by UnifiedPeaks, which is divided into several REMs by StitchIt with the additional constraint that the new sub-REMs should not be linked to the same gene as the original peak. As depicted in Figure 5B such *split events* do occur frequently. Note, that for illustration purposes, split events of degree >10 are not displayed in Figure 5B. The color code indicates whether the splits are supported by ChIA-PET data in K562 cells. If and only if all StitchIt regions are supported, we call a split *fully supported*, if not all but at least one region is supported we call it *partially supported* (see Materials and Methods). In addition to the absolute numbers of *fully* and *partially* supported split events shown in Figure 5B (Supplementary Figure S23 provides the full support rate for all *split events*). While the full support rate is high for split events with degree 2, (about $13\%$ and $11\%$, for Blueprint and Roadmap data, respectively), it gradually drops with increasing split event degree to ∼$5\%$ across both data sets. We note that most StitchIt REMs overlap with a peak contained in the candidate set considered by the UnifiedPeaks method, however most of those peaks are either removed by the correlation filter or the regression step (Supplementary Figure S24B).

To ensure that splitting of peaks into REMs that are assigned to the same gene as the original peak is not an artefact of StitchIt caused by over-fitting, we examined the median length of splitted peaks for various split event degrees. As indicated in Supplementary Figure S24a, the length of the splitted peaks increases constantly with an increasing split event degree, suggesting that indeed only peaks covering large genomic intervals are subject to splitting.

The observation that regions, which are subject to splitting cover large genomic regions (Supplementary Figure S24A), lead us to the hypothesis that these are regions of high regulatory activity. For example, superenhancers are clusters of enhancers covering a vast genomic space (65). We computed the overlap of REMs involved in split events with a curated database of superenhancers, known as SEdb (55). Compared to background models, which adjust for the total number of REMs (see Methods), we find that REMs that are part of split events are enriched in superenhancers across both data sets (Figure 5C).

An example for a split event in our data is provided in Figure 5D. To simplify the example, we are using the same genes used in the 3D illustration of Figure 5A. Here, a peak is linked exclusively to *TMEM14B* by the UnifiedPeaks method. The peak itself is located around the promoter of *TMEM14C* and covers a total genomic range of 2497*bp*. StitchIt divides that peak into segments linked to PAK1P1, to *TMEM14C* itself, and to *TMEM14B*. ChIA-PET data obtained from K562 cells supports the long range interactions to *PAK1P1* and *TMEM14B*. This example, together with the analysis presented in Figure 5B underlines the ability of StitchIt to precisely pinpoint regions of regulatory potential and suggests the application of segmenting large REMs, into more refined segments to reveal their regulatory interactions.

### Exploratory analysis of the regulatory landscape of *EGR1*

**Figure 6.**
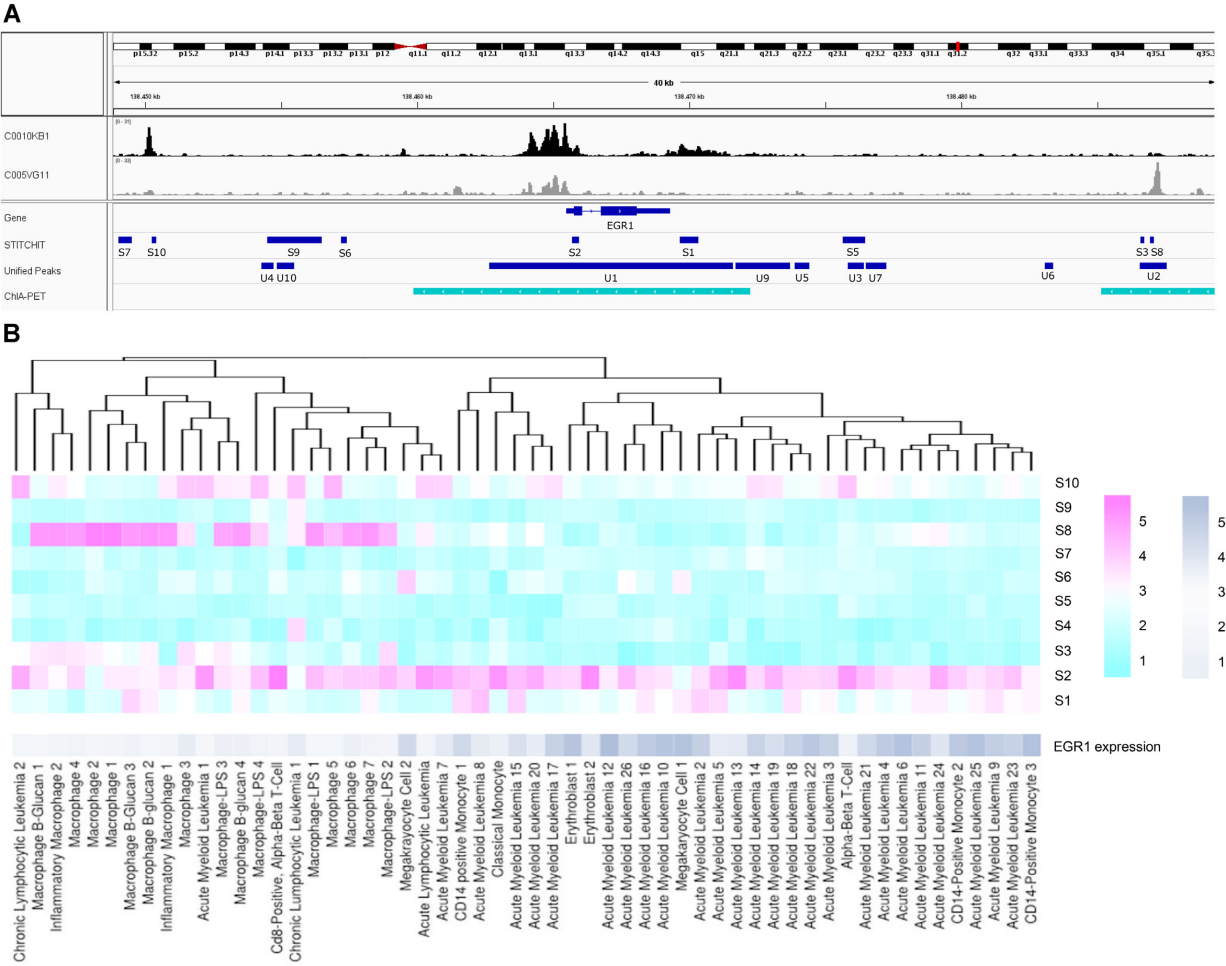
(**A**) Genome browser tracks describing the regulation of *EGR1*. Track *C0010KB1 (black)* exemplifies the DNase1-seq signal for a sample where *EGR1* is expressed, whereas track *C005VG11 (gray)* illustrates the case where *EGR1* is not expressed. (**B**) Heat map that is clustered according to the DNase1-seq signal in the candidate REMs *S*1...*S*10 identified by StitchIt, the gene-expression of *EGR1* is not used for the clustering itself and shown for illustration purposes only. The data has been log transformed with a pseudo-count of 1. Two major clusters can be observed corresponding to samples where *EGR1* is expressed and to those samples where *EGR1* is not expressed. The heatmap shows the *log*_2_ of read counts for DNase1-seq, and log_2_ of TPM for gene-expression, respectively.

To better understand the functional advantage of StitchIt over UnifiedPeaks, we have investigated the regulatory landscape of *EGR1* in more detail. For *EGR1*, the Spearman correlation achieved by the UnifiedPeaks REMs in gene-expression modelling is 0.55, while StitchIt regions achieve a correlation of 0.72. Here, we test whether this difference in model performance is also reflected by an improved interpretability of the identified regions regarding the regulation of *EGR1*. In Figure 6 A, we show the identified candidate regions ranked according to the absolute value of the regression coefficients per site (Supplementary Table S8). A striking difference between StitchIt and UnifiedPeaks is that the latter identifies one large segment (U1: 8970bp) covering 2842bp upstream of *EGR1*, the entire *EGR1* gene as well as 2304 bp downstream of *EGR1* TTS. This segment is split up into two regions using StitchIt: a region downstream of *EGR1* TTS (S1), and into a region within the first exon of *EGR1* (S2). As shown by the DNase1-seq signal tracks in Figure 6A, StitchIt region S1 and S2 do overlap DNase1-seq signal in sample *C0010KB*, in which *EGR1* is expressed, whereas they lack signal in *C005VG11*, where *EGR1* is not expressed. It is likely that this difference between StitchIt and UnifiedPeaks is the main reason for the observed performance difference.

Another interesting association can be observed for S3 and S8, which also overlap a segment identified with UnifiedPeaks (U2). S3 has the strongest negative regression coefficient identified by StitchIt for *EGR1* and indeed this region (as well as S8) shows signal in *C005VG11* but not in *C0010KB*, supporting the role of the regions as an active repressor of *EGR1*. The link of S3 to *EGR1* is further supported by ChIA-PET data.

While these examples provide insights on the level of individual samples, we have considered the DNase1-seq signal within all identified StitchIt regions and used it to cluster the Blueprint samples (Figure 6B). Using only the signal within the candidate regulatory sites, an almost perfect clustering into samples according to *EGR1* expression levels could be obtained. The clustering can be used to assess the cell-type specificity of the suggested regions.

### STITCHIT allows a characterization of repressive elements

StitchIt enables not only the gene-specific identification of REMs, it also allows to characterize the effect of REMs on the expression profile of the target genes. We used this feature to investigate whether there is a difference between the location of elements with a positive and those with a negative association around their target gene. As Supplementary Figure S25 illustrates, we do observe differences. Compared to background models generated by randomly shuffling regression coefficients we found that REMs being positively associated to gene-expression are enriched at a 5 kb bin located at the promoter of genes, the gene body as well as directly downstream of their target genes. However, they are depleted further up and downstream. REMs with a negative regression coefficient on the other hand tend to behave as predicted by the random model with the exception that they are also enriched at the promoter and that they tend to be depleted downstream of genes.

Till this point of our analysis, we have used the catalog of REMs computed by StitchIt in a gene-specific manner, i.e. all scores and validation criteria have been performed from the perspective of genes. However, an obvious question to ask is whether there are REMs that are shared between genes and how the association of those REMs to their target genes can be characterized. To answer this question, we generated a union set of REM for each considered data set (Blueprint and Roadmap) using the BEDTools*merge* command (66). We refer to the resulting elements as *CREMs*. Note that if a REM does not overlap any other REM, the original REM is identical to the corresponding CREM. Specifically, this lead to 535 579 CREMs for Blueprint and 704 735 CREMs for Roadmap data. By intersecting the CREMs with the original, gene-specific REMs using BEDTools*intersect*, we identified CREMs that are linked to either one or to more than one gene. As depicted in Supplementary Figure S26 most CREMs are uniquely associated to one gene only, a small fraction of CREMs is linked to multiple genes ($13\%$ Blueprint, $11\%$ Roadmap datasets).

**Figure 7.**
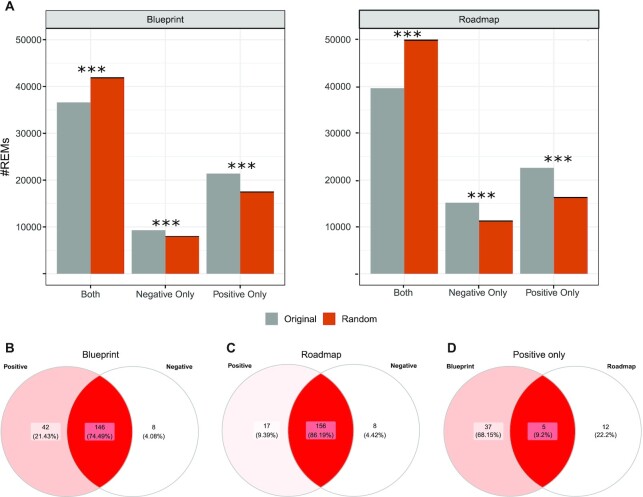
(**A**) Number of StitchIt REMs that are associated to multiple different genes in either a positive, negative, or in both ways compared against a random background model using randomly shuffled coefficient distributions. Venn Diagrams depicting the overlap of TFs found enriched in positively and negatively associated REMs for (**B**) Blueprint, and (**C**) Roadmap data. (**D**) Overlap among all TF sets enriched in REMs with positive association across the considered data sets.

We find that the majority of those CREMs are associated to both positive and negative regulatory effects. As one might expect, we also find that there are more unique CREMs with a positive than a negative association (Figure 7A). These trends are invariant to both the number of genes a CREMs is targeting and to the considered regression coefficient cut-off (Supplementary Figure S27). Compared to background models that randomize the assignment of regression coefficients to CREMs, the described observations occur more often than expected by chance (Figure 7 A, Supplementary Figure S28).

We further characterized the exclusively positive and negative CREMs in terms of the TF binding sites they contain. Using TRAP (54), a method that predicts TF binding using a biophysical model, we obtained lists of enriched TF motifs and investigated the overlap between TFs enriched in positively and negatively associated CREMs for Blueprint (Figure 7B), and Roadmap (Figure 7C) (Supplementary Table S9). We found that most motifs are shared although some TFs occur exclusively in CREMs with a positive sign and some occur exclusively in CREMs that are assigned a negative regression coefficient.

For instance, YY1 and YY2 occur exclusively in positive CREMs in Blueprint data. This is a sensible prediction as YY1 is known to act as an enhancer (67). Another illustrative example is that the known repressor FOSL1 is enriched in repressive elements of Roadmap data (68). As shown in Figure 7D, only five TFs are commonly enriched in positive REMs among all data sets, including RUNX2 and RUNX3. Both are known key regulators and have been reported to control osteoblast differentiation, cell cycle state and CD8+ T cell development, respectively (69). The low overlap between different datasets suggests that the detection of TF motifs may be influenced by the tissue- and cell type specific regulatory landscape investigated by the different consortia.

### STITCHIT learns more putative regulatory regions than other approaches

**Figure 8.**
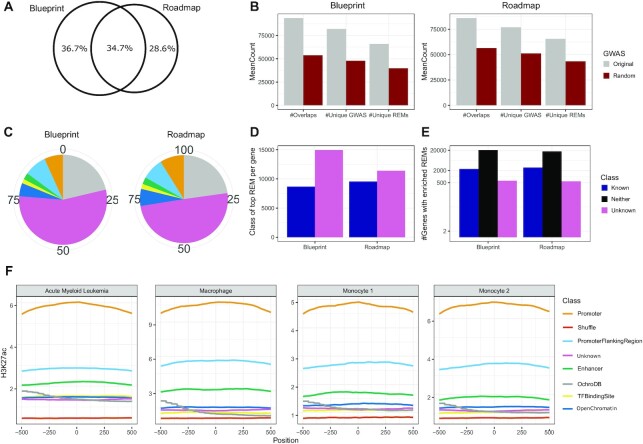
(**A**) The Venn diagram illustrates the overlap of genes for which a model could be learned using StitchIt on either one of the datasets used. (**B**) Number of overlaps between StitchIt REMs and GWAS sites, number of unique GWAS SNPs overlapping with StitchIt REMs and number of unique StitchIt REMs overlapping a GWAS site using the actual GWAS catalog (grey) and 100 randomly sampled SNP sets (red) . (**C**) The distribution of a mapping of StitchIt sites to the ERB and OCHROdb is shown for Blueprint and Roadmap samples. (**D**) Number of genes where the StitchIt REM with the highest regression coefficient is either *known* or *unknown*. (**E**) Number of genes where either *known*, or *unknown*StitchIt REMs are enriched sorted according to the top absolute regression coefficients. (**F**) H3K27ac signal shown for randomly shuffled regions, as well as StitchIt regions split according to the categories obtained from intersecting all StitchIt segments with the ERB and OCHROdb. H3K27ac signal is shown for four Blueprint samples in a window of 1kb centered in the middle of the putative REMs. StitchIt regions overlapping *Promoter* or *Promoter Flanking Regions* show the highest H3K27ac signal, while the signal in randomly determined regions is the lowest. The largest portion of regions, labeled as *unknown* have a similar signal intensity as sites labeled as a *TF binding site* or *Open Chromatin*.

We have seen earlier that StitchIt tends to find more REMs per gene than both UnifiedPeaks or GeneHancer (Figure 2A, Supplementary Figure S10A). In addition to that, we also observe that the overlap in terms of genes for which a model could be learned, is less than $50\%$ between two datasets (Supplementary Figure S29A–C), independent from the method used for the computation. Specifically for StitchIt, only $34.7\%$ (4477) of all gene-specific models are shared between Blueprint and Roadmap. Just $36.7\%$ (8214) of all genes could be exclusively modeled using Blueprint data and $28.6\%$ (6917) with Roadmap (Figure 8A). As shown in Supplementary Figure S29D–F, genes that could be exclusively modeled in Blueprint data tend to be higher expressed in Blueprint than in Roadmap data and vice versa. Analogously, genes that can be modelled in both data sets using StitchIt or UnifiedPeaks are equally expressed in Blueprint and Roadmap data.

Although we have shown that in gene-expression prediction experiments, StitchIt regions achieve a better agreement between predicted and measured gene-expression than related approaches (Figure 2D, Supplementary Figure S11), the performance of predictive models alone does not proof that the identified regions truly play a role in gene regulation. We stress that StitchIt associations do not imply causation. Thus, we can not distinguish whether the accessibility of certain regions is driving expression of a gene, or whether it is a consequence of that gene being expressed. Also indirect associations, which could be caused by co-regulation of genes, can not be avoided. These problems have to be addressed by other methods and will require substantial amounts of additional data to be solved. Therefore, it is especially important to characterize the predicted REMs, especially the uniquely detected ones, further.

An initial check for the functional relevance of StitchIt REMs is whether they exhibit overlaps with known GWAS sites retrieved from the EMBL-EBI GWAS catalog (see methods). Overall we find 93,707 associations with Blueprint and 86 066 with Roadmap data covering 82 041 and 77 006 SNPs respectively using StitchIt. Compared to a random setting considering 100 randomly sampled SNP sets with matchef MAF, all true regions yield significantly more associations (Figure 8B). The complete overlap results of the EMBL-EBI GWAS catalog with our REM predictions is also a unique and valuable resource allowing extensive downstream analysis as it suggests target genes for many GWAS sites (Supplementary Table S7).

Furthermore, we overlapped REMs with the Ensembl Regulatory Build (ERB) (43) and the OCHROdb database (44). In about a quarter of all cases, an overlap is found with a state annotated as Promoter, Promoter flanking region, TF binding site, Enhancer, or Open Chromatin from the ERB. One more quarter overlaps with the OCHROdb. However, roughly half of the StitchIt REMs do not overlap an annotated region (Figure 8C), thus they are labelled as *unknown*, whereas the remaining elements are labeled as *known*.

The question arises whether the *unknown* REMs are simply noise or whether they reflect REMs that have not been annotated so far. To investigate whether these *unknown* REMs are performing regulatory functions, we determined, for each gene, whether the REM with the highest absolute regression coefficient is labelled as *unknown* or *known*. As depicted in Figure 8D, *unknown* REMs are assigned to the highest regression coefficient for the majority of genes. In addition, we find that *unknown* REMs are enriched among the top REMs, sorted by absolute regression coefficient, for about 500 genes, while about 20 000 genes do not show enrichment for either *known* or *unknown* REMs (Figure 8E). These results suggest that *unknown* REMs are of high importance in the regression models, which does suggest a regulatory role for those.

To follow up on the hypothesis that *unknown* REMs are biological relevant, we assessed the signal of three histone marks (H3K27ac, H3K4me1 and H3K4me3) using ChIP-seq data sets for four randomly chosen Blueprint samples. We considered (i) the top 10,000 StitchIt REMs ranked according to their OLS *P*-value (cf. Methods), (ii) 10 000 randomly selected StitchIt REMs omitting their regression coefficient and *P*-value, (iii) a background set composed of 10 000 randomly picked genomic regions following the same size distribution as the original REM set and (iv) the top 10 000 regions per ERB-group. As indicated in Figure 8F the strongest H3K27ac signal occurs within *Promoter* and *Promoter Flanking Regions*. Importantly, the signal of the *Random* regions is the lowest. The signal of the *unknown* regions is similar to that of *TF binding sites* and *Open Chromatin* suggesting that these regions do have a regulatory effect. The association of StitchIt REMs to both active enhancers (H3K27ac) and promoters (H3K4me1/3) is further backed up by the observation that the HM signal in 10 000 randomly selected StitchIt REMs behaves similar to the signal of the top 10 000 REMs (Supplementary Figure S30). Furthermore, the DNase1-signal in *unknown* elements is relatively low but significantly higher than of *shuffled*, randomly picked genomic regions (Supplementary Figure S31).

Together with our previously described *in vivo* and *in silico* validation experiments, these results suggest that StitchIt is able to detect unknown but potentially biologically relevant REMs that can not be detected using currently available sequential REM detection methods such as Focs.

### Overestimation of model performance in predicting gene-expression

Estimating the performance of gene-expression prediction models (Figure 2D) is generally the first step in ensuring that REMs predicted by a model are worthy to be explored further, e.g. in validation experiments. As pointed out by a reviewer during the revision of this manuscript, the definition of candidate REMs is normally done on the complete dataset. For example, the Focs method used a set of REMs defined by members of the Roadmap consortium. The default StitchIt pipeline uses all available samples to generate a set of candidate REMs, which are then filtered using a correlation filter and used for elastic net regression. This leads to a circularity in testing model performance during elastic net regression as the test data considered in the cross-validation process has been previously used to define the candidate REM set, although not as part of a regression approach. To ensure that this problem does not lead to a vast overestimation of model performance as presented in (Figure 2D), we devised a nested execution of StitchIt (Supplementary Figure S3B), in which we subset $20\%$ of the entire data as test data before executing the StitchIt algorithm. This comes with the downside of loosing samples in generating the candidate set of REMs. As shown in Supplementary Figure S32 there is a slight drop in model performance. However, this drop is mostly due to the loss of training samples as we saw in our down-sampling experiments of the Roadmap data (Figure 2F). Given the amount of samples we used the prediction of relevant candidate REMs is hard, as the datasets contain many different cell types and/or tissues. REM locations that are more cell type-specific are difficult to obtain and thus we see a linear drop in prediction performance with samples used. Thus, we think that our MDL formulation prevents otherwise larger effects.

However, the circularity of feature generation and model evaluation is a potential problem of all methods considered in this article: UnifiedPeaks, GeneHancer and Focs. We attempted to also generate a nested version of the UnifiedPeaks approach, but were not successful due to the high computational costs of intersecting bed files for each gene as part of the cross-validation. For GeneHancer and Focs, it is not possible to avoid this issue in the first place, as some of the data used to build and evaluate the linear models, has been used initially to build the REM maps provided in GeneHancer and Focs. As can be expected, the nested mode of StitchIt is computationally more expensive than the default mode, but is available in our repository. With rising amounts of epigenetic data becoming available, it should be considered to generate a robust readout of model performance.

We believe that this problem also highlights again the importance of free data access and absolute transparency about which data types, samples and resources were utilized to generate any kind of publicly available REM database. Only then, potential issues of over-fitting in the models and circularity of overlap with other datasets can be detected and recognized.

## CONCLUSIONS

Our novel method StitchIt solves the combined task of identifying potential REMs, and linking them to their putative target genes at the same time. This is achieved by combining epigenetics and gene-expression data to identify a set of potential REMs considering the signal of the epigenetics data at hand, instead of pre-selected sites of enrichment. Hence, the peak calling step can be omitted. Subsequently, StitchIt regions are refined using a regression learning approach and a confidence score for each REM is computed. Our modeling approach allows a distinct REM to influence multiple genes. As StitchIt is based on the Minimum Description Length principle (MDL), over-fitting is naturally avoided as MDL balances the complexity of the description of the model and the complexity of the data given the model. In this work, uniformly processed DNase1-seq and RNA-seq data from IHEC is used, however our method is conceptually not limited to DNase1-seq data as a carrier of epigenetic information, but also works with ATAC-seq, FAIRE-seq or ChIP-seq data.

We have compared StitchIt against related strategies that are based on the integration of peaks, such as Focs (24), or on known REMs, as stored in the GeneHancer database (70), and show that StitchIt is not only able to learn more sites with regulatory potential than the other methods, while achieving a superior explanatory power of gene-expression, but StitchIt also performed well in various validation experiments including our own CRISPR-Cas9 validation experiments for three StitchIt REMs. Importantly, these experiments were carried out in a cell type that was not used for model training, suggesting the ability of our algorithm to generalise across tissues and cell-types. With the application of StitchIt to larger datasets in the future, including uniformly reprocessed IHEC data, a promising option for further validation could be the usage of massively parallel reporter assays.

Furthermore, we illustrate how StitchIt can be used in an exploratory manner to elucidate the regulation of a distinct gene exemplary for *EGR1*. StitchIt is efficiently implemented in C++ and freely available on github: www.github.com/schulzlab/STITCHIT. We believe that StitchIt paves the way for a seamless integration of the wealth of epigenetics data being produced and allows an easy-to-use analysis of transcriptional regulation on the gene-level.

## DATA AVAILABILITY

StitchIt is available on github: www.github.com/schulzlab/STITCHIT. All processed files and REMs generated in this manuscript are available at Zenodo (https://zenodo.org/record/4077842) as well as in the EpiRego webserver (https://epiregio.de).

## Supplementary Material

gkab798_Supplemental_Files
