# Supplementary material for: Integrative analysis of epigenetics data identifies gene-specific regulatory elements
Source: Nucleic Acids Res. 2021 Sep 11;49(18):10397–418. doi: 10.1093/nar/gkab798 (PMC8501997; doi:10.1093/nar/gkab798)
Supplement: gkab798_Supplemental_Files [file gkab798_supplemental_files.zip › STITCHIT_Supplement_NAR.pdf]

Supplementary Material

Supplementary Section 1 Data and Preprocessing

Accession numbers

Supplementary Table S9 lists the File IDs, Tissue/Cell type, Data type and Internal Sample ID of the raw data files obtained from Blueprint[1] and Roadmap [2]. Note that access to Blueprint and DEEP raw data files is restricted and requires an application to the Data Access Committee of the respective consortia.

Data preprocessing

We conducted two main data preprocessing steps: (1) quantification of gene-expression and (2) alignment of DNase1-seq data and identification of DNase hypersensitive sites.

The essential commands used for both steps along with software versions are presented in the next two sections.

RNA-seq processing

Gene-expression was quantified using Salmon [3], version 0.8.2., the Gencode [4] transcript index v26, and the Gencode genome annotation v26.

For single end reads, we used the command:

./salmon quant -i gencode.v26.transcripts.index/ -l A -r <Sample>_R1.fastq.gz -p 12 -o quants/<Sample> --seqBias --gcBias -g gencode.v26.annotation.gtf

For paired end reads, we used the command:

./salmon quant -i gencode.v26.transcripts.index/ -l A -1 <Sample>_R1.fastq.gz -2 <Sample>_R2.fastq.gz -p 12 -o quants/<Sample> --seqBias --gcBias -g gencode.v26.annotation.gtf

Discretized gene-expression values were computed using the POE method, see Supplementary Sec. 4 for details.

DNase1-seq processing

DNase1-seq reads were aligned to the hg38 reference genome with bowtie [5[, version 1.2.1.1, and samtools [6] version 1.2. For single end reads, this was done using the command call:

./bowtie --threads 10 -S GRCh38_no_alt/GCA_000001405.15_GRCh38_no_alt_analysis_set <Sample>_R1.fastq.gz | samtools view -b -o <Sample>.bam -) 2> Sample>.bowtie_statistics.txt

and for paired end reads using:

./bowtie --threads 10 -S GRCh38_no_alt/GCA_000001405.15_GRCh38_no_alt_analysis_set -1 <Sample>_R1.fastq.gz -2 <Sample>_R2.fastq.gz | samtools view -b -o <Sample>.bam -) 2 > <Sample> .bowtie_statistics.txt

In case that multiple fasta files exist for one sample, they were concatenated using the linux ***cat*** command prior to alignment, if applicable, separately per strand.

DNase Hypersensitive Sites (DHS) were identified with JAMM [7], version 1.0.7.5 and samtools version 1.2.

For single end reads, we use the commands:

bedtools bamtobed -i <Sample>.bam > JAMM-Input/<Sample>.bed

bash JAMM.sh –s JAMM-Input -g hg38_chrSize.txt –o <Sample>_peaks -f 1 -p 8

rm –r JAMM-Input/

For paired end reads, we run the commands:

```
samtools sort -n -O bam -@10 -T Bam-Sort-Pre <Sample>.bam | samtools view -bf 0x2 - | bedtools bamtobed -bedpe -i stdin > JAMM-Input/<Sample>.bed
```

```
bash JAMM.sh -s JAMM-Input -g hg38_chrSize.txt -o <Sample>_peaks -f 1 -p 8 -t paired
```

```
rm –r JAMM-Input/
```

As described in the JAMM github, we computed the enrichment of DNase1-seq signal within the peak by dividing column 7 by column 9 of the JAMM output files:

```
awk '{print $1\"\t\"$2\"\t\"$3\"\t\"$7/$9}'  <Sample>/peaks/filtered.peaks.narrowPeak > <Sample>/peaks/filtered.peaks.narrowPeak.adoptedCoverage
```

Bigwig files were generated using the DEEPtools [8] bamCoverage program, version 2.4.2 with the command

```
bamCoverage -b <Sample>.bam.sorted.bam –o <Sample>.bw -p 4 –normalizeUsingRPKM
```

Supplementary Section 2: Discretize expression data

We use the Probability Of Expression (POE) algorithm to discretize the gene-expression data [9]. Due to R computability and versioning issues, we had to rewrite some parts of the code. The entire R code is shown below. Note that the implementation of POE is also not designed to treat whole genome datasets. Therefore, we consider the data in batches, i.e. a subset of genes is discretized one after another.

```
library("metaArray")
library("Biobase")

args=commandArgs(TRUE)

myPOE<-function (AA, NN = NULL, id = NULL, M = 2000, kap.min = 3, logdata = FALSE,
                 stepsize = 0.5, centersample = FALSE, centergene = FALSE,
                 generatestarts = TRUE, start.method = 1, startobject = R0,
                 collapse.to.two = FALSE, burnin = 200, collapse.window = 50,
                 converge.threshold = 0.01, PR = list(alpha.mm = 0, alpha.sd = 100,
                                      mu.mm = 0, mu.sd = 100, pipos.mm = 0, pipos.sd = 100,
                                      pineg.mm = 0, pineg.sd = 100, kap.pri.rate = 1, tausqinv.aa = 1,
                                      tausqinv.bb = 0.1))


{
  TT <- ncol(AA)
  GG <- nrow(AA)
  tmp.poemat <- matrix(0, GG, TT)
  if (!is.null(NN) & length(NN) != TT) {
    stop("Length of NN should be the same as ncol(AA)")
  }
  if (is.na(sum(as.vector(AA))))
    stop("NA values not allowed in AA matrix. Remove rows with NA values and retry.")
  if (logdata == TRUE)
    AA <- log(AA)
  if (is.null(id) == TRUE)
    id <- as.character(1:GG)
  if (is.null(NN))
    NN <- rep(0, TT)
  if (centersample) {
    sammed <- apply(AA, 2, median)
    AA <- sweep(AA, 2, sammed)
  }

  if (centergene) {
    genmed <- apply(AA, 1, median)
    AA <- sweep(AA, 1, genmed)
  }
  if (generatestarts == TRUE) {
    alpha0 <- apply(AA, 2, mean)
    mats <- t(apply(AA, 1, sort))
    mug0 <- sigmag0 <- kappaposg0 <- kappanegg0 <- rep(NA,
                                                         GG)
    piposg0 <- pinegg0 <- rep(NA, GG)
    if (start.method == 1) {
      for (gg in 1:GG) {
        mug0[gg] <- mean(mats[gg, -c(1, 2, TT - 1, TT)])
        pinegg0[gg] <- piposg0[gg] <- 2/TT
        sigmag0[gg] <- sqrt(var(mats[gg, -c(1, 2, TT -
                                              1, TT)]))
        kappaposg0[gg] <- max(kap.min * sigmag0[gg],
                              mug0[gg] - mats[gg, 1])
        kappanegg0[gg] <- max(kap.min * sigmag0[gg],
                              mats[gg, TT] - mug0[gg])
      }
    }
    if (start.method == 2) {
      for (gg in 1:GG) {
        clu <- kmeans(mats[gg, ], 2)
        if (clu$size[1] == clu$size[2]) {
          big <- 1
          sma <- 2
        }
        else {
          big <- (1:2)[clu$size == max(clu$size)]
          sma <- (1:2)[clu$size == min(clu$size)]
        }
        mug0[gg] <- clu$centers[big]
        sigmag0[gg] <- sqrt(var(mats[gg, clu$cluster ==
                                       big]))
        if (mug0[gg] > 0) {
          pinegg0[gg] <- clu$size[sma]/TT
```

```
        piposg0[gg] <- 1/TT
      }
      else {
        piposg0[gg] <- clu$size[sma]/TT
        pinegg0[gg] <- 1/TT
      }
      kappaposg0[gg] <- max(max(mats[gg, ]) - mug0[gg],
                           kap.min * sigmag0[gg])
      kappanegg0[gg] <- max(mug0[gg] - min(mats[gg,
                                         ]), kap.min * sigmag0[gg])
    }
  }
  if (start.method == 3) {
    for (gg in 1:GG) {
      clu <- kmeans(mats[gg, ], 3)
      posc <- (1:3)[clu$centers == max(clu$centers)]
      negc <- (1:3)[clu$centers == min(clu$centers)]
      midc <- (1:3)[clu$centers == median(clu$centers)]
      mug0[gg] <- clu$centers[midc]
      sigmag0[gg] <- sqrt(var(mats[gg, clu$cluster ==
                                midc]))
      pinegg0[gg] <- clu$size[negc]/TT
      piposg0[gg] <- clu$size[posc]/TT
      kappaposg0[gg] <- max(max(mats[gg, ]) - mug0[gg],
                           kap.min * sigmag0[gg])
      kappanegg0[gg] <- max(mug0[gg] - min(mats[gg,
                                         ]), kap.min * sigmag0[gg])
    }
  }
  if (start.method == 4) {
    if (sum(NN) < 3)
      stop("start.method==4 requires at least 3 normal samples")
    for (gg in 1:GG) {
      mug0[gg] <- mean(AA[gg, NN == 1])
      sigmag0[gg] <- sqrt(var(AA[gg, NN == 1]))
      clu <- kmeans(AA[gg, ], 2)
      norm <- (1:2)[abs(clu$centers - mug0[gg]) ==
                    min(abs(clu$centers - mug0[gg]))]
 expr <- 1
      if (norm == 1)
        expr <- 2
      if (clu$centers[norm] > clu$centers[expr]) {
        pinegg0[gg] <- min(clu$size[expr]/TT, 1/2)
        piposg0[gg] <- 1/TT
      }
      else {
        piposg0[gg] <- min(clu$size[expr]/TT, 1/2)
        pinegg0[gg] <- 1/TT
      }
      kappaposg0[gg] <- max(max(AA[gg, ]) - mug0[gg],
                           kap.min * sigmag0[gg])
      kappanegg0[gg] <- max(mug0[gg] - min(AA[gg, ]),
                           kap.min * sigmag0[gg])
    }
  }
  sigmagsqinv0 <- 1/sigmag0^2
  gamma0 <- (mean(sigmagsqinv0))^2/var(sigmagsqinv0)
  lambda0 <- mean(sigmagsqinv0)/var(sigmagsqinv0)
  tmpmat <- matrix(0, GG, TT)

  PP <- list(alpha = alpha0, mug = mug0, kappaposg = kappaposg0,
             kappanegg = kappanegg0, sigmag = sigmag0, piposg = piposg0,
             pinegg = pinegg0, mu = mean(mug0), tausqinv = 1/var(mug0),
             gamma = gamma0, lambda = lambda0, pil.pos.mean = mean(logit(piposg0)),
             pil.neg.mean = mean(logit(pinegg0)), pil.pos.prec = mean(piposg0) *
               (1 - mean(piposg0)), pil.neg.prec = mean(pinegg0) *
               (1 - mean(pinegg0)), kap.pos.rate = 1/mean(kappaposg0),
             kap.neg.rate = 1/mean(kappanegg0), poe = tmp.poemat,
             phat.pos = tmpmat, phat.neg = tmpmat, accept = 0)
 }
 if (generatestarts == FALSE) {
  mmm <- length(startobject$gamma)
  PP <- list(alpha = startobject$alpha[mmm, ], mug = startobject$mug[mmm,
                                                ], kappaposg = startobject$kappaposg[mmm, ], kappanegg = startobject$kappanegg[mmm,
                                                                                        ], sigmag = startobject$sigmag[mmm, ],
piposg = startobject$piposg[mmm,
                                                                                                                            ],
pinegg = startobject$pinegg[mmm, ], mu = startobject$mu[mmm],
             tausqinv = startobject$tausqinv[mmm], gamma = startobject$gamma[mmm],
             lambda = startobject$lambda[mmm], pil.pos.mean = startobject$pil.pos.mean[mmm],
             pil.pos.prec = startobject$pil.pos.prec[mmm], pil.neg.mean = startobject$pil.neg.mean[mmm],
             pil.neg.prec = startobject$pil.neg.prec[mmm], kap.pos.rate = startobject$kap.pos.rate[mmm],
             kap.neg.rate = startobject$kap.neg.rate[mmm], poe = tmp.poemat,
```

```
            phat.pos = tmpmat, phat.neg = tmpmat, accept = 0)
  }
  if (collapse.to.two) {
    cat("Collapsing to Two: ")
    cw <- collapse.window
    mm <- 0
    pidif <- 1
    pim.pos <- pim.neg <- 100
    while (pidif > converge.threshold) {
      mm <- mm + 1
      new.PR <- unlist(PR)
      new.PP <- unlist(PP)
      avgpos <- rep(0, GG * (3 * TT + 6) + TT + 11)
      res <- .C("poe_fit_2", as.matrix(AA), as.integer(NN),
              as.double(new.PR), as.double(new.PP), as.integer(GG),
              as.integer(TT), as.integer(1), avgpos = as.double(avgpos),
              PACKAGE = "metaArray")$avgpos
      PP$alpha <- res[1:TT]
      PP$mug <- res[(TT + 1):(TT + GG)]
      PP$kappaposg <- res[(TT + GG + 1):(TT + 2 * GG)]
      PP$kappanegg <- res[(TT + 2 * GG + 1):(TT + 3 * GG)]
      PP$sigmag <- res[(TT + 3 * GG + 1):(TT + 4 * GG)]
      PP$piposg <- res[(TT + 4 * GG + 1):(TT + 5 * GG)]
      PP$pinegg <- res[(TT + 5 * GG + 1):(TT + 6 * GG)]
      PP$mu <- res[TT + 6 * GG + 1]
      PP$tausqinv <- res[TT + 6 * GG + 2]
      PP$gamma <- res[TT + 6 * GG + 3]
      PP$lambda <- res[TT + 6 * GG + 4]
      PP$pil.pos.mean <- res[TT + 6 * GG + 5]
      PP$pil.neg.mean <- res[TT + 6 * GG + 6]
      PP$pil.pos.prec <- res[TT + 6 * GG + 7]
      PP$pil.neg.prec <- res[TT + 6 * GG + 8]
      PP$kap.pos.rate <- res[TT + 6 * GG + 9]
      PP$kap.neg.rate <- res[TT + 6 * GG + 10]
      PP$poe <- matrix(res[(TT + 6 * GG + 11):(GG * (TT +
                                    6) + TT + 10)], nrow = GG)
      PP$phat.pos <- matrix(res[(GG * (TT + 6) + TT + 11):(GG *
                                       (2 * TT + 6) + TT + 10)], nrow = GG)
      PP$phat.neg <- matrix(res[(GG * (2 * TT + 6) + TT +
                         11):(GG * (3 * TT + 6) + TT + 10)], nrow = GG)
      PP$accept <- res[GG * (3 * TT + 6) + TT + 11]
      gc()
      pim.pos <- c(pim.pos, mean(PP$piposg))
      pim.neg <- c(pim.neg, mean(PP$pinegg))
      if (mm > (2 * cw)) {
        pidif <- 0.5 * max(abs(mean(pim.neg[(mm - cw -
                                   1):(mm)]) - mean(pim.neg[(mm - 2 * cw - 1):(mm -
                                                  cw)]))/(mean(pim.neg[(mm - cw - 1):(mm)]) +
                                                     mean(pim.neg[(mm - 2 * cw - 1):(mm - cw)])),
                  abs(mean(pim.pos[(mm - cw - 1):(mm)]) - mean(pim.pos[(mm -
                                              2 * cw - 1):(mm - cw)]))/(mean(pim.pos[(mm -
                                                           cw - 1):(mm)]) + mean(pim.pos[(mm - 2 * cw -
                                                                         1):(mm - cw)])))
      }
    }
 for (gg in 1:GG) {
      ppplus <- mean(PP$phat.pos[gg, ])
      ppminu <- mean(PP$phat.pos[gg, ] - PP$poe[gg, ])
      pp0 <- 1 - ppminu - ppplus
      if (ppplus < 2/TT & abs(ppminu - pp0) < 6/TT) {
        PP$phat.pos[gg, ] <- abs(PP$poe[gg, ])
        PP$poe[gg, ] <- abs(PP$poe[gg, ])
        ee <- round(PP$poe[gg, ])
        PP$mug[gg] <- mean(AA[gg, ee == 0], na.rm = TRUE)
        PP$piposg[gg] <- mean(ifelse(ee == 1, 1, 0))
        PP$pinegg[gg] <- mean(ifelse(ee == -1, 1, 0))
        PP$sigmag[gg] <- sqrt(var(AA[gg, ee == 0], na.rm = TRUE))
        PP$kappanegg[gg] <- kap.min * PP$sigmag[gg]
        PP$kappaposg[gg] <- kap.min * PP$sigmag[gg]
      }
    }
    cat("Finished\\n")
  }
  cat("Gibbs Sampler\n")
  new.PR <- unlist(PR)
  new.PP <- unlist(PP)
  avgpos <- rep(0, GG * (3 * TT + 6) + TT + 11)
  cat("First\n")
  res <- .C("poe_fit", as.matrix(AA), as.integer(NN), as.double(new.PR),
          as.double(new.PP), as.integer(GG), as.integer(TT), as.integer(M),
          as.integer(burnin), avgpos = as.double(avgpos), PACKAGE = "metaArray")$avgpos
 cat("Second\n")
  alpha <- res[1:TT]
```

```
    mug <- res[(TT + 1):(TT + GG)]
    kappaposg <- res[(TT + GG + 1):(TT + 2 * GG)]
    kappanegg <- res[(TT + 2 * GG + 1):(TT + 3 * GG)]
    sigmag <- res[(TT + 3 * GG + 1):(TT + 4 * GG)]
    piposg <- res[(TT + 4 * GG + 1):(TT + 5 * GG)]
    pinegg <- res[(TT + 5 * GG + 1):(TT + 6 * GG)]
    mu <- res[TT + 6 * GG + 1]
    tausqinv <- res[TT + 6 * GG + 2]
    gamma <- res[TT + 6 * GG + 3]
    lambda <- res[TT + 6 * GG + 4]
    pil.pos.mean <- res[TT + 6 * GG + 5]
    pil.neg.mean <- res[TT + 6 * GG + 6]
    pil.pos.prec <- res[TT + 6 * GG + 7]
    pil.neg.prec <- res[TT + 6 * GG + 8]
    kap.pos.rate <- res[TT + 6 * GG + 9]
    kap.neg.rate <- res[TT + 6 * GG + 10]
    cat("Three\n")
    poe <- matrix(res[(TT + 6 * GG + 11):(GG * (TT + 6) + TT +
                                         10)], nrow = GG)
    poe <- as.data.frame(poe)
    rownames(poe) <- rownames(AA)
    colnames(poe) <- colnames(AA)
    accept <- res[GG * (3 * TT + 6) + TT + 11]
    gc()
        cat("Four")
    foo <- function(org, fit, inter = 0.01) {
      ord <- order(org)
      org.t <- org[ord]
      fit.t <- fit[ord]
      l <- length(org)
      min.fit <- min(fit.t)
      i <- 1
      while (fit.t[i] - min.fit > inter) {
        fit.t[i] <- min.fit
        i <- i + 1
      }
      i <- length(org)
      max.fit <- max(fit.t)
      while (max.fit - fit.t[i] > inter) {
        fit.t[i] <- max.fit
        i <- i - 1
      }
      fit2 <- fit
      fit2[ord] <- fit.t
      fit2
    }
    for (i in 1:GG) {
      poe[i, ] <- foo(as.numeric(AA[i, ]), as.numeric(poe[i,
                                                    ]))
    }
    return(list(alpha = alpha, mug = mug, kappaposg = kappaposg,
                kappanegg = kappanegg, sigmag = sigmag, piposg = piposg,
                pinegg = pinegg, mu = mu, tausqinv = tausqinv, gamma = gamma,
                lambda = lambda, pil.pos.mean = pil.pos.mean, pil.pos.prec = pil.pos.prec,
                pil.neg.mean = pil.neg.mean, pil.neg.prec = pil.neg.prec,
                kap.pos.rate = kap.pos.rate, kap.neg.rate = kap.neg.rate,
                poe = poe, accept = accept))
  }



data<-read.table(args[1],header=T,row.names=1,stringsAsFactors = F)
medGenes<-apply(data,1,median)
data<-data[-which(medGenes == 0),]
batchSize=2000
nbatchs=dim(data)[1]/batchSize
for (i in c(1:ceiling(nbatchs))){

        subset<-data[c((((i-1)*batchSize)+1):min(i*batchSize,dim(data)[1])),]
        print(paste0("Discretizing batch : ",(i-1)*batchSize+1," to ",min(i*batchSize,dim(data)[1])))
        poe.Result_CenterBoth_2000<-myPOE(log(as.matrix((subset))+1),M=2000,centergene = TRUE, centersample = TRUE)
        write.table(round(poe.Result_CenterBoth_2000$poe,digits=0),file=paste0("Discretised_Data_Batch_",i,".txt"),quote=FALSE,row.names =
        TRUE, col.names=NA,sep="\t")
}
```

Supplementary Section 3: STITCHIT algorithm

Running the STITCHIT algorithm

To run STITCHIT, the user needs to provide discretized (-d) and original expression data (-o), a gene annotation file (-a), a chromosome size file (-s), as well as big wig files with the epigenetic signal to consider (-b). Using the command:

./build/core/STITCHIT –b <Consortium>/DNase_bw/ -a ../../../nobackup/References/gencode.v26.annotation.gtf -d <Consortium>/_Discretised_Complet.txt -o <Consortium>_expression.txt -s data/hg38_chrSize.txt -w 25000 -c 12 -p 0.05 -g <geneID> -z 10 -f ../../../archive00/Segmentation_<Consortium>/ -r 500000 -t 2000

a call to STITCHIT can be invoked. The parameter –w denotes the size of the window extension up an downstream of the gene, -c denotes the number of used CPU, -p is the significance threshold for the correlation test, -g is the parameter to denote the target gene ID, -z indicates the width of the initial binning, -f denotes the output path, -r is the maximum size of the entire search region and –t refers to the maximum size of a segment. Further details on the parameters are provided in the repository (www.github.com/Schulzlab/Stitchit).

General Setup of the Data Matrix processed in Steps (c) to (f) in Figure S2

The extracted the data matrix $D_{i,j}$ consists of m rows corresponding to the samples and n columns that correspond to the base pair positions. In addition, each sample is associated with a class label according to its expression value. With C_k we relate to all rows that are assigned to label k. We initially group the base pairs into segments of size β, which is a user defined parameter that we set to set $\beta = 5$ in our experiments. Setting β to a larger value would speed up the runtime, however, since no segment can be smaller than β, increasing it could lead to missing out segments.

Example

The following example showcases a run of the STITCHIT algorithm on a data matrix consisting of six samples with six initial segments ($\beta = 1$). The example tries to find a segmentation that best explains the expression difference for two classes, labelled 1 and 2. Each class has been assigned to three samples.

Data matrix $D_{i,j}$

	**b1**	**b2**	**b3**	**b4**	**b5**	**b6**	**exp**
**s1**	10	9	1	1	7	8	1
**s2**	11	10	2	1	7	8	1
**s3**	10	8	1	0	9	8	1
**s4**	2	4	25	24	0	1	2
**s5**	3	5	23	22	1	0	2
**s6**	2	6	26	25	0	1	2

From the data matrix, we compute the sum S and sum of squares of the values of each column of D per class.

$$S_k[j] = S[j-1] + \sum_{i \in C_k} d_{i,j}$$

$$SS_k[j] = SS[j-1] + \sum_{i \in C_k} d_{i,j}^2$$

Sum per class S_k:

**$S_1$1**	**$S_1$2**	**$S_1$3**	**$S_1$4**	**$S_1$5**	**$S_1$6**
31	58	62	64	87	111

$S_2 1$	$S_2 2$	$S_2 3$	$S_2 4$	$S_2 5$	$S_2 6$
7	22	96	167	168	170

Sum of squares per class SS_k

$SS_1 1$	$SS_1 2$	$SS_1 3$	$SS_1 4$	$SS_1 5$	$SS_1 6$
321	566	572	574	753	945

$SS_2 1$	$SS_2 2$	$SS_2 3$	$SS_2 4$	$SS_2 5$	$SS_2 6$
17	94	1924	3609	3610	3612

To calculate the data costs $w_{i,j}^k$ for each hypothetical segment from position i to j, with $i \leq j$, we need to compute the empirical standard deviation $\hat{\sigma}_{i,j}^k$ as well as the number of data points within a bin $n_{i,j}^k$. This we can do in constant time using the precomputed vectors S_k and SS_k [10], where

$$\hat{\sigma}_{i,j}^k = \sqrt{\frac{1}{n_{i,j}^k}\left((SS_k[j] - SS_k[i-1]) - \frac{1}{n_{i,j}^k}(S_k[j] - S_k[i-1])^2\right)},$$

$$n_{i,j}^k = (j + 1 - i) * |C_k|,$$

where $|C_k|$ denotes the number of samples related to class k.

Data points per segment $n_{i,j}$

i, j	1	2	3	4	5	6
1	3	6	9	12	15	18
2	0	3	6	9	12	15
3	0	0	3	6	9	12
4	0	0	0	3	6	9
5	0	0	0	0	3	6
6	0	0	0	0	0	3

The matrix $n_{i,j}$ is identical for both classes.
Standard deviation class 1 $\hat{\sigma}_{i,j}^1$

i, j	1	2	3	4	5	6
1	0.471405	0.942809	4.012327	4.403282	4.069398	3.804237
2	0	0.816497	3.890873	3.829708	3.771236	3.627059
3	0	0	0.471405	0.57735	3.224137	3.47511
4	0	0	0	0.471405	3.578485	3.435472
5	0	0	0	0	0.942809	0.687184
6	0	0	0	0	0	0

Standard deviation class 2 $\hat{\sigma}^2_{i,j}$

i,j	1	2	3	4	5	6
1	0.471405	1.490712	10	10.34777	10.73437	10.55789
2	0	0.816497	9.889669	9.113821	10.92748	11.02643
3	0	0	1.247219	1.34371	11.29186	11.87668
4	0	0	0	1.247219	11.7047	10.9522
5	0	0	0	0	0.471405	0.5
6	0	0	0	0	0	0.471405

From $\hat{\sigma}^k_{i,j}$ and $n^k_{i,j}$, we then compute the data costs $w^k_{i,j}$ according to the MDL encoding for Gaussian distributed data points [11]. For notational convenience, we write s to denote to the segment according to the range i, j. Hence, we compute the data costs for segment s and class label k as

$$L(D|s,k) = w^k_{i,j} = \frac{n^k_{i,j}}{2}\left(\frac{1}{\log(2)} + \log_2(2\pi\hat{\sigma}^k_{i,j}\hat{\sigma}^k_{i,j})\right) + n^k_{i,j}\log_2\tau,$$

Where τ is the data resolution. To simplify the example, we set $\tau = 1$. As we only work with integers setting $\tau = 1$ is sufficient, however, since we later on also encode the mean values for each segment, which can be a floating point number, we set $\tau = 0.1$ in all our experiments.

Scores for class 1 $w^1_{i,j}$

i,j	1	2	3	4	5	6
1	5.705249	17.4105	44.92036	61.50349	75.17291	88.45775
2	0	8.082693	29.68083	44.31552	58.821	72.68268
3	0	0	5.705249	13.16539	42.08063	57.40525
4	0	0	0	5.705249	28.95637	42.90498
5	0	0	0	0	8.705249	14.67289
6	0	0	0	0	0	0

Scores for class 2 $w_{i,j}^{2}$

i,j	1	2	3	4	5	6
1	5.705249	21.37628	56.77776	76.29552	96.16316	114.9653
2	0	8.082693	37.75581	55.57291	77.2392	96.7441
3	0	0	9.916281	20.4776	58.35531	78.68126
4	0	0	0	9.916281	39.21437	57.95874
5	0	0	0	0	5.705249	11.92027
6	0	0	0	0	0	5.705249

Next, we compute the total cost matrix W, defined as

$W_{i,j} = \sum_{l=1..k} \frac{w_{i,j}^{k}}{|C_k|}$,

Total cost matrix *W:*

i,j	1	2	3	4	5	6
1	3.803499	12.92893	33.89937	45.93301	57.11203	67.80769
2	0	5.388462	22.47888	33.29614	45.3534	56.47559
3	0	0	5.207177	11.21433	33.47865	45.36217
4	0	0	0	5.207177	22.72358	33.62124
5	0	0	0	0	4.803499	8.864386
6	0	0	0	0	0	1.90175

Using W, we conduct the dynamic programming approach as described by Nguyen and Vreeken [12] to find the optimal segmentation for each possible number of segments according to our cost function, following the recursion formula

$$c_{i,j} = \min_{k=1,\dots,j-1} (c_{i-1,k} + W_{k+1,j})$$

i,j	1	2	3	4	5	6
1	3.803499	12.92893	33.89937	45.93301	57.11203	67.80769
2	0	9.191961	18.1361	24.14326	46.40757	54.79739
3	0	0	14.39914	20.40629	28.94675	33.00764
4	0	0	0	19.60631	25.20979	29.27068
5	0	0	0	0	24.40981	28.4707
6	0	0	0	0	0	26.31156

Subsequently, we compute the costs for the models of a segmentation S consisting of $|S|$ segments according to:

$$L(S) = L_{\mathbb{N}}(|S|) + |C||S|log_2\left(\frac{|\max - \min|}{\tau}\right) + log_2\binom{n-1}{|S|-1},$$

Where $|C|$ is the number of possible class labels (in this example $|C| = 2$).

Costs for the binning:

**Level 1**	10.91945
**Level 2**	23.64225
**Level 3**	35.29254
**Level 4**	45.44401
**Level 5**	54.66348

**Level 6**	62.33327

Combined with the last column of the dynamic programing matrix this yields the final costs:

**Level 1**	78.72714
**Level 2**	78.43965
**Level 3**	**68.30019**
**Level 4**	74.71469
**Level 5**	83.13418
**Level 6**	88.64484

Here, the minimum cost is associated to the third level.

Performing backtracking in the dynamic programming matrix yields the segmentation

$$b_{1,2}, b_{3,4}, b_{5,6}.$$

The corresponding points in the matrix are marked in red.

**i,j**	**1**	**2**	**3**	**4**	**5**	**6**
**1**	3.803499	**12.92893**	33.89937	45.93301	57.11203	67.80769
**2**	0	9.191961	18.1361	**24.14326**	46.40757	54.79739
**3**	0	0	14.39914	20.40629	28.94675	**33.00764**
**4**	0	0	0	19.60631	25.20979	29.27068
**5**	0	0	0	0	24.40981	28.4707
**6**	0	0	0	0	0	26.31156

Supplementary Section 4 Additional gene-enhancer linkage approaches

Unsupervised window based linkage

For each gene g in each sample i we compute the number of DNase Hypersensitive sites c_i^g, the length of DHSs l_i^g, as well as the signal within the DHSs s_i^g according to:

$$c_s^g = \sum_{p \in P_{w,g}} I(p) * \exp \frac{(-dist(d_{p,g})}{d_0}),$$

$$l_s^g = \sum_{p \in P_{w,g}} |p| * \exp\left(-dist(d_{p,g})/d_0\right),$$

$$s_s^g = \sum_{p \in P_{w,g}} s(p) * \exp \frac{(-dist(d_{p,g})}{d_0}).$$

Here, I is the indicator function, $|p|$ denotes the length of p, the function $s(p)$ denotes the signal within p. $P_{w,g}$ denotes the set of DHSs p within a window w around the TSS of gene g. As shown in Figure S2, we consider three different windows w: (1) a window of size 5kb centered at the TSS of gene g, (2) a window of size 50kb centered at the TSS of gene g, (3) a window considering a 2.5kb upstreaming region from the TSS of gene g combined with the entire gene-body of g. The input matrix of the linear regression is composed of the following terms:

	Count DHSs	Length DHSs	Signal DHSs
Sample 1	c_1^g	l_1^g	s_1^g
...			
Sample n	c_n^g	l_n^g	s_n^g

We compute these quantities using the TEPIC [13] tool using the following commands

5kb:

bash TEPIC.sh -g hg38.noPrefix.masked.fa -b <Sample>_peaks/peaks/filtered.peaks.narrowPeak.adoptedCoverage -o <Sample>_5kb -p ../PWMs/human_jaspar_hoc_kellis.PSEM -c 12 -n 4 -w 5000 -a gencode.v26.annotation.gtf -f gencode.v26.annotation.gtf -q TRUE

50kb:

bash TEPIC.sh -g hg38.noPrefix.masked.fa -b <Sample>_peaks/peaks/filtered.peaks.narrowPeak.adoptedCoverage -o <Sample>_50kb -p ../PWMs/human_jaspar_hoc_kellis.PSEM -c 12 -n 4 -w 50000 -a gencode.v26.annotation.gtf -f gencode.v26.annotation.gtf -q TRUE

gene body:

bash TEPIC.sh -g hg38.noPrefix.masked.fa -b <Sample>_peaks/peaks/filtered.peaks.narrowPeak.adoptedCoverage -o <Sample>_gB -p ../PWMs/human_jaspar_hoc_kellis.PSEM -c 12 -n 4 -w 5000 -a gencode.v26.annotation.gtf -f gencode.v26.annotation.gtf -q TRUE -y

The workflow is depicted in Figure S4a. Details on the parameters are provided in the TEPIC repository (www.github.com/Schulzlab/TEPIC).

Promoters and Enhancers extracted from GeneHancer

We downloaded the collection of all regulatory elements contained in the GeneHancer database [14], obtained at 6th August, 2018. Using an adapted version of the tool presented in this work, we compute the DNase1-seq signal within each of these regions and identify relevant segments using a correlation test. This yields a per-gene input matrix of the following form:

	DNase1-seq signal in GeneHancer site 1	…	DNase1-seq signal in GeneHancer site m
Sample 1	g_1^1		g_1^m
...			
Sample n	g_n^1		g_n^m

Note that this approach does not depend on any size or regions cut-offs.

We provide a script to parse the GeneHancer tsv file into a simple tab delimited format:

chr ***tab*** *start* ***tab*** *end* ***tab*** *ENSG-ID* ***tab*** *gene name*

The script can be used with the command:

python rewriteGeneHancer.py <Original GeneHancer dump> ENSGIds_GeneName.txt > <Destination.txt>

Subsequently, the candidate regions can be computed via:

./build/core/GENEHANCER -b <Consortium>/DNase_bw/ -a /gencode.v26.annotation.gtf -o <Consortium>_expression.txt -s data/hg38_chrSize.txt -w 25000 -p 0.05 -g <geneID> -f GeneHancer_<Consortium> -r 500000 –k genehancer_database_06.07.2018_EnsembleMatch.sorted.bed

Here, <Consortium> is a placeholder for the data source, and <geneID> is a placeholder for the target gene. Details on the parameters are provided in the repository (www.github.com/Schulzlab/Stitch).

The workflow is depicted in Figure S4b.

Unification of DNase hypersensitive sites

Using *bedtools* [15], we combined all DHSs within a window w centered at the TSS of the target gene g into several merged sites MS^m. Within these sites, we compute the average DNase1-seq signal using an adapted version of the STITCHIT algorithm and identify sites that are correlated to the expression of gene g. This yields a feature matrix as shown below.

	DNase1-seq signal in unified site 1	…	DNase1-seq signal in unified site m
Sample 1	u_1^1		u_1^m
...			
Sample n	u_n^1		u_n^m

We used the linux *cat* and *sort* commands together with the *bedtools merge* command

cat <Consortium>/*/peaks/filtered.peaks.narrowPeak.adoptedCoverage

sort -s -k1,1 -k2,2n Merged_<Consortium>.bed > Merged_<Consortium>.sorted.bed

bedtools merge -i Merged_<Consortium>.sorted.bed > Merged_<Consortium>.sorted.merged.bed

to generate the merged DHS sites and subsequently generated the feature matrices using:

./build/core/UNIFIED_PEAKS -b <Consortium>/DNase_wig_Normalized/ -a gencode.v26.annotation.gtf -o <Consortium>_expression.txt -s data/hg38_chrSize.txt -w 25000 -p 0.05 -g <geneID> -f UnifiedPeaks_<Consortium> -r 500000 -k Merged_<Consortium>_Peaks.sorted.merged.bed

The workflow is depicted in Figure S4c. Details on the parameters are provided in the repository (www.github.com/Schulzlab/Stitchit).

Supplementary Section 5: Supplementary Figures & Tables

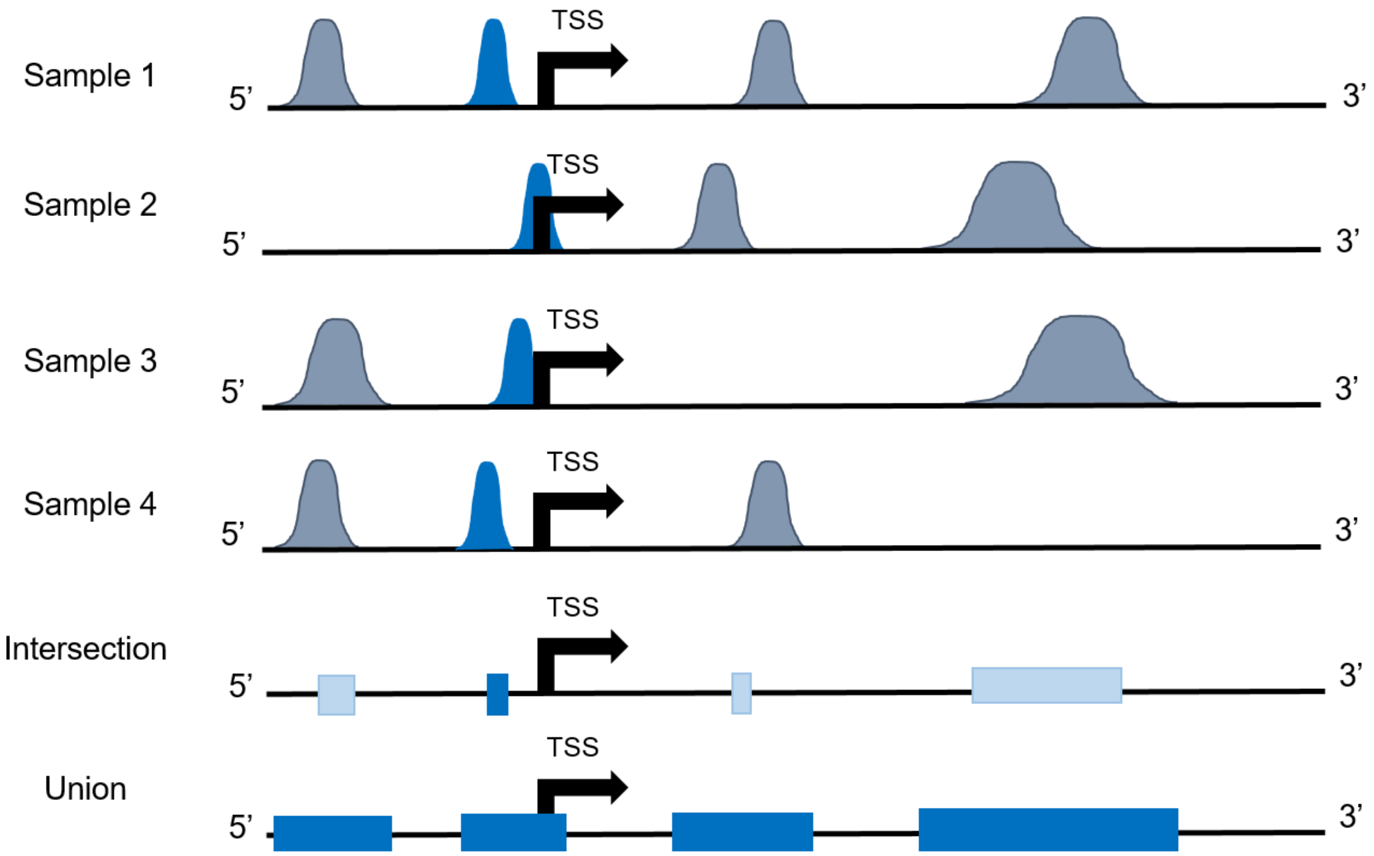

Supplementary Figure S1: This Figure illustrates two common ways to integrate DHS sites across multiple samples. In the intersection, only the accessible portion of the genome across all samples is available. In the union case, all overlapping DHS sites are merged. The latter case preserves a large portion of the accessible chromatin, but it loses the possible varying position of the DHSs in the data. The intersection on the other hand might be too conservative.

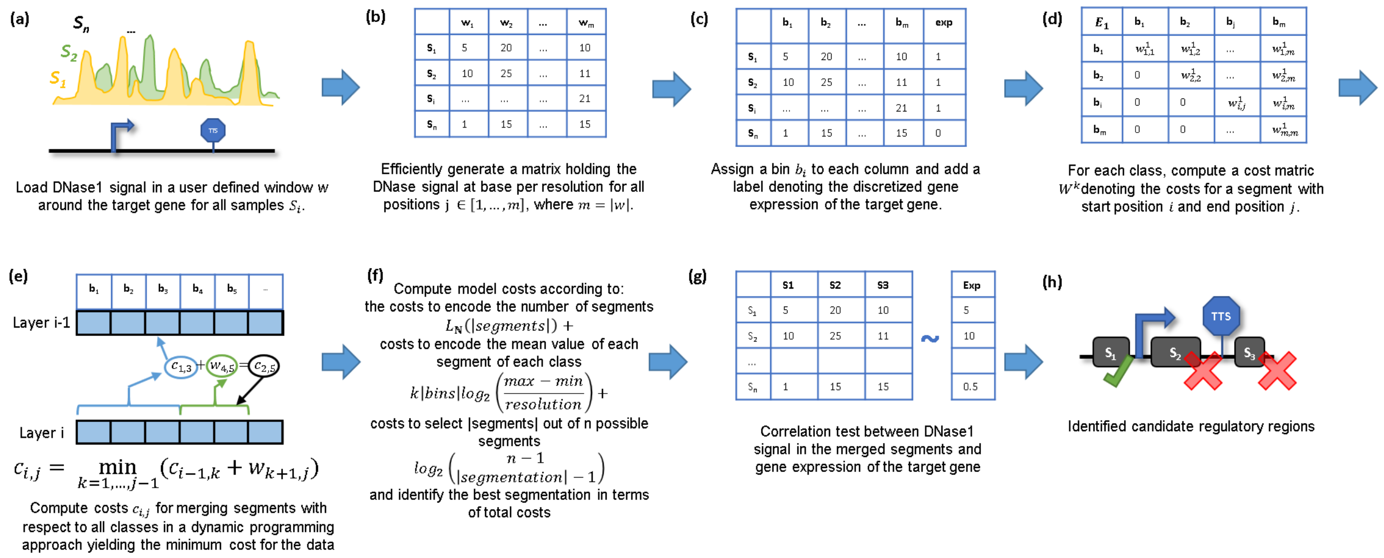

Supplementary Figure S2: Schematic overview on the STITCHIT algorithm. (a) Upon data generation we load DNase1-seq and gene-expression data per gene across for all available samples. (b) Using the C-library *libBigWig*, we efficiently generate a data matrix holding the DNase1-seq data at base-pair resolution. (c) We divide the data into x distinct classes referring to the discrete expression value of the target gene across the respective samples. (d) For each expression class, we compute a cost matrix that denotes the costs for generating every possible binning. (e) The optimal solution with the minimum cost is computed in a dynamic programming approach, yielding the costs of the data given a certain model (*L(D|M)*). (f) Following the MDL principle, the costs for the actual model (*L(M)*) are computed according to default formulas. (g) Via a correlation test with the continuous gene-expression data, significantly associated segments are identified, yielding one segment associated to gene expression (h).

(a)

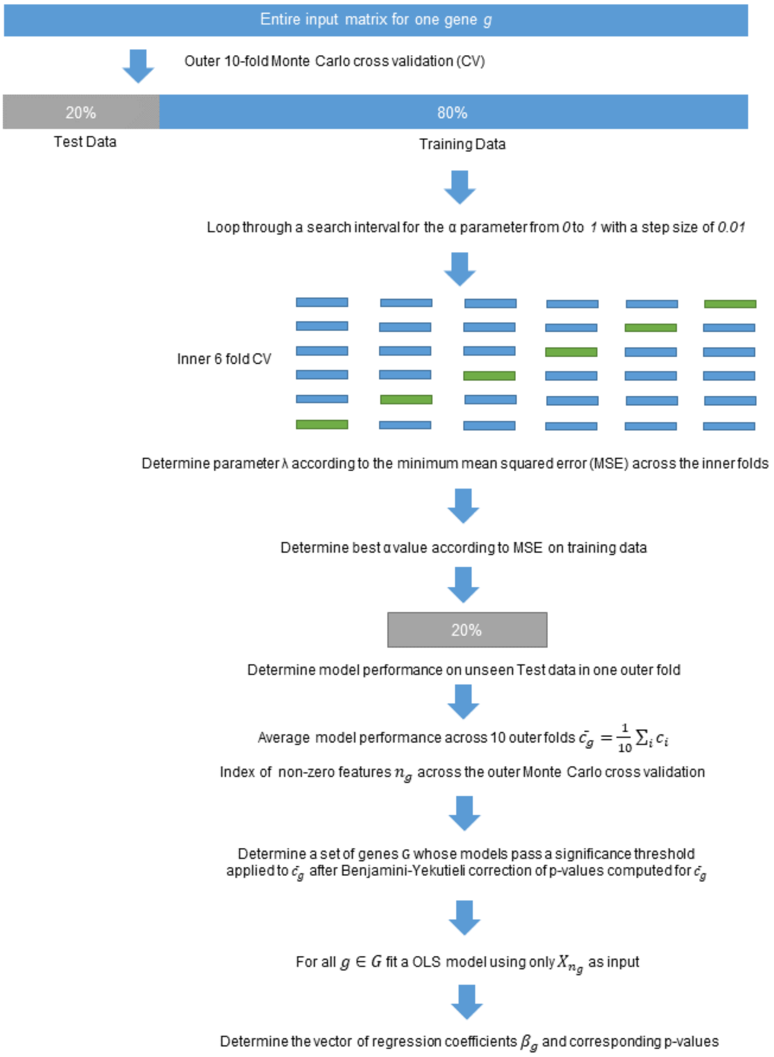

(b)

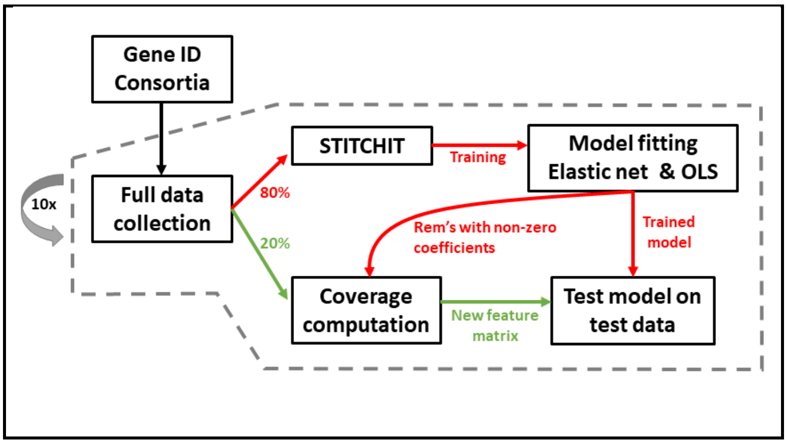

Supplementary Figure S3: a) Two level learning approach used in the manuscript. Using elastic net regularization, a set of candidate features per gene is selected. For all models achieving a sufficient model performance that is passing a significance test and a p-value correction according to Benjamini-Yekutieli, an OLS model is fitted to learn the final coefficient values and their corresponding p-values. b) Execution of the STITCHIT algorithm using a nested feature selection on 80% of the entire data set in a 10 fold Monte Carlo cross-validation procedure.

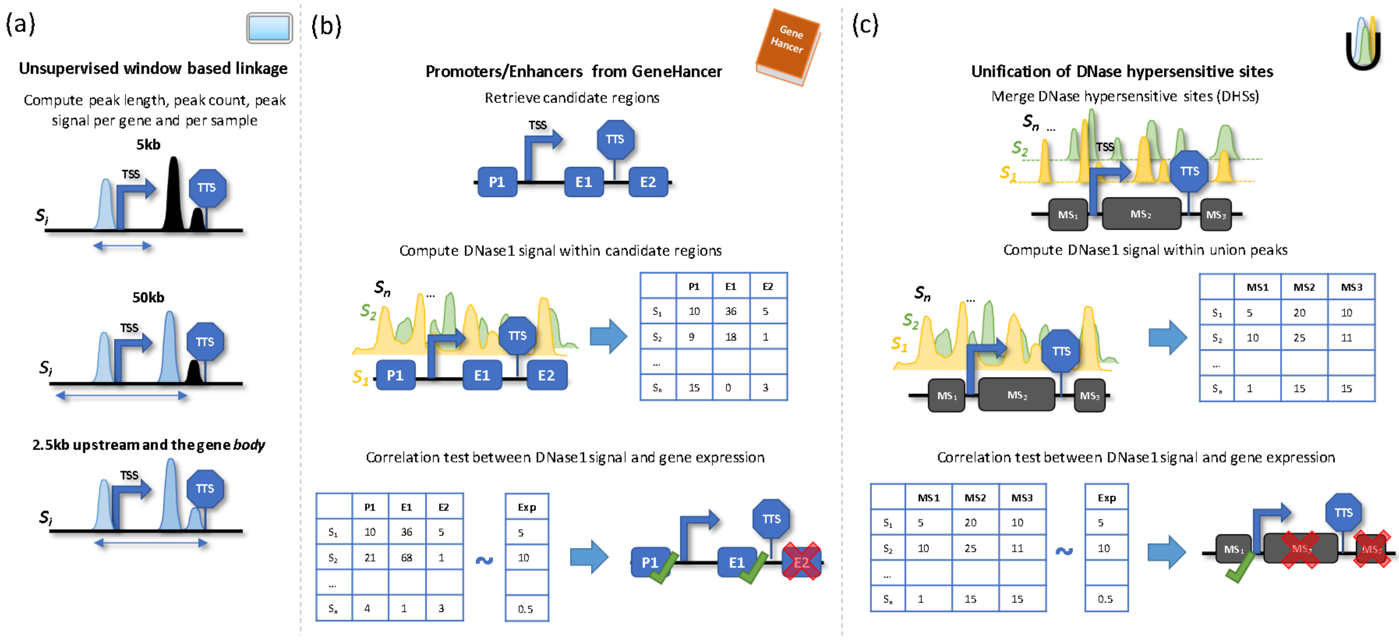

Supplementary Figure S4 Overview on additional gene-enhancer linkage approaches. Following a window based approach, as normally applied in per sample models (a), we assess length, count and signal of DNase hypersensitive sites (DHSs) per gene and per sample using three different windows: 5kb, 50kb, and a gene body window. Also, we consider a curated set of promoters and enhancers contained in the GeneHancer database (b). Here, we select regions that exhibit DNase1-seq signal that correlates with the expression of a target gene. Another approach is depicted in (c). Here, we identify the union of all DHS sites and select regions that exhibit DNase1-seq signal that correlates with the expression of the target gene.

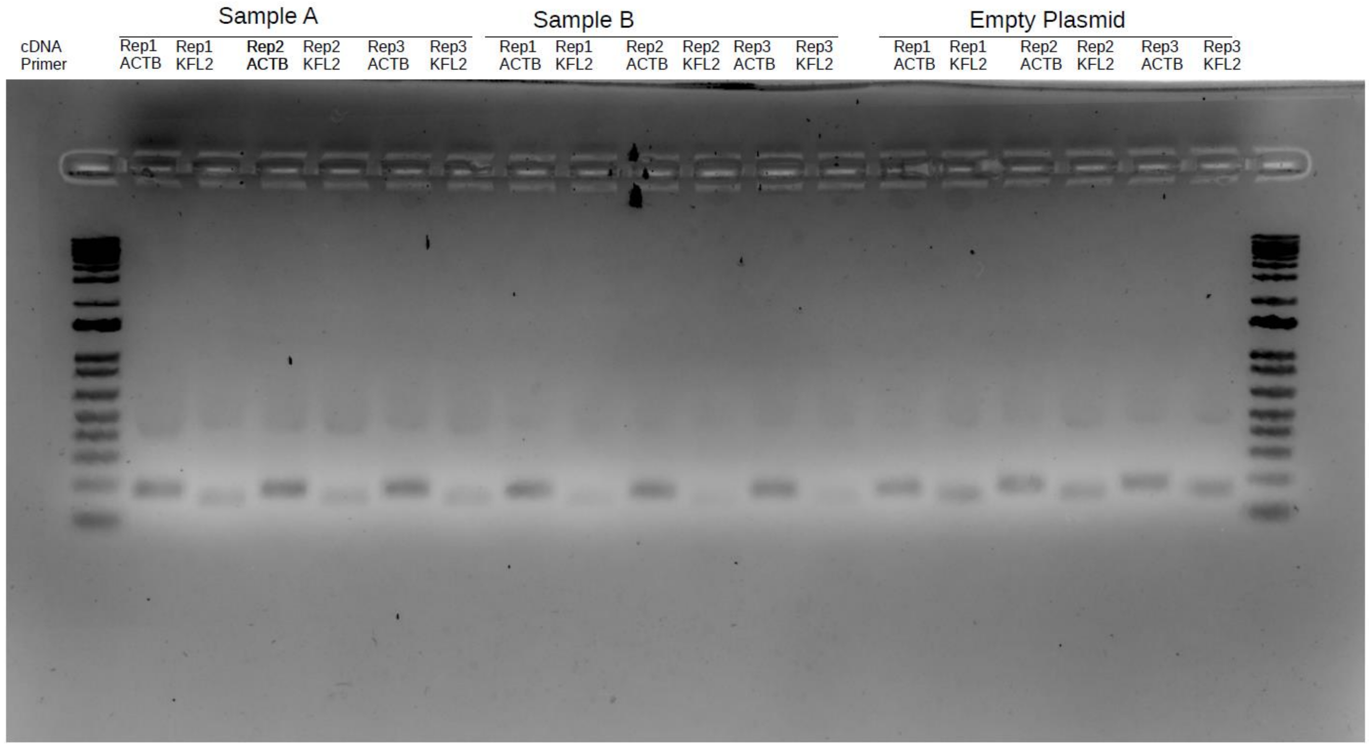

Supplementary Figure S5: PCR results for experiments targeting KLF2.

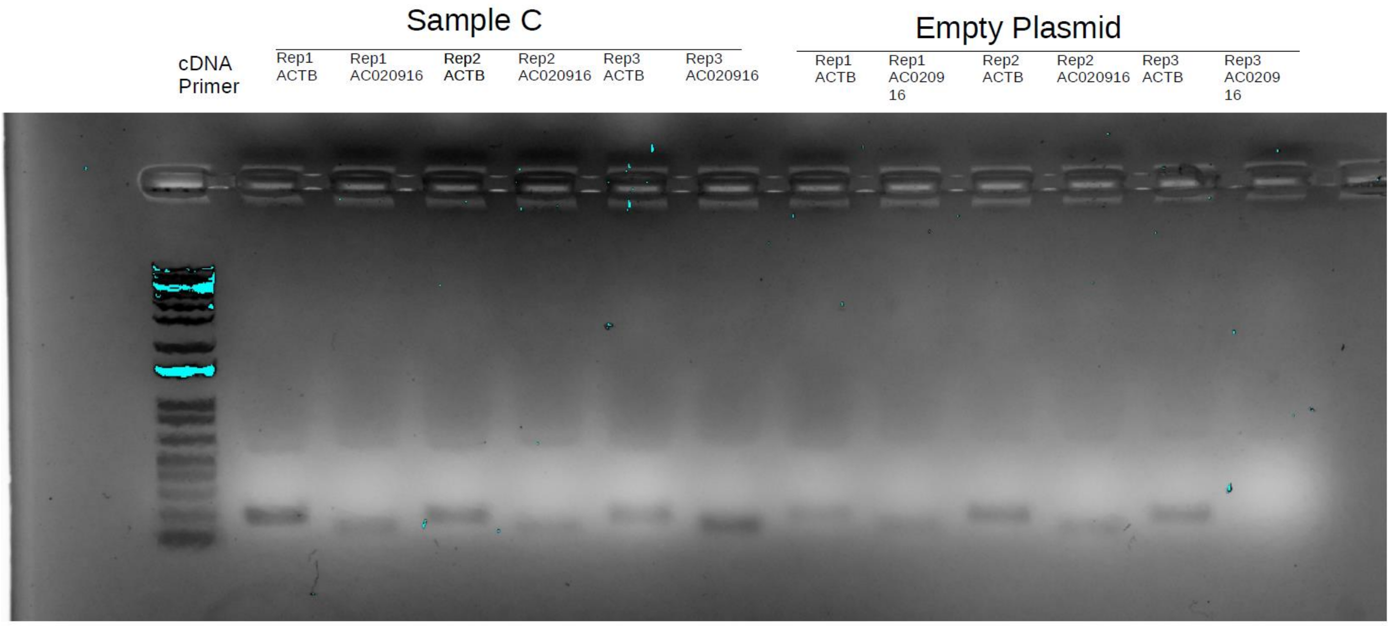

Supplementary Figure S6: PCR results for experiments targeting NOS3.

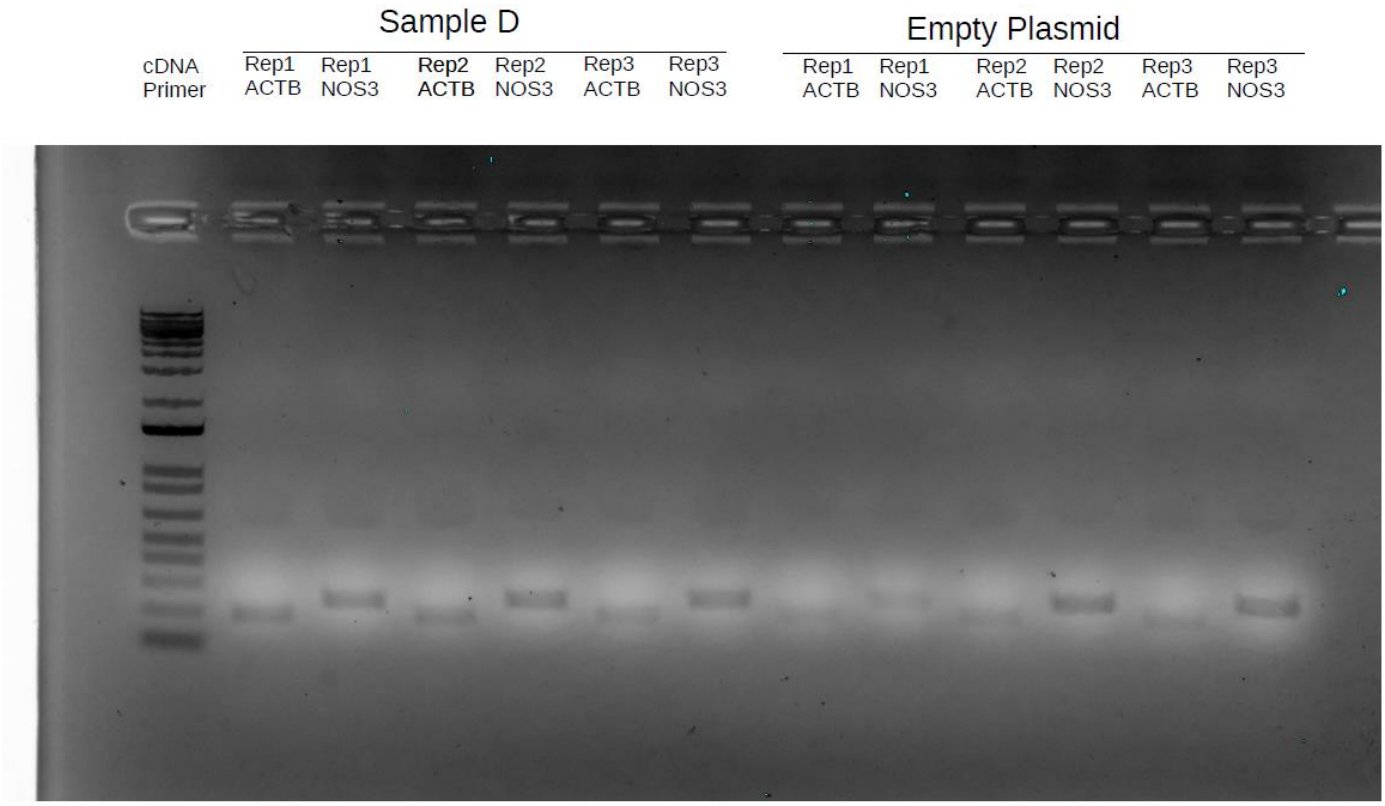

Supplementary Figure S7: PCR results for experiments targeting AC020916.

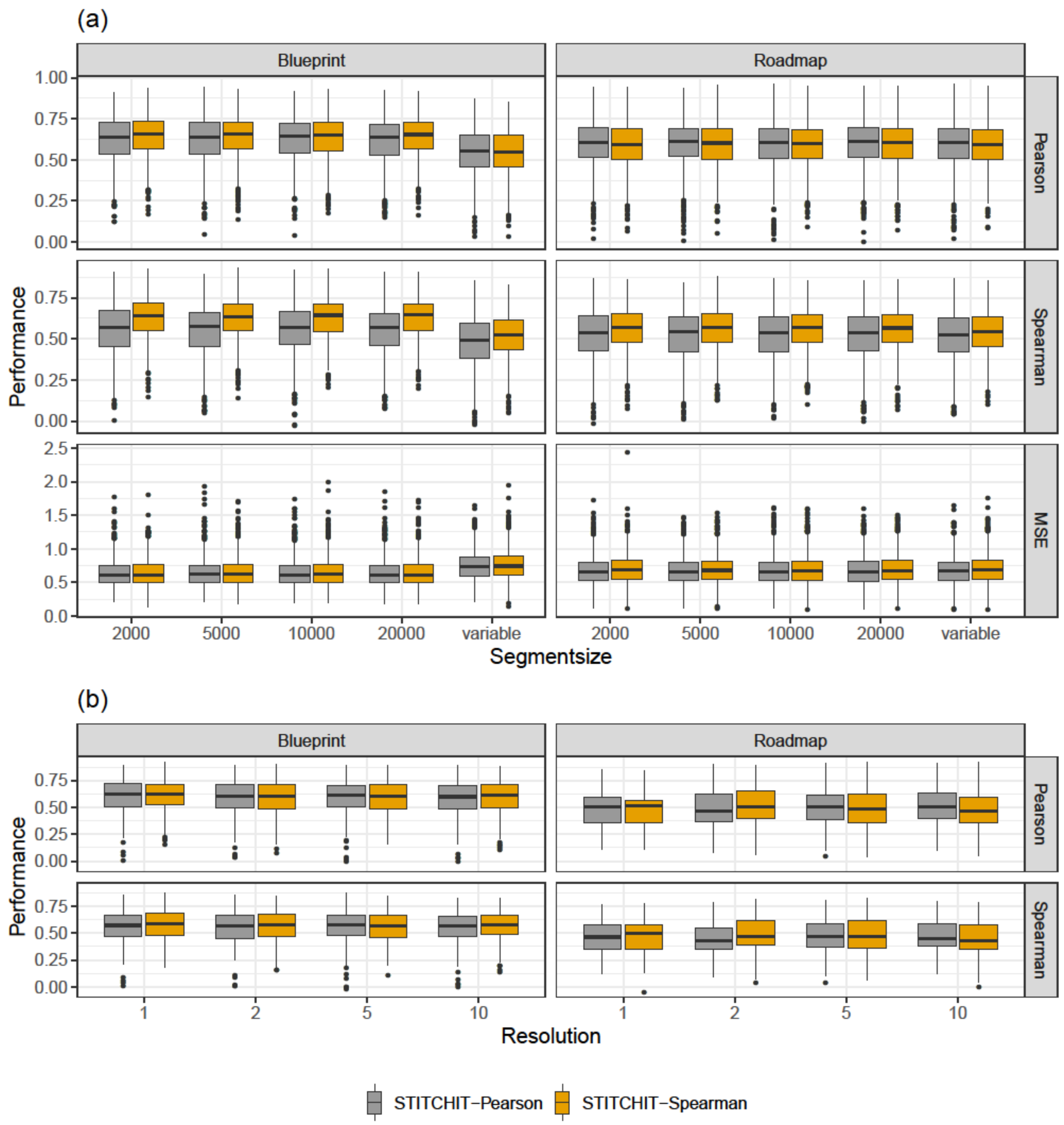

Supplementary Figure S8: (a) Influence of different values for the step size parameter used in STITCHIT on model performance. Importantly we note that a fixed value of the segment size is beneficial in the case of Blueprint data. (b) Influence of different values for the resolution parameter used in STITCHIT on model performance. Increasing the resolution leads to an improved runtime of STITCHIT as the initial binning is smaller.

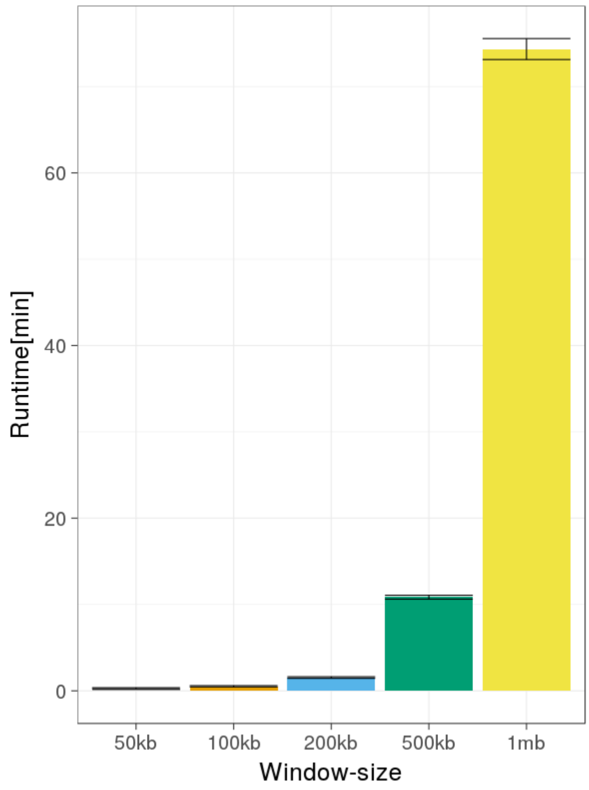

Supplementary Figure S9: Runtime of STITCHIT [min] depending on the used extension up- and downstream of the target gene.

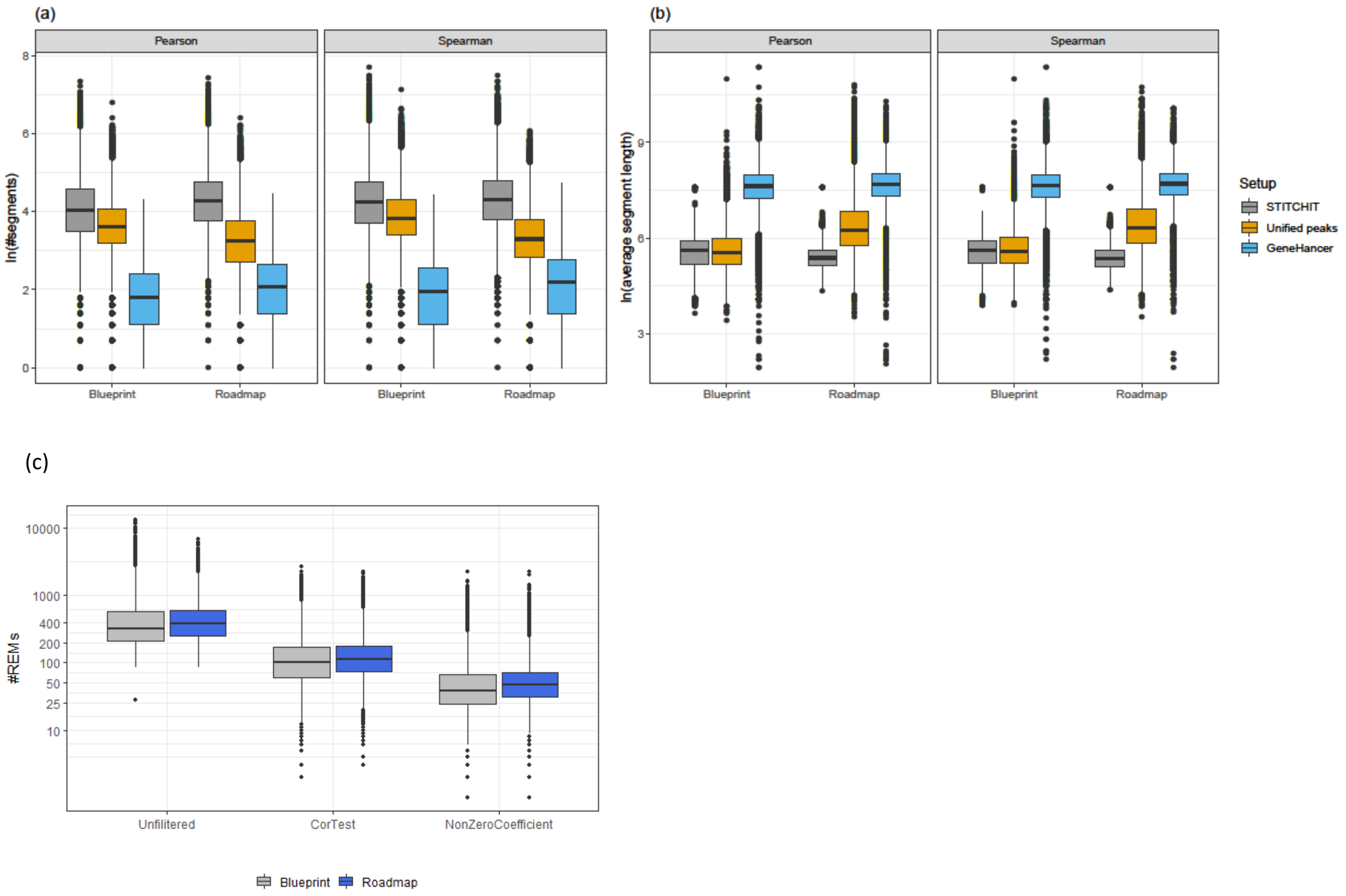

Supplementary Figure S10: (a) Number of segments suggested by STITCHIT, Unified peaks or GeneHancer using either Pearson or Spearman correlation as an initial filtering. (b) Length of segments suggested by STITCHIT, Unified peaks or GeneHancer using either Pearson or Spearman correlation as an initial filtering. (c) Number of REMS for STITCHIT obtained for Blueprint and Roadmap data as Unfiltered REMs, upon the initial correlation based test and after fitting the regression model considering only REMs with a non-zero coefficient across the outer cross validation.

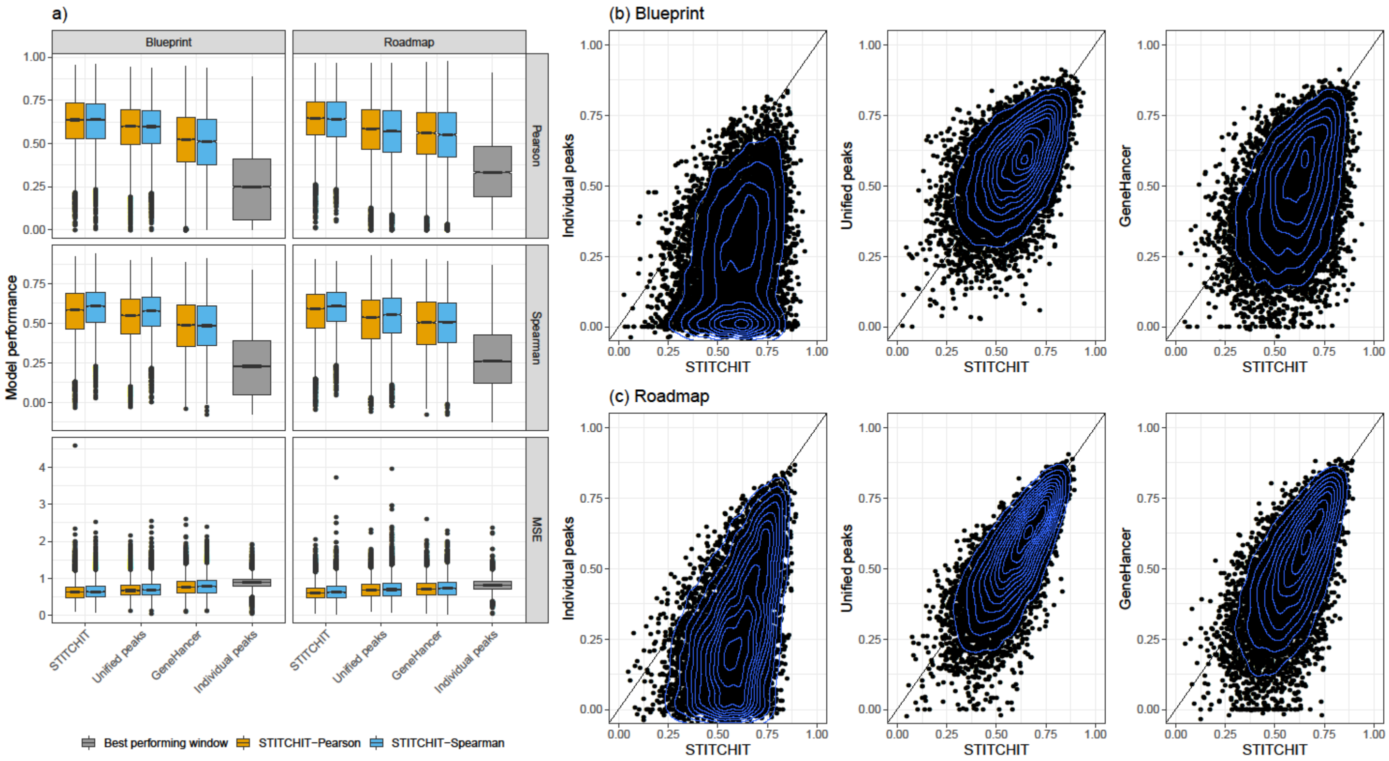

Supplementary Figure S11: (a) Model performance assessed in terms of Pearson and Spearman correlation as well as using the Mean Squared error (MSE) for all consortia. The models are compared on the same gene sets within one consortia. (b-c) Scatter plots comparing the spearman correlation of genes for two distinct models on Blueprint and Roadmap data respectively.

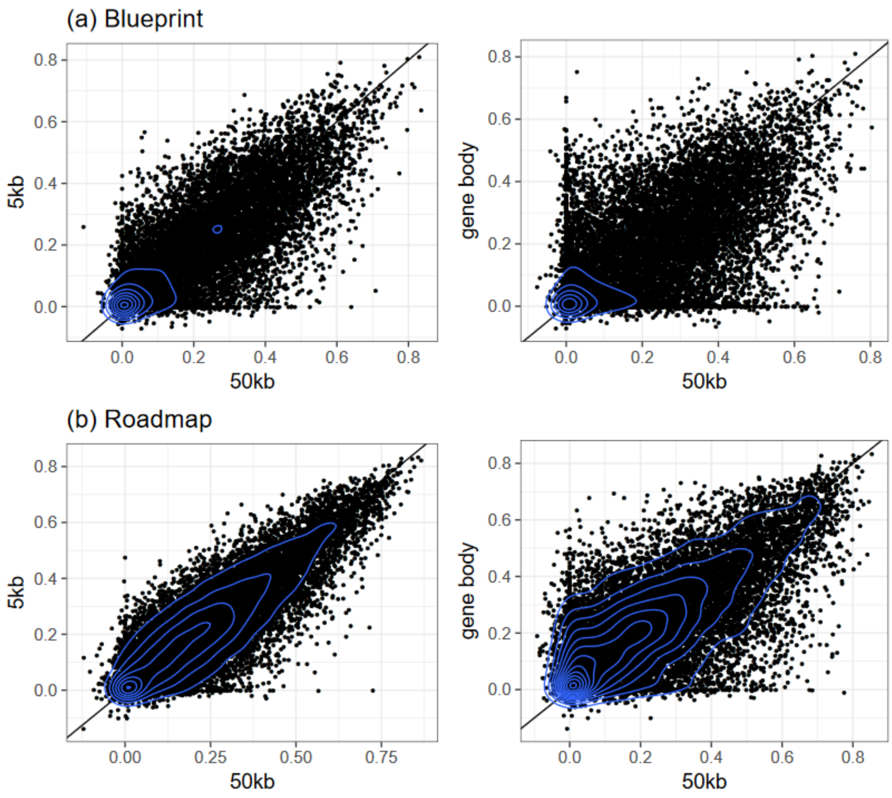

Supplementary Figure S12: Scatterplots comparing different unsupervised annotation approaches (5kb, 50kb, gene body) on Blueprint (a) and Roadmap (b) data.

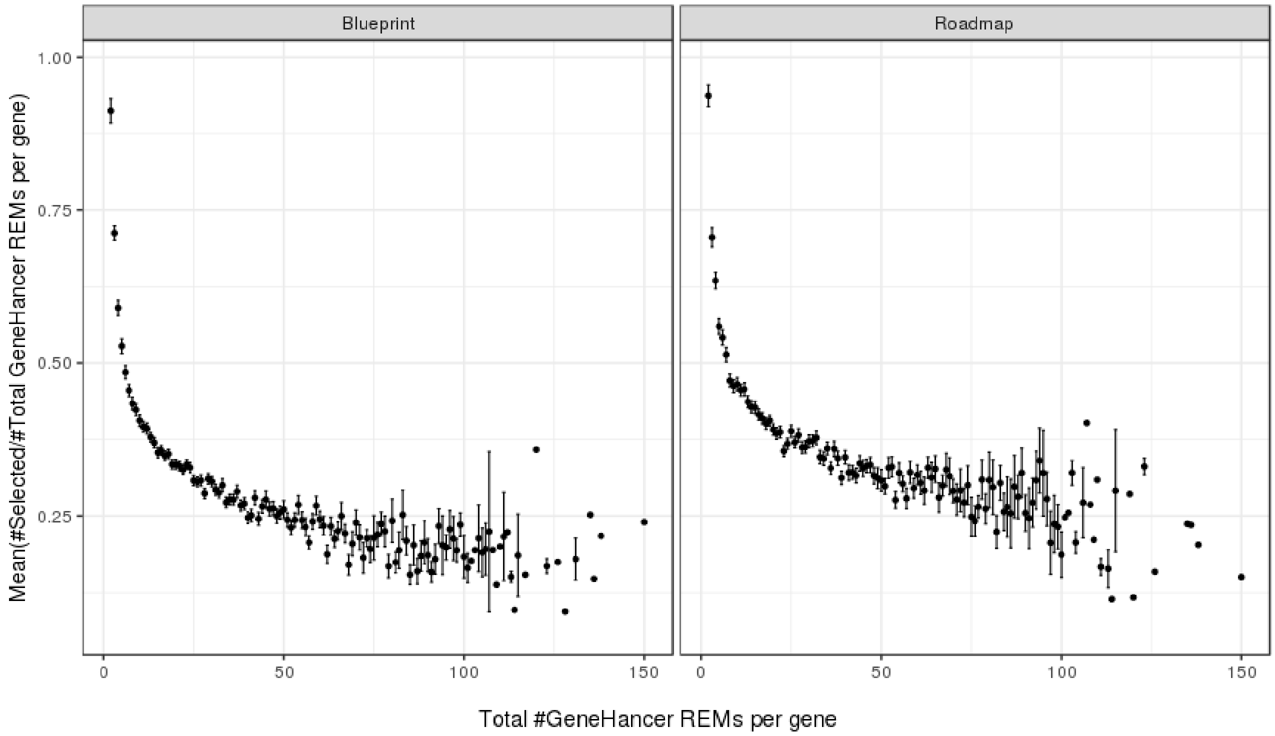

Supplementary Figure S13: Here, the x-axis shows the total number of regulatory elements contained in the GeneHancer database for a gene. The y-axis shows the mean across all between the number of selected enhancers and the total number of known enhancers as indicated on the x-axis. We observe a decreasing trend that tends to stabilize around 0.25.

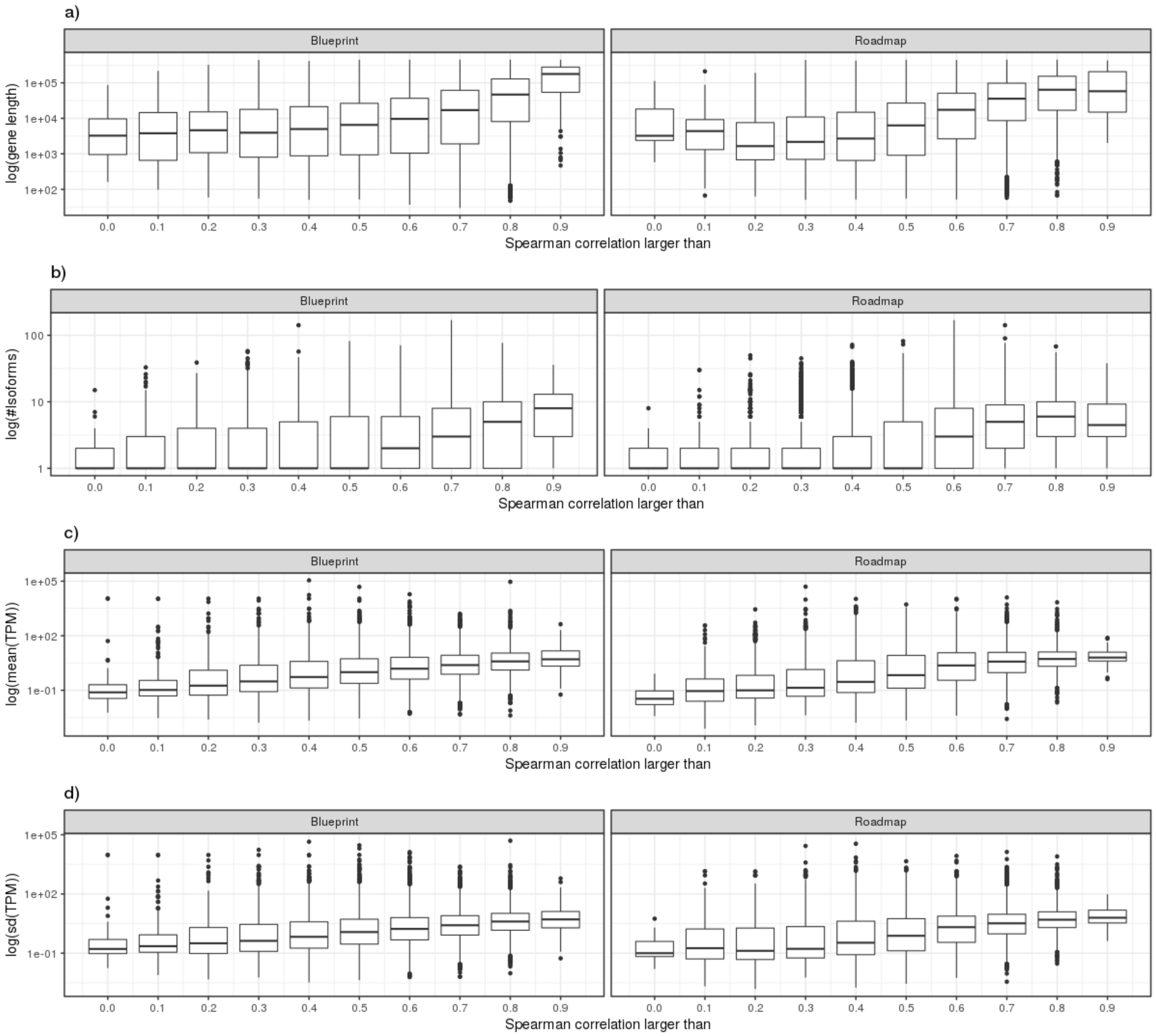

Supplementary Figure S14: Analysis of variables that are linked to the performance of the linear models predicting gene-expression: (a) gene length, (b) number of isoforms, (c) average gene expression in TPM and (d) the standard deviation of gene expression

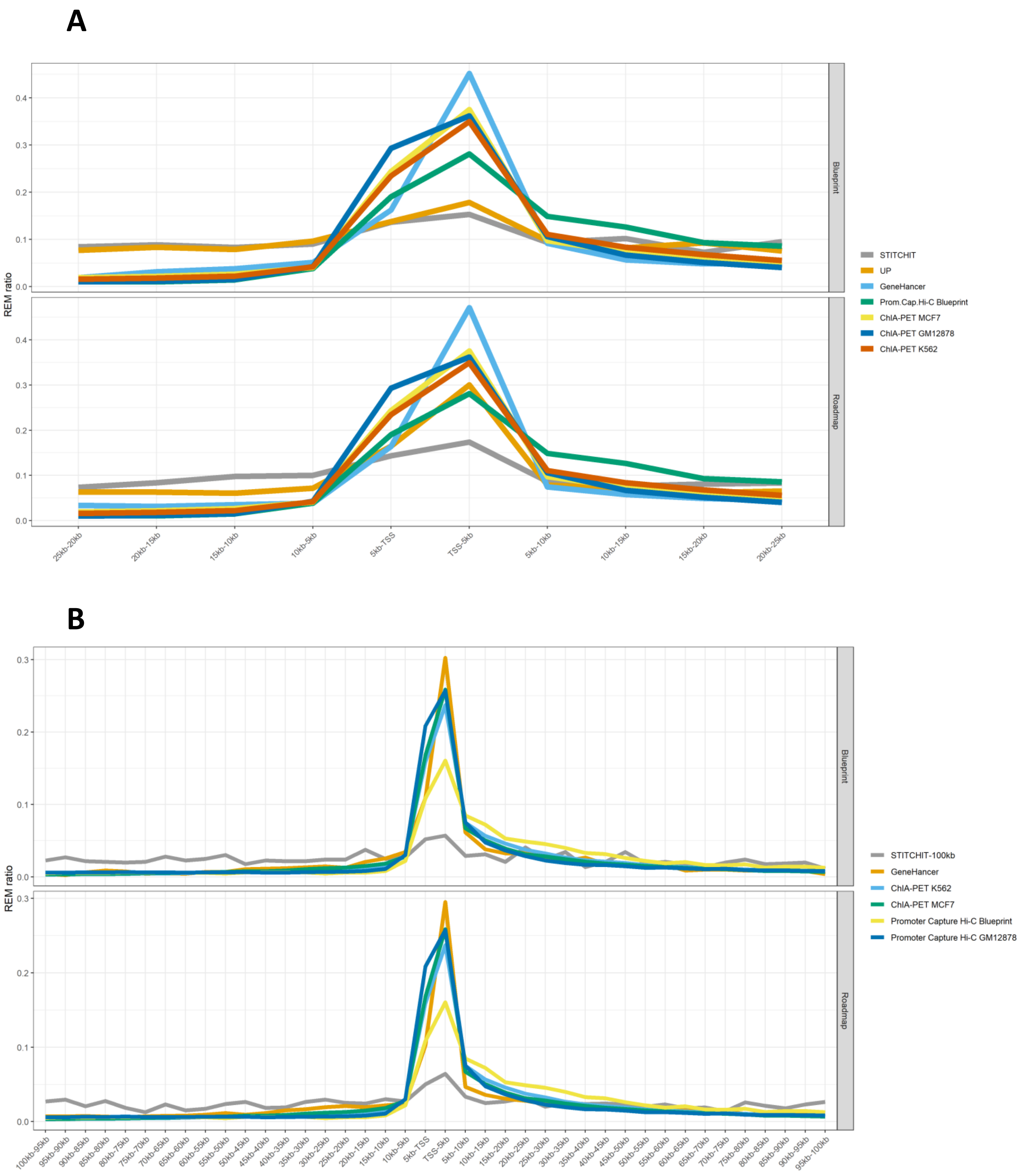

Supplementary Figure S15: **A** Distribution of PEIs around genes for all datasets and all methods including experimentally determined chromatin interactions using ChIA-PET and Promoter Capture Hi-C data for a 25kb window. We observe an enrichment at the TSS (TSS-5kb) and a decline both up- and downstream of the promoter region. **B** Same as A for a 100kb window. REMs with a p-value ≤ 0.05 and absolute regression coefficients ≥ 0.1 are considered.

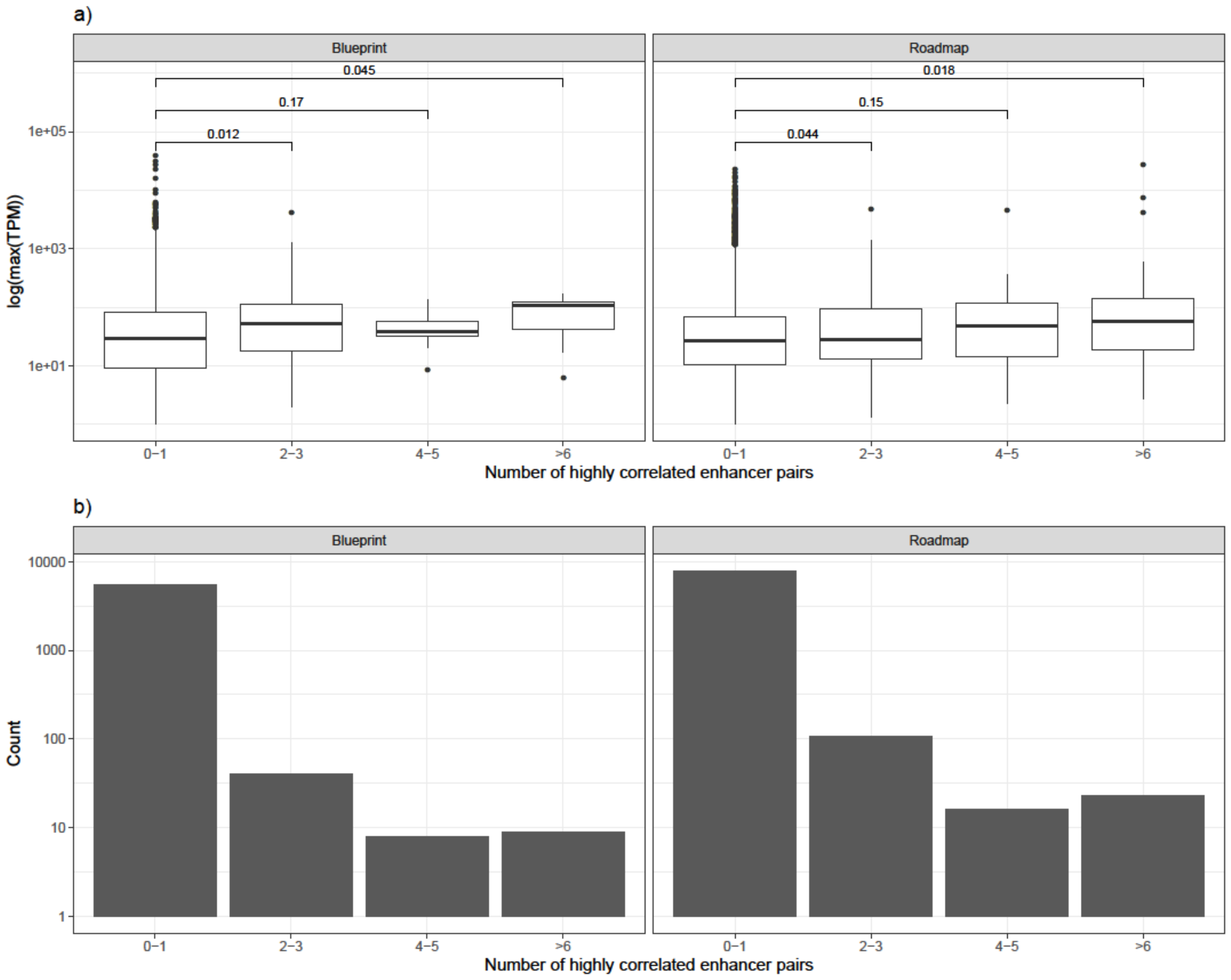

Supplementary Figure S16: Similar to Andersson *et al.* we investigated the presence of additive enhancers in our data set [16]. The maximum expression of genes associated to such additive enhancers' pairs is shown in (a) where a slight trend towards a correlation between the number of correlated enhancer pairs and the maximum gene expression can be observed. As indicated in (b) we only observe a few enhancers that are classified as being additive.

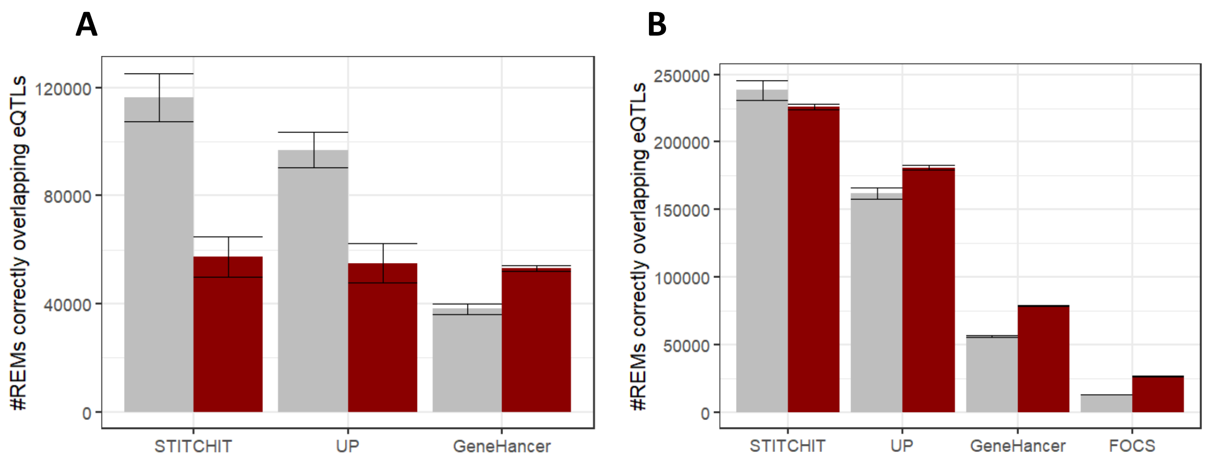

Supplementary Figure S17: Number of unique REMs correctly overlapping with GTEX eQTLs in (A) Blueprint and (B) Roadmap data. Grey bars show the number for the real REM sets and red bars show the numbers for the random REM sets.

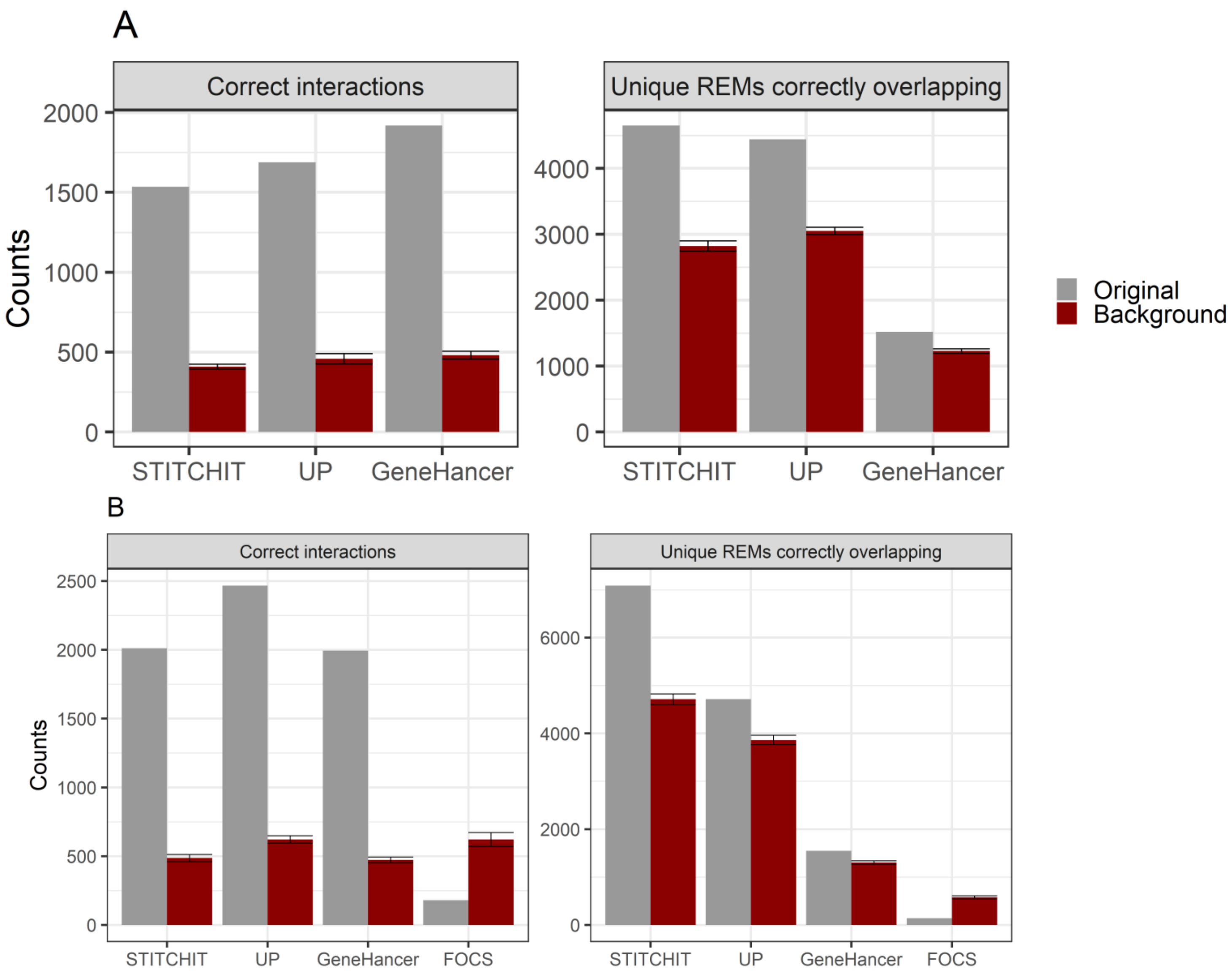

Supplementary Figure S18: Number of unique interactions and unique REMs correctly overlapping with Promoter Capture Hi-C data from Javierre et al. (2016, Cell) for (a) Blueprint and (b) Roadmap data. Grey bars show the number for the real REM sets and red bars show the numbers for the random REM sets.

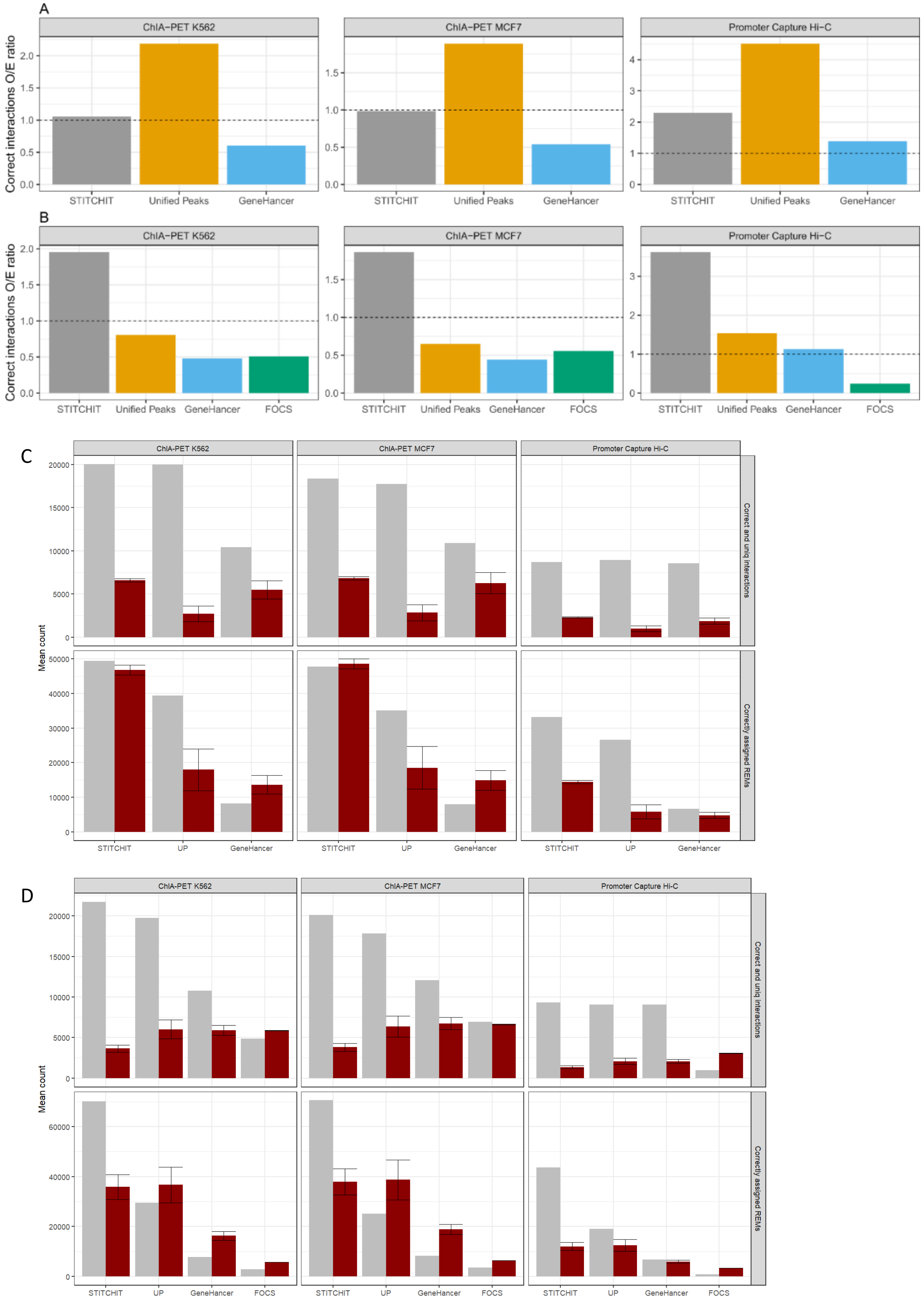

Supplementary Figure S19: OE ratio indicating the number of unique REMs overlapping chromatin interactions in ChIA-PET data in K562 and MCF7 as well as Promoter Capture Hi-C data from GM12878 using (a) Blueprint and (b) Roadmap data. Number of unique interactions and unique REMs correctly overlapping with ChIA-Pet and GM12878 promoter capture Hi-C interactions in Blueprint (c) and Roadmap (d). Grey bars show the number for the real REM sets and red bars show the numbers for the random REM sets.

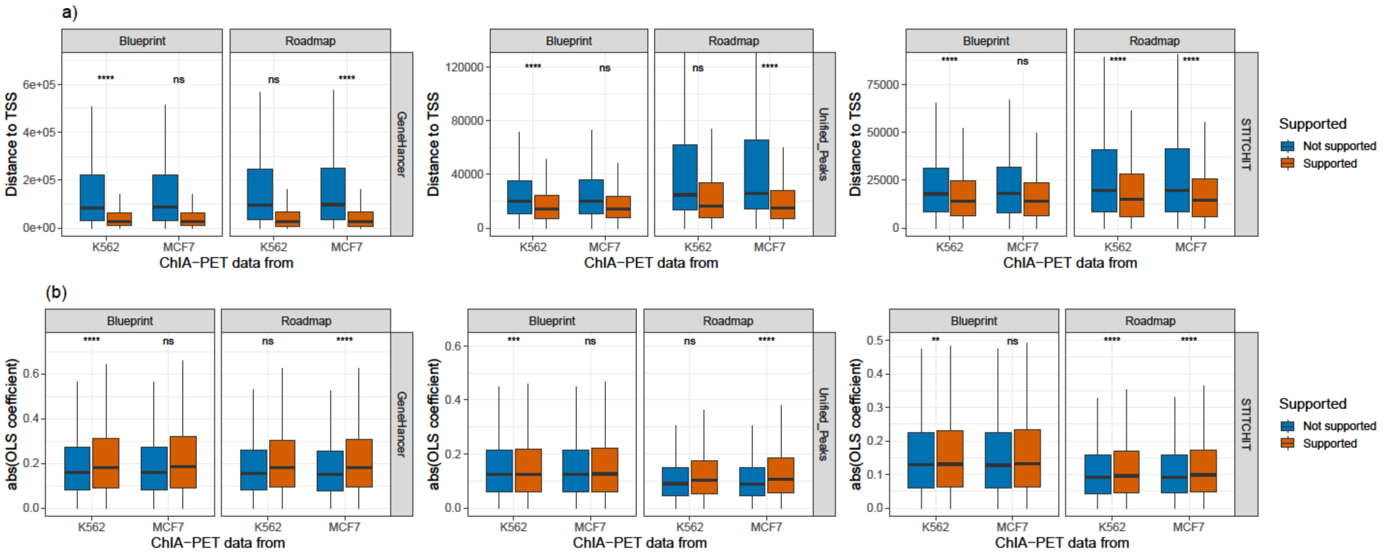

Supplementary Figure S20: a) The distance of a REM to the TSS of their target genes is contrasted for REMs unsupported and supported REMs with respect to ChIA-PET data. b) Instead of distance to TSS, REMs are contrasted with respect to their absolute OLS coefficient.

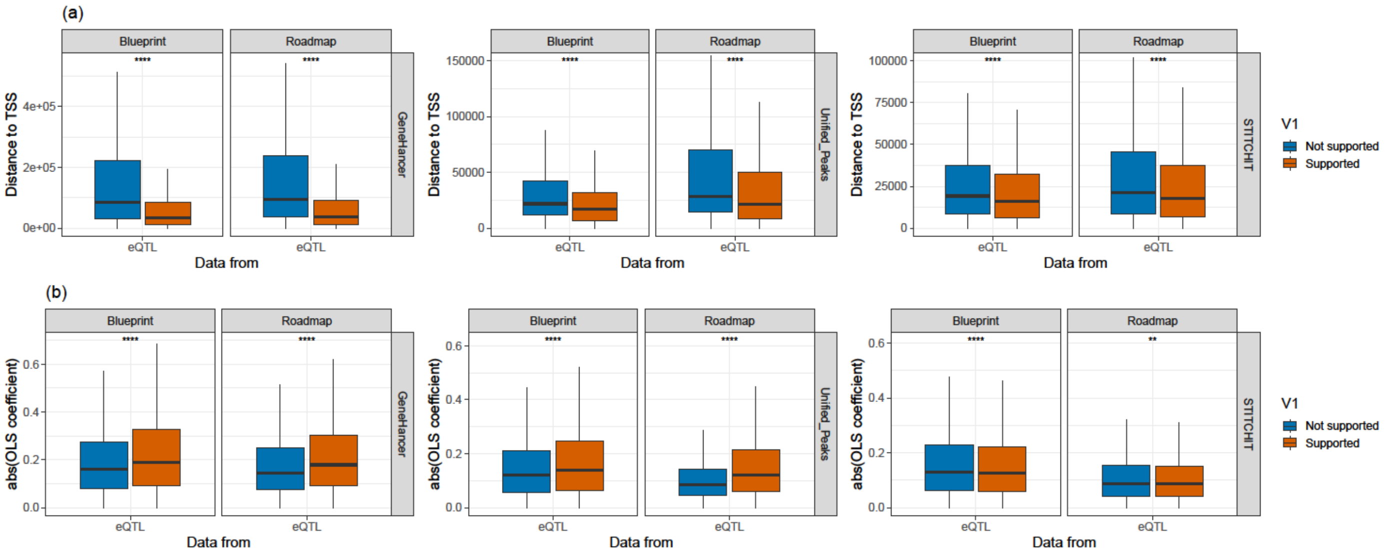

Supplementary Figure S21: a) The distance of a REM to the TSS of their target genes is contrasted for REMs unsupported and supported REMs with respect to eQTLs from the ExSNP database. b) Instead of distance to TSS, REMs are contrasted with respect to their absolute OLS coefficient (NS: not significant, *: p < 0.05, **: p < 0.01, ***: p < 0.001, ****: p< 0.0001).

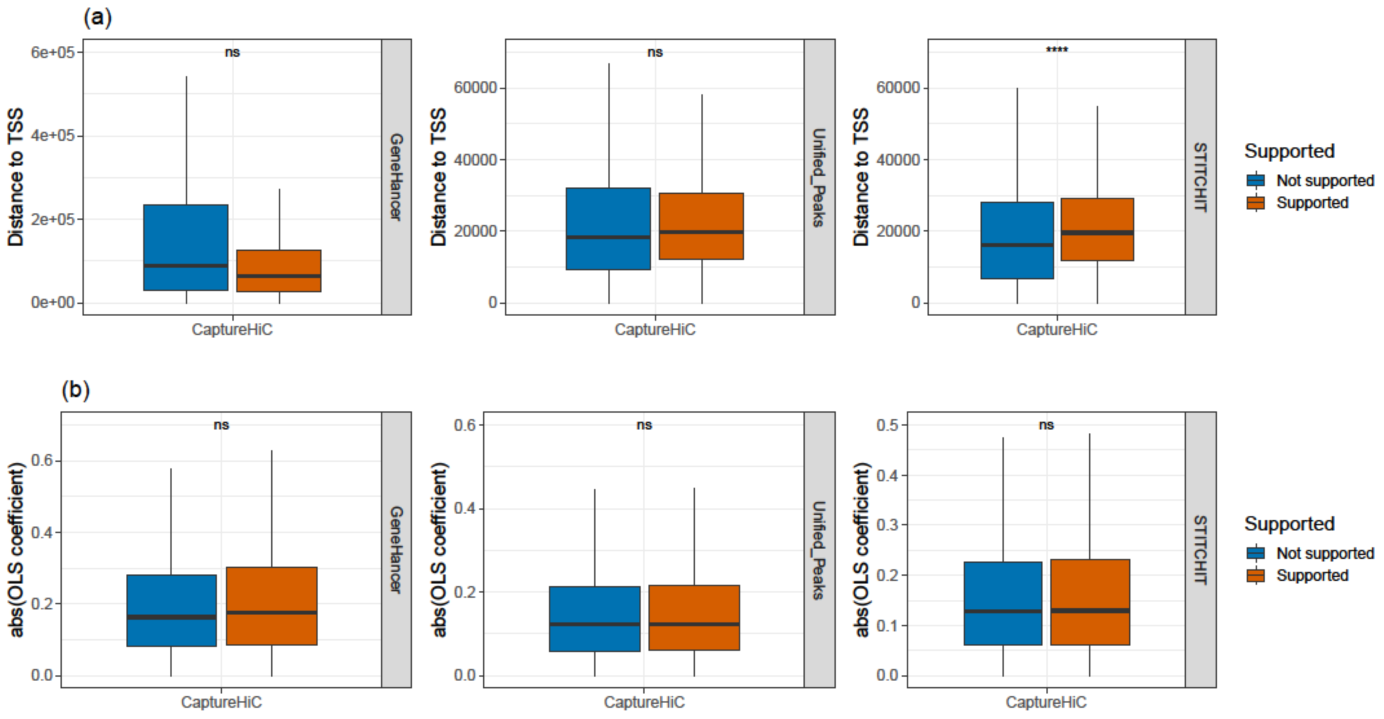

Supplementary Figure S22: a) The distance of a REM to the TSS of their target genes is contrasted for REMs unsupported and supported REMs with respect to GM12878 Capture Hi-C data using the Blueprint data set. b) Instead of distance to TSS, REMs are contrasted with respect to their absolute OLS coefficient (NS: not significant, *: $p < 0.05$, **: $p < 0.01$, ***: $p < 0.001$, ****: $p < 0.0001$).

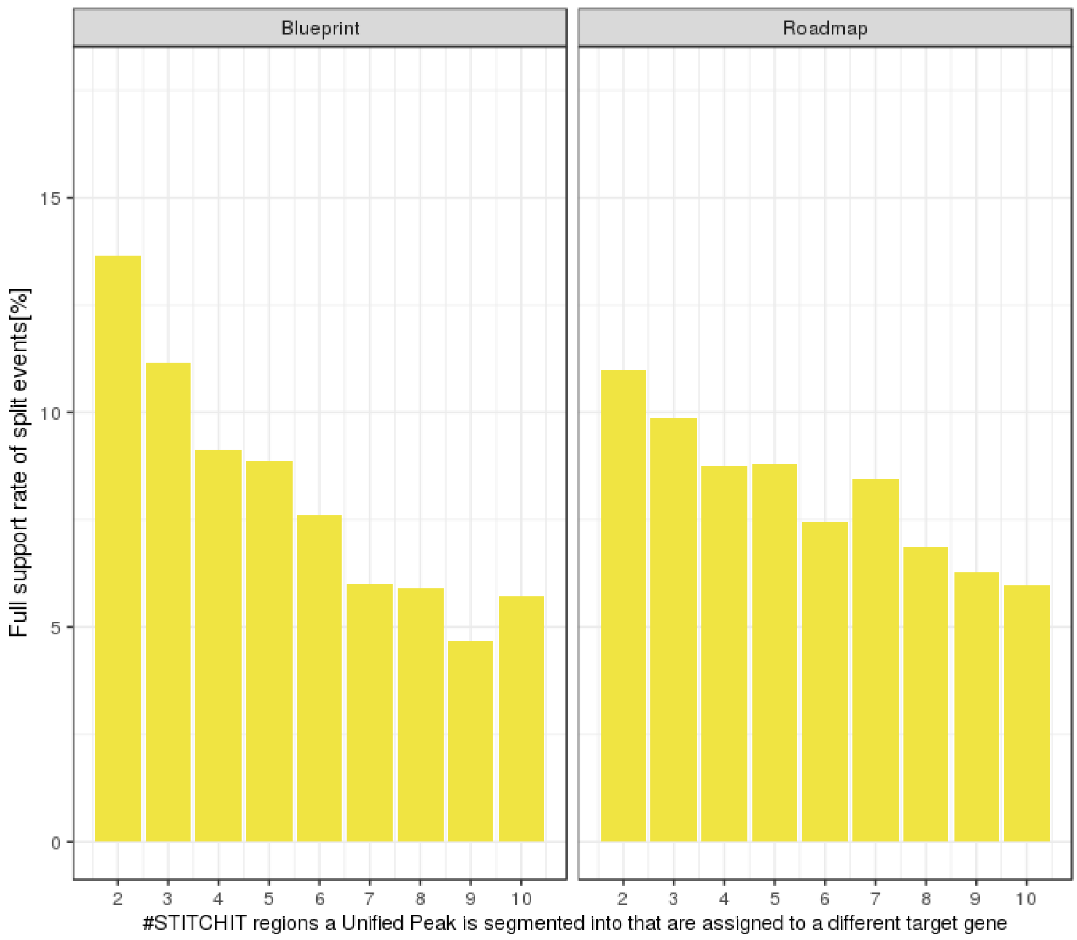

Supplementary Figure S23: Full support rate through ChIA-PET data for split events shown separately for Blueprint and Roadmap data sets.

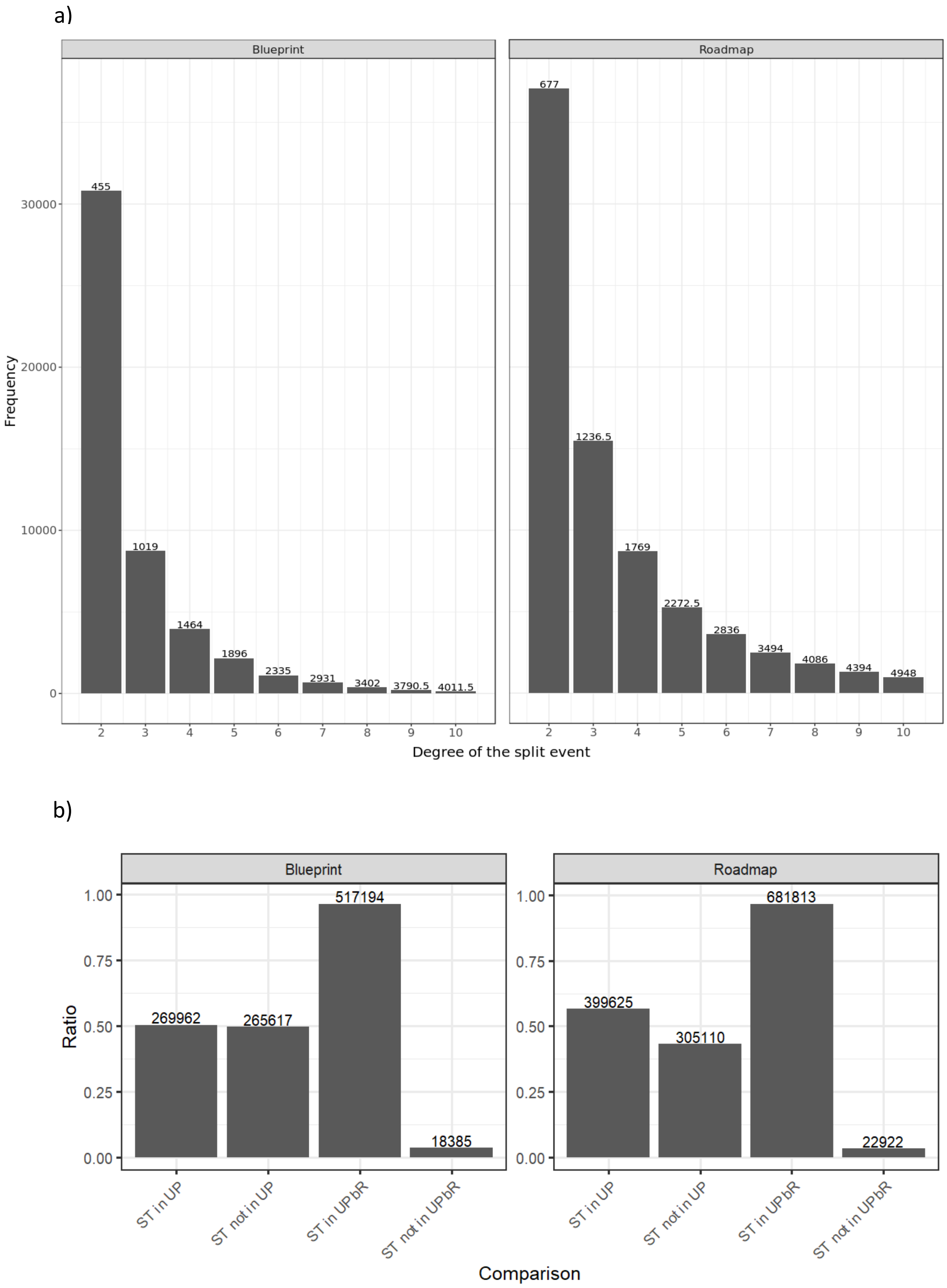

Supplementary Figure S24: a) Frequency of Unified Peak (UP) splits into various STITCHIT REMs, which are linked to the same gene, are shown per data set for various degrees of split events. The median length of the splitted UP is shown on top of the bars. As shown, the length of the UP increases with increasing degree of the split events. b) Overlap of STITCHIT REMs with UP before (UP bR) and after (UP) regression based filtering for Blueprint and Roadmap.

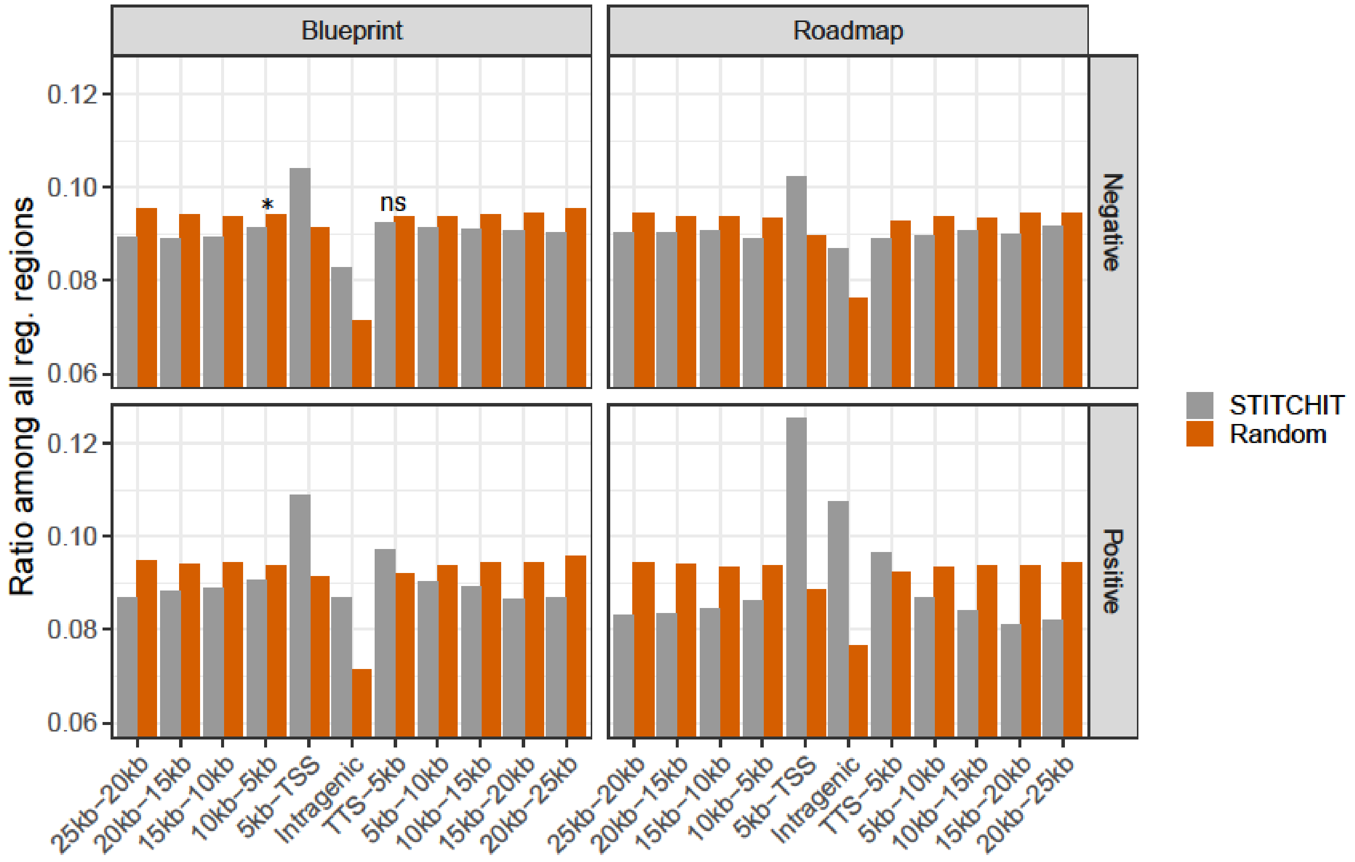

Supplementary Figure S25: Distribution of REMs around a gene contrasted against a random model shown separately for REMs with a negative and a positive coefficient. Compared to the random model, REMs with a positive reg. coefficient are enriched at the gene promoter, are depleted in the gene body and enriched directly downstream of the Transcription Termination Site (TTS). They tend to be depleted further away from the gene. REMs with a negative ref. coefficient are enriched at the TSS, depleted in the gene body and, unlike positive REMs, also depleted directly downstream of the TTS. Significance was assessed using a one-sample t-test. Each comparison obtained a BH corrected p-value < 0.001 if not indicated otherwise (NS: not significant, *: p-adjust < 0.05).

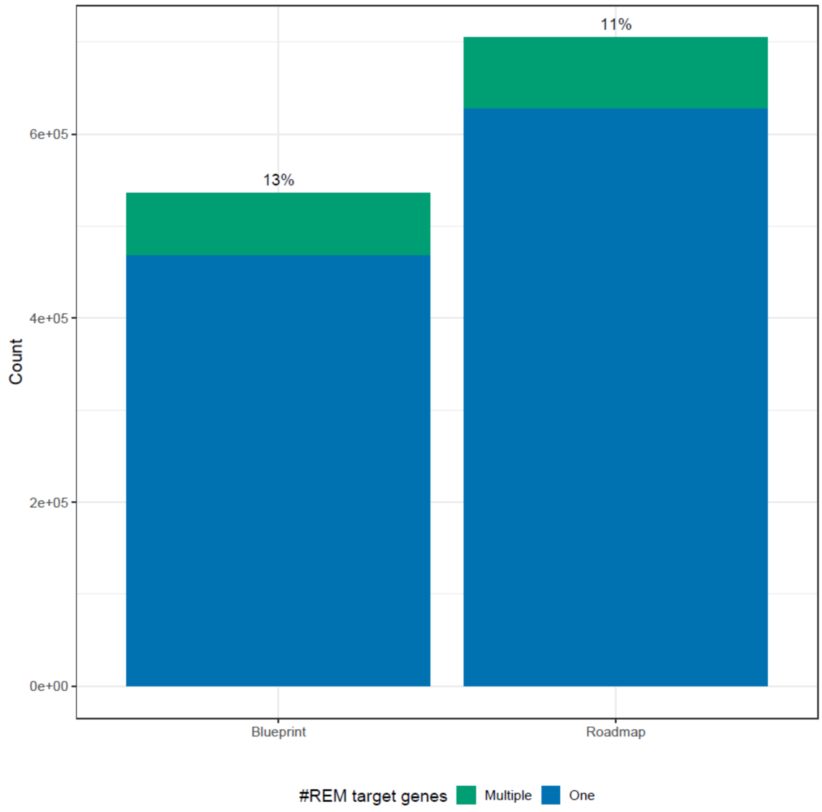

Supplementary Figure S26: Here, we report how many REMs target one or more than one gene. To perform this comparison, a set of union REMs has been generated. The label above the boxes indicates the percentage of REMs with more than one target gene.

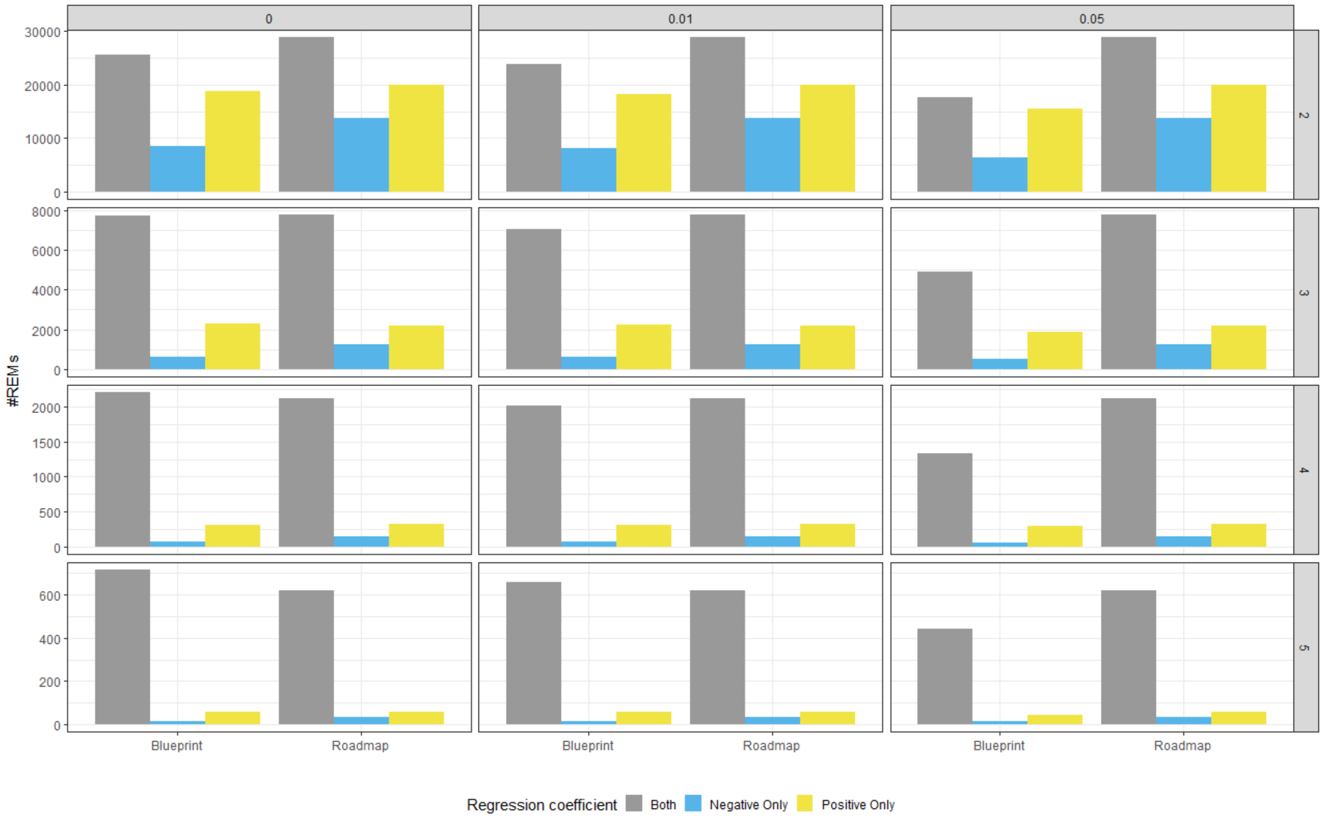

Supplementary Figure S27: Illustration of the distribution of the number of REMs targeting more than one gene as predicted by STITCHIT elucidating the number of REMs with an exclusively positive, exclusively negative or with both positive and negative regression coefficient signs. The columns of the facet indicate a threshold on the absolute value of the reg. coefficient, the rows indicate the number of target genes. The trend that *Both > Positive Only > Negative Only*, is consistent for all experiments.

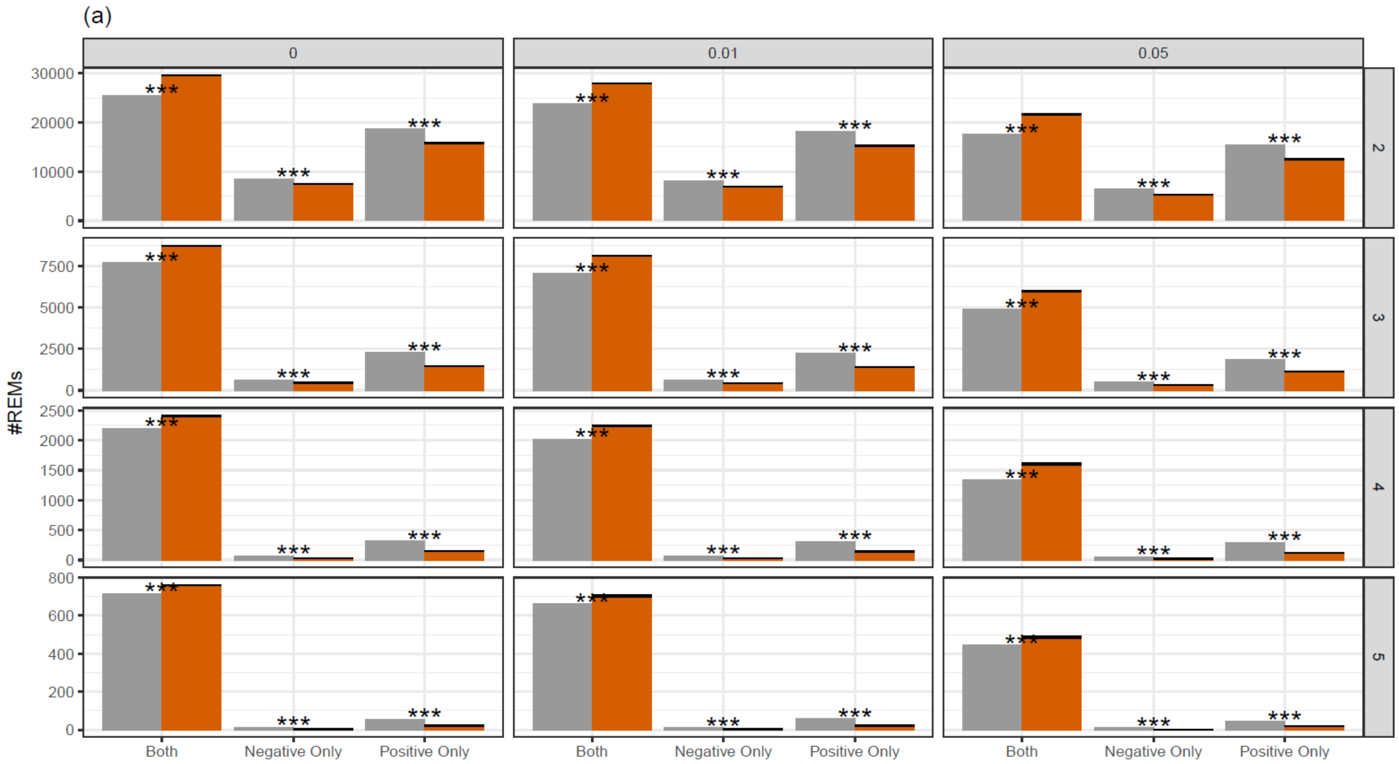

Supplementary Figure S28a: Comparison of REM separating REMs into exclusively negative, positive or both interactions against a random background model for Blueprint data. The columns of the facet indicate a threshold on the absolute value of the reg. coefficient, the rows indicate the number of target genes. Throughout all facets, we observe less REMs occurring in the *Both* group than expected, whereas we observe more in the negative and positive only groups, respectively. Significance was assessed using a one-sample t-test (NS: not significant, *: p < 0.05, **: p < 0.01, ***: p < 0.001, ****: p< 0.0001).

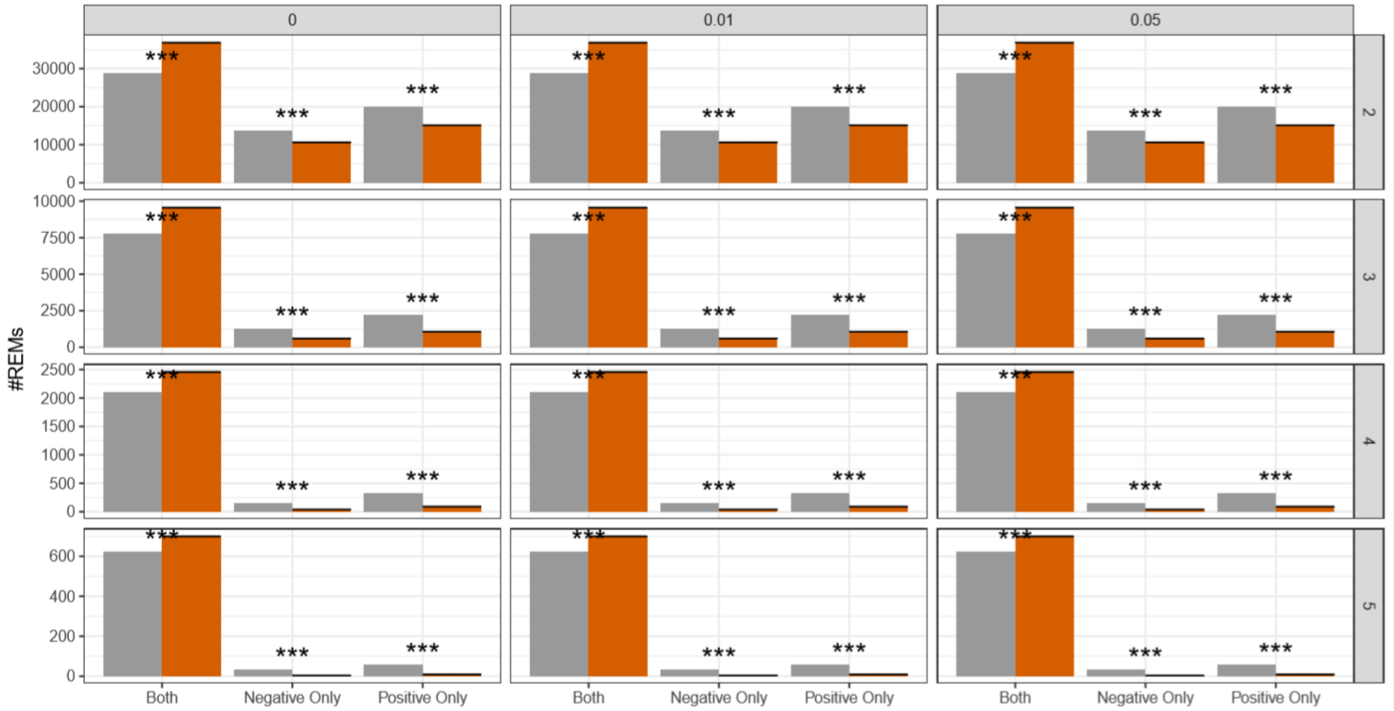

Supplementary Figure S28b: Comparison of REM separating REMs into exclusively negative, positive or both interactions against a random background model for Roadmap data. The columns of the facet indicate a threshold on the absolute value of the reg. coefficient, the rows indicate the number of target genes. Throughout all facets, we observe less REMs occurring in the *Both* group than expected, whereas we observe more in the negative and positive only groups, respectively. Significance was assessed using a one-sample t-test (NS: not significant, *: p < 0.05, **: p < 0.01, ***: p < 0.001, ****: p< 0.0001).).

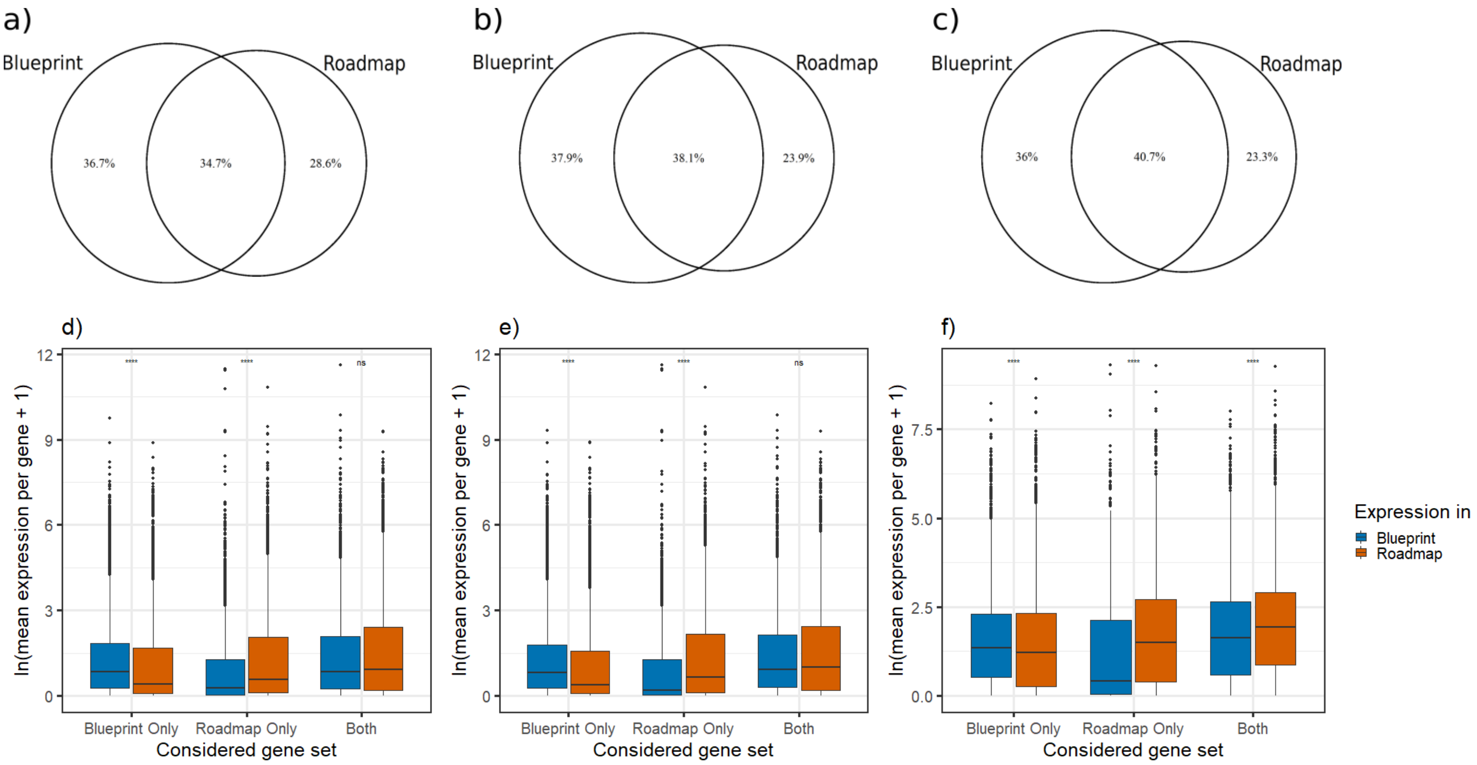

Supplementary Figure S29: Venn Diagramms indicating the overlap in terms of covered genes for a) STITCHIT, b) Unified Peaks, and c) GeneHancer data respectively. In d-f, the expression of the genes shown in a-c is depicted for d) STTICHIT, e) Unified Peaks and f) GeneHancer, respectively.

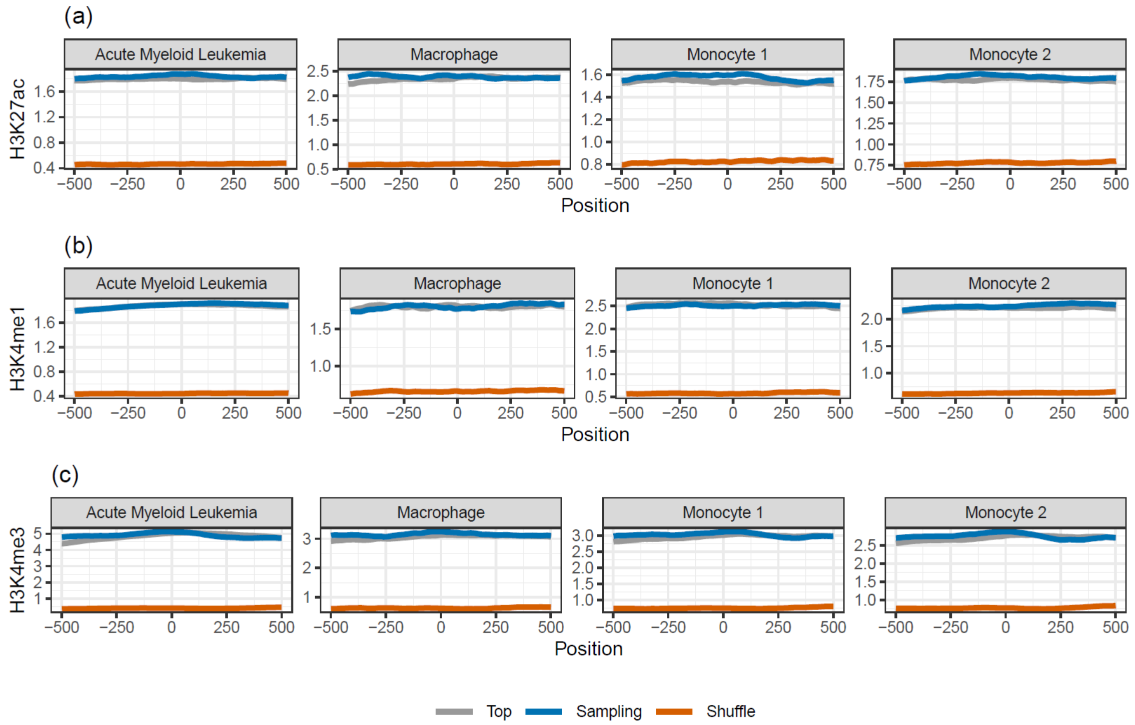

Supplementary Figure S30: Enrichment of (a) H3K27ac, (b) H3K4me1, and (c) H3K4me3 in the top 10.000 STITCHIT REMs, ranked by OLS p-Value (grey), 10.000 randomly sampled STITCHIT REMs (blue) and 10.000 randomly placed genomic intervals (orange). STITCHIT REMs are enriched for the tested HM signatures, which are characteristic for both active promoters and enhancer regions.

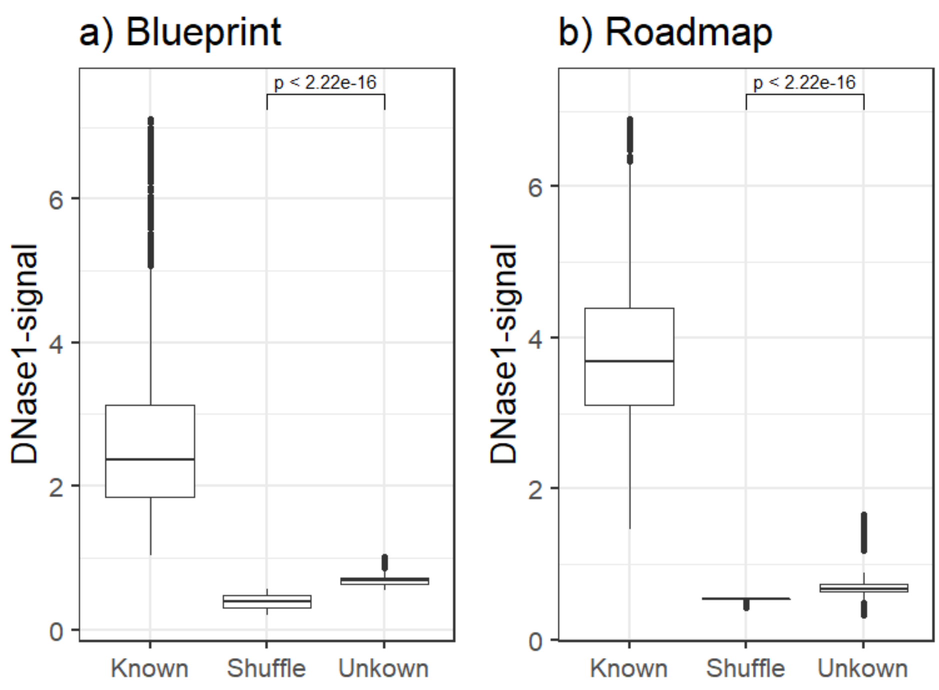

Supplementary Figure S31: Boxplots contrasting the DNase1 signal within Known, Shuffled and Unknown STITCHIT REMs for a) Blueprint and b) Roadmap.

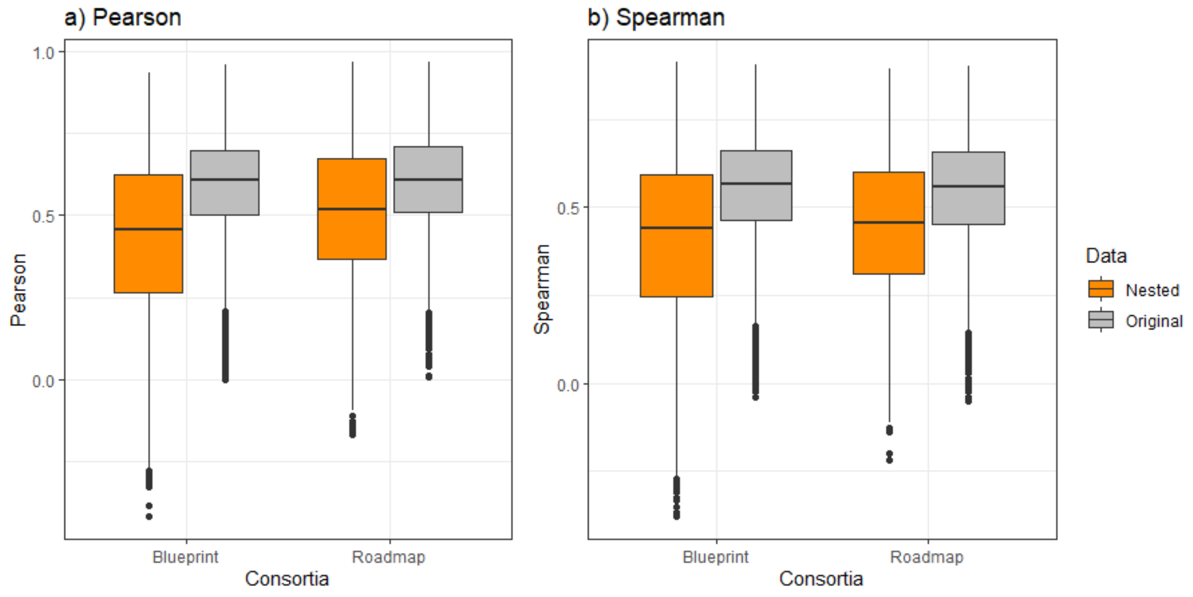

Supplementary Figure S32: Mean a) Pearson and b) Spearman correlation across a 10-fold Monte Carlo cross-validation assessed for individual genes on Blueprint and Roadmap data using nested feature selection compared to the original ML pipeline.

Consortium	File ID	Tissue/Cell type	Data type	Internal Sample ID
Blueprint	130919_SN546_0217_B_C2GFPACXX_CTTGTA_1_read2.fastq.gz	CD8-positive, alpha-beta T cell	RNA-seq	B_C0066P12
Blueprint	130919_SN546_0217_B_C2GFPACXX_CTTGTA_1_read1.fastq.gz	CD8-positive, alpha-beta T cell	RNA-seq	B_C0066P12
Blueprint	130207_SN546_0194_A_H093AADXX_CTTGTA_1_read1.fastq.gz	CD14-positive, CD16-negative classical monocyte	RNA-seq	B_C005PS12
Blueprint	130207_SN546_0194_A_H093AADXX_CTTGTA_1_read2.fastq.gz	CD14-positive, CD16-negative classical monocyte	RNA-seq	B_C005PS12
Blueprint	150226_SN935_0006_B_C5YBTACXX_TTAGGC_4_read1.fastq.gz	Acute Lymphocytic Leukemia	RNA-seq	B_S00DFM11
Blueprint	150226_SN935_0006_B_C5YBTACXX_TTAGGC_4_read2.fastq.gz	Acute Lymphocytic Leukemia	RNA-seq	B_S00DFM11
Blueprint	C3JYVACXX_lane6_5385_ACAGTG_L006_R1.fastq.gz	macrophage - T=6days LPS	RNA-seq	B_S00HSH11
Blueprint	C3JYVACXX_lane6_5389_ATCACG_L006_R1.fastq.gz	macrophage - T=6days LPS	RNA-seq	B_S00JRB11
Blueprint	C2MVTACXX_lane7_4641_ACAGTG_L007_R1.fastq.gz	macrophage - T=6days LPS	RNA-seq	B_S00BYT11
Blueprint	C2NTEACXX_lane7_4819_ACAGTG_L007_R1.fastq.gz	macrophage - T=6days LPS	RNA-seq	B_S00CS011
Blueprint	C250RACXX_lane5_3990_CTTGTA_L005_R2.fastq.gz	CD34-negative, CD41-positive, CD42-positive megakaryocyte cell	RNA-seq	B_C006NSB1
Blueprint	C250RACXX_lane5_3990_CTTGTA_L005_R1.fastq.gz	CD34-negative, CD41-positive, CD42-positive megakaryocyte cell	RNA-seq	B_C006NSB1
Blueprint	C250RACXX_lane7_3990_CTTGTA_L007_R2.fastq.gz	CD34-negative, CD41-positive, CD42-positive megakaryocyte cell	RNA-seq	B_C006NSB1
Blueprint	C250RACXX_lane7_3990_CTTGTA_L007_R1.fastq.gz	CD34-negative, CD41-positive, CD42-positive megakaryocyte cell	RNA-seq	B_C006NSB1
Blueprint	C250RACXX_lane8_3990_CTTGTA_L008_R1.fastq.gz	CD34-negative, CD41-positive, CD42-positive megakaryocyte cell	RNA-seq	B_C006NSB1
Blueprint	C250RACXX_lane8_3990_CTTGTA_L008_R2.fastq.gz	CD34-negative, CD41-positive, CD42-positive megakaryocyte cell	RNA-seq	B_C006NSB1
Blueprint	C250RACXX_lane6_3990_CTTGTA_L006_R2.fastq.gz	CD34-negative, CD41-positive, CD42-positive megakaryocyte cell	RNA-seq	B_C006NSB1
Blueprint	C250RACXX_lane6_3990_CTTGTA_L006_R1.fastq.gz	CD34-negative, CD41-positive, CD42-positive megakaryocyte cell	RNA-seq	B_C006NSB1
Blueprint	130919_SN546_0217_B_C2GFPACXX_ACAGTG_2_read1.fastq.gz	CD34-negative, CD41-positive, CD42-positive megakaryocyte cell	RNA-seq	B_S004BT
Blueprint	130919_SN546_0217_B_C2GFPACXX_ACAGTG_2_read2.fastq.gz	CD34-negative, CD41-positive, CD42-positive megakaryocyte cell	RNA-seq	B_S004BT
Blueprint	140618_SN935_0195_A_C42K0ACXX_ACTGAT_3_read1.fastq.gz	CD4-positive, alpha-beta T cell	RNA-seq	B_S008H111
Blueprint	140618_SN935_0195_A_C42K0ACXX_ACTGAT_3_read2.fastq.gz	CD4-positive, alpha-beta T cell	RNA-seq	B_S008H111
Blueprint	140618_SN935_0195_A_C42K0ACXX_GCCAAT_1_read1.fastq.gz	erythroblast	RNA-seq	B_S002R512
Blueprint	140618_SN935_0195_A_C42K0ACXX_GCCAAT_1_read2.fastq.gz	erythroblast	RNA-seq	B_S002R512
Blueprint	130919_SN546_0217_B_C2GFPACXX_GTGAAA_2_read2.fastq.gz	erythroblast	RNA-seq	B_S002S312
Blueprint	130919_SN546_0217_B_C2GFPACXX_GTGAAA_2_read1.fastq.gz	erythroblast	RNA-seq	B_S002S312
Blueprint	130919_SN546_0217_B_C2GFPACXX_TTAGGC_5_read1.fastq.gz	macrophage	RNA-seq	B_S001S714
Blueprint	130919_SN546_0217_B_C2GFPACXX_TTAGGC_5_read2.fastq.gz	macrophage	RNA-seq	B_S001S714
Blueprint	130919_SN546_0217_B_C2GFPACXX_ATCACG_4_read2.fastq.gz	inflammatory macrophage	RNA-seq	B_S001MJ12
Blueprint	130919_SN546_0217_B_C2GFPACXX_ATCACG_4_read1.fastq.gz	inflammatory macrophage	RNA-seq	B_S001MJ12
Blueprint	130919_SN546_0217_B_C2GFPACXX_TGACCA_6_read1.fastq.gz	inflammatory macrophage	RNA-seq	B_S0022I14
Blueprint	130919_SN546_0217_B_C2GFPACXX_TGACCA_6_read2.fastq.gz	inflammatory macrophage	RNA-seq	B_S0022I14
Blueprint	C3JYVACXX_lane6_5384_TGACCA_L006_R1.fastq.gz	macrophage - T=6days untreated	RNA-seq	B_S00HRJ11
Blueprint	C2MVTACXX_lane7_4640_TGACCA_L007_R1.fastq.gz	macrophage - T=6days untreated	RNA-seq	B_S00BXV11
Blueprint	C2NTEACXX_lane7_4818_TGACCA_L007_R1.fastq.gz	macrophage - T=6days untreated	RNA-seq	B_S00CR211
Blueprint	C3JYVACXX_lane6_5388_CTTGTA_L006_R1.fastq.gz	macrophage - T=6days untreated	RNA-seq	B_S00JQD11
Blueprint	150429_SN935_0015_A_C5RHEACXX_GATCAG_1_read2.fastq.gz	Acute Myeloid Leukemia	RNA-seq	B_S013M311
Blueprint	150429_SN935_0015_A_C5RHEACXX_GATCAG_1_read1.fastq.gz	Acute Myeloid Leukemia	RNA-seq	B_S013M311
Blueprint	150429_SN935_0015_A_C5RHEACXX_TTAGGC_1_read1.fastq.gz	Acute Myeloid Leukemia	RNA-seq	B_S00D0F11
Blueprint	150429_SN935_0015_A_C5RHEACXX_TTAGGC_1_read2.fastq.gz	Acute Myeloid Leukemia	RNA-seq	B_S00D0F11
Blueprint	151209_SN546_0285_A_H3WLNADXX_ACAGTG_1_read1.fastq.gz	Acute Myeloid Leukemia	RNA-seq	B_S00D6311
Blueprint	151209_SN546_0285_A_H3WLNADXX_ACAGTG_1_read2.fastq.gz	Acute Myeloid Leukemia	RNA-seq	B_S00D6311
Blueprint	150429_SN935_0015_A_C5RHEACXX_GTCCGC_5_read2.fastq.gz	Acute Myeloid Leukemia	RNA-seq	B_S005EJ11
Blueprint	150429_SN935_0015_A_C5RHEACXX_GTCCGC_5_read1.fastq.gz	Acute Myeloid Leukemia	RNA-seq	B_S005EJ11
Blueprint	151209_SN546_0285_A_H3WLNADXX_GTGAAA_1_read1.fastq.gz	Acute Myeloid Leukemia	RNA-seq	B_S013QW11
Blueprint	151209_SN546_0285_A_H3WLNADXX_GTGAAA_1_read2.fastq.gz	Acute Myeloid Leukemia	RNA-seq	B_S013QW11
Blueprint	150226_SN935_0006_B_C5YBTACXX_ACTGAT_3_read2.fastq.gz	Acute Myeloid Leukemia	RNA-seq	B_S005FH11
Blueprint	150226_SN935_0006_B_C5YBTACXX_ACTGAT_3_read1.fastq.gz	Acute Myeloid Leukemia	RNA-seq	B_S005FH11
Blueprint	150429_SN935_0015_A_C5RHEACXX_TTAGGC_7_read2.fastq.gz	Acute Myeloid Leukemia	RNA-seq	B_S00XXH11
Blueprint	150429_SN935_0015_A_C5RHEACXX_TTAGGC_7_read1.fastq.gz	Acute Myeloid Leukemia	RNA-seq	B_S00XXH11
Blueprint	150226_SN935_0006_B_C5YBTACXX_GCCAAT_1_read1.fastq.gz	Acute Myeloid Leukemia	RNA-seq	B_S00CXR11

Blueprint	150226_SN935_0006_B_C5YBTACXX_GCCAAT_1_read2.fastq.gz	Acute Myeloid Leukemia	RNA-seq	B_S00CXR11
Blueprint	150603_SN935_0017_A_C73GHACXX_GGCTAC_1_read1.fastq.gz	Acute Myeloid Leukemia	RNA-seq	B_S013N111
Blueprint	150603_SN935_0017_A_C73GHACXX_GGCTAC_1_read2.fastq.gz	Acute Myeloid Leukemia	RNA-seq	B_S013N111
Blueprint	150226_SN935_0006_B_C5YBTACXX_GTCCGC_3_read2.fastq.gz	Acute Myeloid Leukemia	RNA-seq	B_S00CYP11
Blueprint	150226_SN935_0006_B_C5YBTACXX_GTCCGC_3_read1.fastq.gz	Acute Myeloid Leukemia	RNA-seq	B_S00CYP11
Blueprint	150429_SN935_0015_A_C5RHEACXX_CGATGT_4_read2.fastq.gz	Acute Myeloid Leukemia	RNA-seq	B_S00D5511
Blueprint	150429_SN935_0015_A_C5RHEACXX_CGATGT_4_read1.fastq.gz	Acute Myeloid Leukemia	RNA-seq	B_S00D5511
Blueprint	150226_SN935_0006_B_C5YBTACXX_ACAGTG_2_read2.fastq.gz	Acute Myeloid Leukemia	RNA-seq	B_S00D3911
Blueprint	150226_SN935_0006_B_C5YBTACXX_ACAGTG_2_read1.fastq.gz	Acute Myeloid Leukemia	RNA-seq	B_S00D3911
Blueprint	150429_SN935_0015_A_C5RHEACXX_TGACCA_8_read2.fastq.gz	Acute Myeloid Leukemia	RNA-seq	B_S00XUN11
Blueprint	150429_SN935_0015_A_C5RHEACXX_TGACCA_8_read1.fastq.gz	Acute Myeloid Leukemia	RNA-seq	B_S00XUN11
Blueprint	150429_SN935_0015_A_C5RHEACXX_ACTTGA_2_read2.fastq.gz	Acute Myeloid Leukemia	RNA-seq	B_S00XYF11
Blueprint	150429_SN935_0015_A_C5RHEACXX_ACTTGA_2_read1.fastq.gz	Acute Myeloid Leukemia	RNA-seq	B_S00XYF11
Blueprint	150429_SN935_0015_A_C5RHEACXX_CAGATC_3_read2.fastq.gz	Acute Myeloid Leukemia	RNA-seq	B_S013PY11
Blueprint	150429_SN935_0015_A_C5RHEACXX_CAGATC_3_read1.fastq.gz	Acute Myeloid Leukemia	RNA-seq	B_S013PY11
Blueprint	150429_SN935_0015_A_C5RHEACXX_GATCAG_7_read1.fastq.gz	Acute Myeloid Leukemia	RNA-seq	B_S00Y1311
Blueprint	150429_SN935_0015_A_C5RHEACXX_GATCAG_7_read2.fastq.gz	Acute Myeloid Leukemia	RNA-seq	B_S00Y1311
Blueprint	150429_SN935_0015_A_C5RHEACXX_TGACCA_2_read2.fastq.gz	Acute Myeloid Leukemia	RNA-seq	B_S00XWJ11
Blueprint	150429_SN935_0015_A_C5RHEACXX_TGACCA_2_read1.fastq.gz	Acute Myeloid Leukemia	RNA-seq	B_S00XWJ11
Blueprint	150603_SN935_0017_A_C73GHACXX_CAGATC_1_read1.fastq.gz	Acute Myeloid Leukemia	RNA-seq	B_S013RU11
Blueprint	150603_SN935_0017_A_C73GHACXX_CAGATC_1_read2.fastq.gz	Acute Myeloid Leukemia	RNA-seq	B_S013RU11
Blueprint	150226_SN935_0006_B_C5YBTACXX_GTGAAA_2_read2.fastq.gz	Acute Myeloid Leukemia	RNA-seq	B_S00D1D11
Blueprint	150226_SN935_0006_B_C5YBTACXX_GTGAAA_2_read1.fastq.gz	Acute Myeloid Leukemia	RNA-seq	B_S00D1D11
Blueprint	150429_SN935_0015_A_C5RHEACXX_GGCTAC_3_read2.fastq.gz	Acute Myeloid Leukemia	RNA-seq	B_S00Y0511
Blueprint	150429_SN935_0015_A_C5RHEACXX_GGCTAC_3_read1.fastq.gz	Acute Myeloid Leukemia	RNA-seq	B_S00Y0511
Blueprint	150429_SN935_0015_A_C5RHEACXX_ACTGAT_5_read2.fastq.gz	Acute Myeloid Leukemia	RNA-seq	B_S00D4711
Blueprint	150429_SN935_0015_A_C5RHEACXX_ACTGAT_5_read1.fastq.gz	Acute Myeloid Leukemia	RNA-seq	B_S00D4711
Blueprint	150603_SN935_0017_A_C73GHACXX_CGATGT_2_read1.fastq.gz	Acute Myeloid Leukemia	RNA-seq	B_S00XVL11
Blueprint	150603_SN935_0017_A_C73GHACXX_CGATGT_2_read2.fastq.gz	Acute Myeloid Leukemia	RNA-seq	B_S00XVL11
Blueprint	150429_SN935_0015_A_C5RHEACXX_GTGAAA_4_read2.fastq.gz	Acute Myeloid Leukemia	RNA-seq	B_S013SS11
Blueprint	150429_SN935_0015_A_C5RHEACXX_GTGAAA_4_read1.fastq.gz	Acute Myeloid Leukemia	RNA-seq	B_S013SS11
Blueprint	150429_SN935_0015_A_C5RHEACXX_AGTCAA_6_read1.fastq.gz	Acute Myeloid Leukemia	RNA-seq	B_00Y6U11
Blueprint	150429_SN935_0015_A_C5RHEACXX_AGTCAA_6_read2.fastq.gz	Acute Myeloid Leukemia	RNA-seq	B_00Y6U11
Blueprint	150226_SN935_0006_B_C5YBTACXX_CTTGTA_1_read1.fastq.gz	Acute Myeloid Leukemia	RNA-seq	B_00CWT11
Blueprint	150226_SN935_0006_B_C5YBTACXX_CTTGTA_1_read2.fastq.gz	Acute Myeloid Leukemia	RNA-seq	B_00CWT11
Blueprint	150429_SN935_0015_A_C5RHEACXX_ACAGTG_6_read2.fastq.gz	Acute Myeloid Leukemia	RNA-seq	B_00Y4Y11
Blueprint	150429_SN935_0015_A_C5RHEACXX_ACAGTG_6_read1.fastq.gz	Acute Myeloid Leukemia	RNA-seq	B_00Y4Y11
Blueprint	130919_SN546_0217_B_C2GFPACXX_GATCAG_5_read2.fastq.gz	macrophage	RNA-seq	B_0022I12
Blueprint	130919_SN546_0217_B_C2GFPACXX_GATCAG_5_read1.fastq.gz	macrophage	RNA-seq	B_S0022I12
Blueprint	130919_SN546_0217_B_C2GFPACXX_ACTGAT_3_read1.fastq.gz	macrophage	RNA-seq	B_C005VG11
Blueprint	130919_SN546_0217_B_C2GFPACXX_ACTGAT_3_read2.fastq.gz	macrophage	RNA-seq	B_C005VG11
Blueprint	150917_SN546_0282_B_C7C6TACXX_GTCCGC_8_read1.fastq.gz	Chronic Lymphocytic Leukemia	RNA-seq	B_S00B0N11
Blueprint	150917_SN546_0282_B_C7C6TACXX_GTCCGC_8_read2.fastq.gz	Chronic Lymphocytic Leukemia	RNA-seq	B_S00B0N11
Blueprint	150603_SN935_0017_A_C73GHACXX_GCCAAT_8_read2.fastq.gz	Chronic Lymphocytic Leukemia	RNA-seq	B_S00B2J11
Blueprint	150603_SN935_0017_A_C73GHACXX_GCCAAT_8_read1.fastq.gz	Chronic Lymphocytic Leukemia	RNA-seq	B_S00B2J11
Blueprint	C2MVTACXX_lane7_4642_GCCAAT_L007_R1.fastq.gz	macrophage - T=6days B-glucan	RNA-seq	B_S00C0J11
Blueprint	C3JYVACXX_lane6_5386_GCCAAT_L006_R1.fastq.gz	macrophage - T=6days B-glucan	RNA-seq	B_S00HTF11
Blueprint	C3JYVACXX_lane6_5390_TTAGGC_L006_R1.fastq.gz	macrophage - T=6days B-glucan	RNA-seq	B_S00JS911
Blueprint	C2NTEACXX_lane7_4820_GCCAAT_L007_R1.fastq.gz	macrophage - T=6days B-glucan	RNA-seq	B_S00CTZ11
Blueprint	s_120830_6.ACAGTG.read1.fastq.gz	CD14-positive, CD16-negative classical monocyte	RNA-seq	B_C0010KB1
Blueprint	s_120830_6.ACAGTG.read2.fastq.gz	CD14-positive, CD16-negative classical monocyte	RNA-seq	B_C0010KB1
Blueprint	s_120830_3.TTAGGC.read1.fastq.gz	CD14-positive, CD16-negative classical monocyte	RNA-seq	B_C001UYB4
Blueprint	s_120830_3.TTAGGC.read2.fastq.gz	CD14-positive, CD16-negative classical monocyte	RNA-seq	B_C001UYB4
Blueprint	s_120830_5.AGTTCC.read1.fastq.gz	CD14-positive, CD16-negative classical monocyte	RNA-seq	B_C0011IB1
Blueprint	s_120830_5.AGTTCC.read2.fastq.gz	CD14-positive, CD16-negative classical monocyte	RNA-seq	B_C0011IB1

Blueprint	C20FWACXX_lane5_4172_ACAGTG_L005_R1.fastq.gz	CD8-positive, alpha-beta T cell	DNase1-seq	B_C0066P12
Blueprint	C20BGACXX_lane6_3758_CAGATC_L006_R1.fastq.gz	CD14-positive, CD16-negative classical monocyte	DNase1-seq	B_C005PS12
Blueprint	C2U81ACXX_lane8_4901_CGATGT_L008_R1.fastq.gz	Acute Lymphocytic Leukemia	DNase1-seq	B_S00DFM11
Blueprint	C3JYVACXX_lane8_5397_ACAGTG_L008_R1.fastq.gz	macrophage - T=6days LPS	DNase1-seq	B_S00HSH11
Blueprint	C3JYVACXX_lane7_5401_ACTTGA_L007_R1.fastq.gz	macrophage - T=6days LPS	DNase1-seq	B_S00JRB11
Blueprint	C2MVTACXX_lane8_4644_TGACCA_L008_R1.fastq.gz	macrophage - T=6days LPS	DNase1-seq	B_S00BYT11
Blueprint	C2N9FACXX_lane8_4644_TGACCA_L008_R1.fastq.gz	macrophage - T=6days LPS	DNase1-seq	B_S00BYT11
Blueprint	C2NE2ACXX_lane8_4754_GATCAG_L008_R1.fastq.gz	macrophage - T=6days LPS	DNase1-seq	B_S00CS011
Blueprint	C2NTEACXX_lane8_4754_GATCAG_L008_R1.fastq.gz	macrophage - T=6days LPS	DNase1-seq	B_S00CS011
Blueprint	C20BGACXX_lane5_3685_GCCAAT_L005_R1.fastq.gz	CD34-negative, CD41-positive, CD42-positive megakaryocyte cell	DNase1-seq	B_C006NSB1
Blueprint	C5TLLACXX_lane7_6736_GGCTAC_L007_R1.fastq.gz	CD34-negative, CD41-positive, CD42-positive megakaryocyte cell	DNase1-seq	B_S004BT
Blueprint	C5TLLACXX_lane7_6737_AGTCAA_L007_R1.fastq.gz	CD4-positive, alpha-beta T cell	DNase1-seq	B_S008H111
Blueprint	C7KUGACXX_lane6_7444_CAGATC_L006_R1.fastq.gz	erythroblast	DNase1-seq	B_S002R512
Blueprint	C6LR9ACXX_lane8_7443_GCCAAT_L008_R1.fastq.gz	erythroblast	DNase1-seq	B_S002S312
Blueprint	C2NTUACXX_lane8_4646_GCCAAT_L008_R1.fastq.gz	macrophage	DNase1-seq	B_S001S714
Blueprint	C20BGACXX_lane5_3687_CTTGTA_L005_R1.fastq.gz	inflammatory macrophage	DNase1-seq	B_S001MJ12
Blueprint	C2NTUACXX_lane8_4648_CTTGTA_L008_R1.fastq.gz	inflammatory macrophage	DNase1-seq	B_S0022I14
Blueprint	C3JYVACXX_lane7_5396_TGACCA_L007_R1.fastq.gz	macrophage - T=6days untreated	DNase1-seq	B_S00HRJ11
Blueprint	C2N9FACXX_lane8_4643_CGATGT_L008_R1.fastq.gz	macrophage - T=6days untreated	DNase1-seq	B_S00BXV11
Blueprint	C2MVTACXX_lane8_4643_CGATGT_L008_R1.fastq.gz	macrophage - T=6days untreated	DNase1-seq	B_S00BXV11
Blueprint	C2NTEACXX_lane8_4753_ACTTGA_L008_R1.fastq.gz	macrophage - T=6days untreated	DNase1-seq	B_S00CR211
Blueprint	C2NE2ACXX_lane8_4753_ACTTGA_L008_R1.fastq.gz	macrophage - T=6days untreated	DNase1-seq	B_S00CR211
Blueprint	C3JYVACXX_lane7_5400_TTAGGC_L007_R1.fastq.gz	macrophage - T=6days untreated	DNase1-seq	B_S00JQD11
Blueprint	C791JACXX_lane1_7991_AGTCAA_L001_R1.fastq.gz	Acute Myeloid Leukemia	DNase1-seq	B_S013M311
Blueprint	C4Y81ACXX_lane7_5974_TAGCTT_L007_R1.fastq.gz	Acute Myeloid Leukemia	DNase1-seq	B_S00D0F11
Blueprint	C5682ACXX_lane7_5965_TGACCA_L007_R1.fastq.gz	Acute Myeloid Leukemia	DNase1-seq	B_S00D6311
Blueprint	C20FWACXX_lane3_4175_CTTGTA_L003_R1.fastq.gz	Acute Myeloid Leukemia	DNase1-seq	B_S005EJ11
Blueprint	C7KUGACXX_lane3_7994_CCGTCC_L003_R1.fastq.gz	Acute Myeloid Leukemia	DNase1-seq	B_S013QW11
Blueprint	C20BGACXX_lane6_3754_ACAGTG_L006_R1.fastq.gz	Acute Myeloid Leukemia	DNase1-seq	B_S005FH11
Blueprint	C68R7ACXX_lane5_6919_TTAGGC_L005_R1.fastq.gz	Acute Myeloid Leukemia	DNase1-seq	B_S00XXH11
Blueprint	C55VCACXX_lane8_5975_GGCTAC_L008_R1.fastq.gz	Acute Myeloid Leukemia	DNase1-seq	B_S00CXR11
Blueprint	C791JACXX_lane1_7992_AGTTCC_L001_R1.fastq.gz	Acute Myeloid Leukemia	DNase1-seq	B_S013N111
Blueprint	C5682ACXX_lane7_5971_TTAGGC_L007_R1.fastq.gz	Acute Myeloid Leukemia	DNase1-seq	B_S00CYP11
Blueprint	C4Y81ACXX_lane8_5970_ATCACG_L008_R1.fastq.gz	Acute Myeloid Leukemia	DNase1-seq	B_S00D5511
Blueprint	C4Y81ACXX_lane8_5972_ACTTGA_L008_R1.fastq.gz	Acute Myeloid Leukemia	DNase1-seq	B_S00D3911
Blueprint	C68R7ACXX_lane5_6913_GAGTGG_L005_R1.fastq.gz	Acute Myeloid Leukemia	DNase1-seq	B_S00XUN11
Blueprint	C6957ACXX_lane6_6920_ACTTGA_L006_R1.fastq.gz	Acute Myeloid Leukemia	DNase1-seq	B_S00XYF11
Blueprint	C7KUGACXX_lane3_7993_ATGTCA_L003_R1.fastq.gz	Acute Myeloid Leukemia	DNase1-seq	B_S013PY11
Blueprint	C68R7ACXX_lane8_6922_TAGCTT_L008_R1.fastq.gz	Acute Myeloid Leukemia	DNase1-seq	B_S00Y1311
Blueprint	C68R7ACXX_lane8_6923_GGCTAC_L008_R1.fastq.gz	Acute Myeloid Leukemia	DNase1-seq	B_S00Y1311
Blueprint	C6957ACXX_lane5_6917_ATCACG_L005_R1.fastq.gz	Acute Myeloid Leukemia	DNase1-seq	B_S00XWJ11
Blueprint	C7DJ1ACXX_lane2_7995_GTAGAG_L002_R1.fastq.gz	Acute Myeloid Leukemia	DNase1-seq	B_S013RU11
Blueprint	C5682ACXX_lane7_5966_ACAGTG_L007_R1.fastq.gz	Acute Myeloid Leukemia	DNase1-seq	B_S00D1D11
Blueprint	C68R7ACXX_lane8_6921_GATCAG_L008_R1.fastq.gz	Acute Myeloid Leukemia	DNase1-seq	B_S00Y0511
Blueprint	C5682ACXX_lane8_5968_CAGATC_L008_R1.fastq.gz	Acute Myeloid Leukemia	DNase1-seq	B_S00D4711
Blueprint	C6957ACXX_lane3_6914_GGTAGC_L003_R1.fastq.gz	Acute Myeloid Leukemia	DNase1-seq	B_S00XVL11
Blueprint	C7KUGACXX_lane3_7996_GTCCGC_L003_R1.fastq.gz	Acute Myeloid Leukemia	DNase1-seq	B_S013SS11
Blueprint	C6957ACXX_lane7_6926_ACAGTG_L007_R1.fastq.gz	Acute Myeloid Leukemia	DNase1-seq	B_S00Y6U11
Blueprint	C4Y81ACXX_lane7_5967_GCCAAT_L007_R1.fastq.gz	Acute Myeloid Leukemia	DNase1-seq	B_S00CWT11
Blueprint	C6957ACXX_lane6_6924_CGATGT_L006_R1.fastq.gz	Acute Myeloid Leukemia	DNase1-seq	B_S00Y4Y11
Blueprint	C2NTUACXX_lane8_4647_CAGATC_L008_R1.fastq.gz	macrophage	DNase1-seq	B_S0022I12
Blueprint	D1YHUACXX_lane8_3684_ACAGTG_L008_R1.fastq.gz	macrophage	DNase1-seq	B_C005VG11
Blueprint	C68R7ACXX_lane5_7046_GCCAAT_L005_R1.fastq.gz	Chronic Lymphocytic Leukemia	DNase1-seq	B_S00B0N11
Blueprint	C5NY1ACXX_lane8_6126_ATCACG_L008_R1.fastq.gz	Chronic Lymphocytic Leukemia	DNase1-seq	B_S00B2J11
Blueprint	C2MVTACXX_lane8_4645_ACAGTG_L008_R1.fastq.gz	macrophage - T=6days B-glucan	DNase1-seq	B_S00C0J11
Blueprint	C3JYVACXX_lane7_5398_GCCAAT_L007_R1.fastq.gz	macrophage - T=6days B-glucan	DNase1-seq	B_S00HTF11

Blueprint	C3JYVACXX_lane7_5402_GATCAG_L007_R1.fastq.gz	macrophage - T=6days B-glucan	DNase1-seq	B_S00JS911
Blueprint	C2NE2ACXX_lane8_4755_TAGCTT_L008_R1.fastq.gz	macrophage - T=6days B-glucan	DNase1-seq	B_S00CTZ11
Blueprint	D1FB1ACXX_lane8_3324_ACAGTG_L008_R1.fastq.gz	CD14-positive, CD16-negative classical monocyte	DNase1-seq	B_C0010KB1
Blueprint	D1FB1ACXX_lane6_3326_CAGATC_L006_R1.fastq.gz	CD14-positive, CD16-negative classical monocyte	DNase1-seq	B_C001UYB4
Blueprint	D1FB1ACXX_lane8_3323_TGACCA_L008_R1.fastq.gz	CD14-positive, CD16-negative classical monocyte	DNase1-seq	B_C0011IB1
Roadmap	ENCFF583FQV	skin fibroblast	RNA-seq	R_ENCSR637GBV
Roadmap	ENCFF249BIO	skin fibroblast	RNA-seq	R_ENCSR655XQF
Roadmap	ENCFF946MIY	skin fibroblast	RNA-seq	R_ENCSR022MON
Roadmap	ENCFF533GEZ	skin fibroblast	RNA-seq	R_ENCSR022MON
Roadmap	ENCFF273FCE	skin fibroblast	RNA-seq	R_ENCSR982VYI
Roadmap	ENCFF224MVX	fibroblast of skin of abdomen	RNA-seq	R_ENCSR361DRG
Roadmap	ENCFF079EAZ	fibroblast of skin of abdomen	RNA-seq	R_ENCSR681ALA
Roadmap	ENCFF105OLM	IMR-90	RNA-seq	R_ENCSR424FAZ
Roadmap	ENCFF381KYM	IMR-90	RNA-seq	R_ENCSR424FAZ
Roadmap	ENCFF547LGQ	IMR-90	RNA-seq	R_ENCSR424FAZ
Roadmap	ENCFF094HAH	IMR-90	RNA-seq	R_ENCSR424FAZ
Roadmap	ENCFF714LDG	trophoblast cell	RNA-seq	R_ENCSR762CJN
Roadmap	ENCFF629SLX	muscle of arm	RNA-seq	R_ENCSR406YML
Roadmap	ENCFF319ZJP	muscle of arm	RNA-seq	R_ENCSR364IBB
Roadmap	ENCFF070JPZ	muscle of arm	RNA-seq	R_ENCSR364IBB
Roadmap	ENCFF269DXG	muscle of arm	RNA-seq	R_ENCSR317LMH
Roadmap	ENCFF442QQD	muscle of arm	RNA-seq	R_ENCSR620ZNQ
Roadmap	ENCFF520PYM	muscle of arm	RNA-seq	R_ENCSR305NXN
Roadmap	ENCFF617CUF	muscle of arm	RNA-seq	R_ENCSR677MYO
Roadmap	ENCFF691YZM	muscle of arm	RNA-seq	R_ENCSR990LHE
Roadmap	ENCFF713VFN	stomach	RNA-seq	R_ENCSR922VBO
Roadmap	ENCFF794IMD	stomach	RNA-seq	R_ENCSR721HDG
Roadmap	ENCFF433BMT	stomach	RNA-seq	R_ENCSR702IGQ
Roadmap	ENCFF824JKN	stomach	RNA-seq	R_ENCSR549DVY
Roadmap	ENCFF314DZG	stomach	RNA-seq	R_ENCSR783BUO
Roadmap	ENCFF437SZX	stomach	RNA-seq	R_ENCSR783BUO
Roadmap	ENCFF838LGI	stomach	RNA-seq	R_ENCSR951NPS
Roadmap	ENCFF383KNO	stomach	RNA-seq	R_ENCSR123ZCX
Roadmap	ENCFF701DLG	stomach	RNA-seq	R_ENCSR774SEX
Roadmap	ENCFF326BCF	muscle of back	RNA-seq	R_ENCSR729ZII
Roadmap	ENCFF851VPL	muscle of back	RNA-seq	R_ENCSR806ESH
Roadmap	ENCFF093TMQ	muscle of back	RNA-seq	R_ENCSR995ORR
Roadmap	ENCFF670EYD	muscle of back	RNA-seq	R_ENCSR891JVD
Roadmap	ENCFF473DKB	muscle of back	RNA-seq	R_ENCSR652AWW
Roadmap	ENCFF447GUA	muscle of back	RNA-seq	R_ENCSR027EJD
Roadmap	ENCFF630XRG	muscle of back	RNA-seq	R_ENCSR576UKA
Roadmap	ENCFF096QFW	muscle of back	RNA-seq	R_ENCSR094RGI
Roadmap	ENCFF683XJX	muscle of back	RNA-seq	R_ENCSR239BBI
Roadmap	ENCFF015SQM	muscle of back	RNA-seq	R_ENCSR522XTV
Roadmap	ENCFF025DKC	small intestine	RNA-seq	R_ENCSR719HRO
Roadmap	ENCFF531PQC	small intestine	RNA-seq	R_ENCSR621FYE
Roadmap	ENCFF796HDN	small intestine	RNA-seq	R_ENCSR150JIX
Roadmap	ENCFF974KOL	small intestine	RNA-seq	R_ENCSR446RKD
Roadmap	ENCFF677RLO	small intestine	RNA-seq	R_ENCSR523EDD
Roadmap	ENCFF038OLY	muscle of leg	RNA-seq	R_ENCSR096USV
Roadmap	ENCFF591YBG	muscle of leg	RNA-seq	R_ENCSR860DST
Roadmap	ENCFF610CFP	muscle of leg	RNA-seq	R_ENCSR144UVO
Roadmap	ENCFF726EJA	muscle of leg	RNA-seq	R_ENCSR144UVO
Roadmap	ENCFF969AEA	muscle of leg	RNA-seq	R_ENCSR545WAC

Roadmap	ENCFF280FJO	muscle of leg	RNA-seq	R_ENCSR174ESD
Roadmap	ENCFF678IQL	muscle of leg	RNA-seq	R_ENCSR086DZF
Roadmap	ENCFF755EWN	muscle of leg	RNA-seq	R_ENCSR561WEX
Roadmap	ENCFF823WWJ	muscle of leg	RNA-seq	R_ENCSR447IJE
Roadmap	ENCFF800RTW	large intestine	RNA-seq	R_ENCSR286KWP
Roadmap	ENCFF062QDJ	large intestine	RNA-seq	R_ENCSR859KGW
Roadmap	ENCFF376BOA	large intestine	RNA-seq	R_ENCSR859KGW
Roadmap	ENCFF132LZV	large intestine	RNA-seq	R_ENCSR777ONH
Roadmap	ENCFF600PXN	large intestine	RNA-seq	R_ENCSR930URM
Roadmap	ENCFF203FUW	large intestine	RNA-seq	R_ENCSR857VKL
Roadmap	ENCFF472AVS	large intestine	RNA-seq	R_ENCSR363BVC
Roadmap	ENCFF994THJ	left lung	RNA-seq	R_ENCSR861SOG
Roadmap	ENCFF565ABG	left lung	RNA-seq	R_ENCSR733MWN
Roadmap	ENCFF836QCP	left lung	RNA-seq	R_ENCSR592EZK
Roadmap	ENCFF810HYB	left lung	RNA-seq	R_ENCSR499NEL
Roadmap	ENCFF527HNS	left lung	RNA-seq	R_ENCSR222IGR
Roadmap	ENCFF391AKU	left lung	RNA-seq	R_ENCSR572FXC
Roadmap	ENCFF715EGE	kidney	RNA-seq	R_ENCSR907KDH
Roadmap	ENCFF391AJE	kidney	RNA-seq	R_ENCSR212AMA
Roadmap	ENCFF390GZH	kidney	RNA-seq	R_ENCSR896QPD
Roadmap	ENCFF959UJB	kidney	RNA-seq	R_ENCSR495UXA
Roadmap	ENCFF240TXD	right lung	RNA-seq	R_ENCSR554KBK
Roadmap	ENCFF994CGB	right lung	RNA-seq	R_ENCSR074APH
Roadmap	ENCFF299JVW	right lung	RNA-seq	R_ENCSR560MDQ
Roadmap	ENCFF567KUC	right lung	RNA-seq	R_ENCSR176WMG
Roadmap	ENCFF527HRM	right lung	RNA-seq	R_ENCSR044JAQ
Roadmap	ENCFF822QPE	thymus	RNA-seq	R_ENCSR367QHR
Roadmap	ENCFF410KEZ	thymus	RNA-seq	R_ENCSR158XIJ
Roadmap	ENCFF715OSA	thymus	RNA-seq	R_ENCSR069CMT
Roadmap	ENCFF199PMN	thymus	RNA-seq	R_ENCSR175CNQ
Roadmap	ENCFF649RZG	thymus	RNA-seq	R_ENCSR175CNQ
Roadmap	ENCFF975AUW	heart	RNA-seq	R_ENCSR047LLJ
Roadmap	ENCFF092JPL	heart	RNA-seq	R_ENCSR863BUL
Roadmap	ENCFF149FEQ	renal cortex interstitium	RNA-seq	R_ENCSR328PVI
Roadmap	ENCFF371QWQ	renal cortex interstitium	RNA-seq	R_ENCSR899SWV
Roadmap	ENCFF826YSU	renal cortex interstitium	RNA-seq	R_ENCSR436ZKE
Roadmap	ENCFF051TUA	renal cortex interstitium	RNA-seq	R_ENCSR436ZKE
Roadmap	ENCFF479AQV	adrenal gland	RNA-seq	R_ENCSR335GET
Roadmap	ENCFF396DVW	adrenal gland	RNA-seq	R_ENCSR120NEA
Roadmap	ENCFF080VMX	adrenal gland	RNA-seq	R_ENCSR688YOZ
Roadmap	ENCFF345CEU	adrenal gland	RNA-seq	R_ENCSR740OPV
Roadmap	ENCFF953VOF	renal pelvis	RNA-seq	R_ENCSR424TSZ
Roadmap	ENCFF993WUV	renal pelvis	RNA-seq	R_ENCSR204XBB
Roadmap	ENCFF329YXL	renal pelvis	RNA-seq	R_ENCSR929KRW
Roadmap	ENCFF960GCL	renal pelvis	RNA-seq	R_ENCSR929KRW
Roadmap	ENCFF956SGC	left kidney	RNA-seq	R_ENCSR702IMR
Roadmap	ENCFF859IDO	left renal cortex interstitium	RNA-seq	R_ENCSR015EMF
Roadmap	ENCFF832ENU	left renal cortex interstitium	RNA-seq	R_ENCSR125NGM
Roadmap	ENCFF139JYD	left renal cortex interstitium	RNA-seq	R_ENCSR125NGM
Roadmap	ENCFF356DVL	left renal cortex interstitium	RNA-seq	R_ENCSR759WPF
Roadmap	ENCFF847ODW	left renal cortex interstitium	RNA-seq	R_ENCSR413LXW
Roadmap	ENCFF490TMA	left renal pelvis	RNA-seq	R_ENCSR029FTY
Roadmap	ENCFF453MGJ	left renal pelvis	RNA-seq	R_ENCSR321ROU

Roadmap	ENCFF592GJW	left renal pelvis	RNA-seq	R_ENCSR410DUZ
Roadmap	ENCFF493PDU	left renal pelvis	RNA-seq	R_ENCSR160UAZ
Roadmap	ENCFF754GRT	right renal pelvis	RNA-seq	R_ENCSR552YAE
Roadmap	ENCFF791IHU	right renal pelvis	RNA-seq	R_ENCSR352GCS
Roadmap	ENCFF260HSY	right renal pelvis	RNA-seq	R_ENCSR352GCS
Roadmap	ENCFF178QHC	right renal pelvis	RNA-seq	R_ENCSR543TQW
Roadmap	ENCFF853QBA	right renal pelvis	RNA-seq	R_ENCSR928CEQ
Roadmap	ENCFF708BFX	spinal cord	RNA-seq	R_ENCSR899NLW
Roadmap	ENCFF725KJB	spinal cord	RNA-seq	R_ENCSR899NLW
Roadmap	ENCFF123FVX	spinal cord	RNA-seq	R_ENCSR333FZW
Roadmap	ENCFF659JRI	spinal cord	RNA-seq	R_ENCSR333FZW
Roadmap	ENCFF701WAW	right renal cortex interstitium	RNA-seq	R_ENCSR822AOE
Roadmap	ENCFF589SOA	right renal cortex interstitium	RNA-seq	R_ENCSR884EVS
Roadmap	ENCFF711XVI	right renal cortex interstitium	RNA-seq	R_ENCSR400DJE
Roadmap	ENCFF098SSN	spleen	RNA-seq	R_ENCSR265NZF
Roadmap	ENCFF732SOA	psoas muscle	RNA-seq	R_ENCSR817TLH
Roadmap	ENCFF703HYW	muscle of trunk	RNA-seq	R_ENCSR531RKI
Roadmap	ENCFF102XVK	ovary	RNA-seq	R_ENCSR727VTD
Roadmap	ENCFF855YTN	ovary	RNA-seq	R_ENCSR725TPW
Roadmap	ENCFF632WEO	pancreas	RNA-seq	R_ENCSR629VMZ
Roadmap	ENCFF075UBV	pancreas	RNA-seq	R_ENCSR571BML
Roadmap	ENCFF276UME	testis	RNA-seq	R_ENCSR755LFM
Roadmap	ENCFF673MWT	forelimb muscle	RNA-seq	R_ENCSR711NGL
Roadmap	ENCFF976CLZ	hindlimb muscle	RNA-seq	R_ENCSR516VDS
Roadmap	ENCFF990ZUE	H1-hESC	RNA-seq	R_ENCSR911GQI
Roadmap	ENCFF307UVF	H1-hESC	RNA-seq	R_ENCSR844HLP
Blueprint	ENCFF426NOK	skin fibroblast	DNase1-seq	R_ENCBS336CDQ
Blueprint	ENCFF976EWT	skin fibroblast	DNase1-seq	R_ENCBS336CDQ
Blueprint	ENCFF638STX	skin fibroblast	DNase1-seq	R_ENCBS336CDQ
Blueprint	ENCFF761MPW	skin fibroblast	DNase1-seq	R_ENCBS336CDQ
Blueprint	ENCFF484CBD	skin fibroblast	DNase1-seq	R_ENCBS336CDQ
Blueprint	ENCFF700BMG	skin fibroblast	DNase1-seq	R_ENCBS336CDQ
Blueprint	ENCFF186NLY	skin fibroblast	DNase1-seq	R_ENCBS336CDQ
Blueprint	ENCFF645VBV	skin fibroblast	DNase1-seq	R_ENCBS336CDQ
Blueprint	ENCFF594NMP	skin fibroblast	DNase1-seq	R_ENCBS336CDQ
Blueprint	ENCFF865ORR	skin fibroblast	DNase1-seq	R_ENCBS890NFL
Blueprint	ENCFF593MYJ	skin fibroblast	DNase1-seq	R_ENCBS890NFL
Blueprint	ENCFF419BYU	skin fibroblast	DNase1-seq	R_ENCBS890NFL
Blueprint	ENCFF836RXB	skin fibroblast	DNase1-seq	R_ENCBS890NFL
Blueprint	ENCFF479NNH	skin fibroblast	DNase1-seq	R_ENCBS180EDA
Blueprint	ENCFF899QJN	skin fibroblast	DNase1-seq	R_ENCBS180EDA
Blueprint	ENCFF707PZQ	fibroblast of skin of abdomen	DNase1-seq	R_ENCBS405WVO
Blueprint	ENCFF972ZVY	fibroblast of skin of abdomen	DNase1-seq	R_ENCBS405WVO
Blueprint	ENCFF346SMK	fibroblast of skin of abdomen	DNase1-seq	R_ENCBS405WVO
Blueprint	ENCFF491YLY	fibroblast of skin of abdomen	DNase1-seq	R_ENCBS405WVO
Blueprint	ENCFF657JQO	fibroblast of skin of abdomen	DNase1-seq	R_ENCBS405WVO
Blueprint	https://www.ncbi.nlm.nih.gov/geo/query/acc.cgi?acc=GSM878636	fibroblast of skin of abdomen	DNase1-seq	R_ENCBS599YIE
Blueprint	ENCFF070YBE	IMR-90	DNase1-seq	R_ENCBS754TWW_ENCBS048TNH
Blueprint	ENCFF175PZA	IMR-90	DNase1-seq	R_ENCBS754TWW_ENCBS048TNH
Blueprint	ENCFF119LCF	trophoblast cell	DNase1-seq	R_ENCBS150HBC_ENCBS376RZJ
Blueprint	ENCFF047VYC	trophoblast cell	DNase1-seq	R_ENCBS150HBC_ENCBS376RZJ
Blueprint	ENCFF091DTH	trophoblast cell	DNase1-seq	R_ENCBS150HBC_ENCBS376RZJ
Blueprint	ENCFF982YFY	trophoblast cell	DNase1-seq	R_ENCBS150HBC_ENCBS376RZJ

Blueprint	ENCFF356LUH	trophoblast cell	DNase1-seq	R_ENCBS150HBC_ENCBS376RZJ
Blueprint	ENCFF846KKT	trophoblast cell	DNase1-seq	R_ENCBS150HBC_ENCBS376RZJ
Blueprint	ENCFF894MKA	trophoblast cell	DNase1-seq	R_ENCBS150HBC_ENCBS376RZJ
Blueprint	ENCFF149EUW	trophoblast cell	DNase1-seq	R_ENCBS150HBC_ENCBS376RZJ
Blueprint	ENCFF385MYE	trophoblast cell	DNase1-seq	R_ENCBS150HBC_ENCBS376RZJ
Blueprint	ENCFF654RBE	trophoblast cell	DNase1-seq	R_ENCBS150HBC_ENCBS376RZJ
Blueprint	ENCFF671NGZ	trophoblast cell	DNase1-seq	R_ENCBS150HBC_ENCBS376RZJ
Blueprint	ENCFF695HOH	trophoblast cell	DNase1-seq	R_ENCBS150HBC_ENCBS376RZJ
Blueprint	ENCFF145YHJ	trophoblast cell	DNase1-seq	R_ENCBS150HBC_ENCBS376RZJ
Blueprint	ENCFF041BSC	trophoblast cell	DNase1-seq	R_ENCBS150HBC_ENCBS376RZJ
Blueprint	ENCFF278YNM	trophoblast cell	DNase1-seq	R_ENCBS150HBC_ENCBS376RZJ
Blueprint	ENCFF144BFB	trophoblast cell	DNase1-seq	R_ENCBS150HBC_ENCBS376RZJ
Blueprint	ENCFF968JUL	trophoblast cell	DNase1-seq	R_ENCBS150HBC_ENCBS376RZJ
Blueprint	ENCFF459PAV	trophoblast cell	DNase1-seq	R_ENCBS150HBC_ENCBS376RZJ
Blueprint	ENCFF149ODQ	muscle of arm	DNase1-seq	R_ENCBS090AGL
Blueprint	ENCFF960XRX	muscle of arm	DNase1-seq	R_ENCBS090AGL
Blueprint	ENCFF941KYM	muscle of arm	DNase1-seq	R_ENCBS090AGL
Blueprint	ENCFF896QEW	muscle of arm	DNase1-seq	R_ENCBS090AGL
Blueprint	ENCFF273BFB	muscle of arm	DNase1-seq	R_ENCBS516CJQ
Blueprint	ENCFF825SDM	muscle of arm	DNase1-seq	R_ENCBS516CJQ
Blueprint	ENCFF027HWH	muscle of arm	DNase1-seq	R_ENCBS054WKY
Blueprint	ENCFF415GQM	muscle of arm	DNase1-seq	R_ENCBS586CPQ
Blueprint	ENCFF817VSJ	muscle of arm	DNase1-seq	R_ENCBS586CPQ
Blueprint	ENCFF840APC	muscle of arm	DNase1-seq	R_ENCBS261IIB
Blueprint	ENCFF337KND	muscle of arm	DNase1-seq	R_ENCBS180RZG
Blueprint	ENCFF443DXI	muscle of arm	DNase1-seq	R_ENCBS180RZG
Blueprint	ENCFF794SFF	muscle of arm	DNase1-seq	R_ENCBS892WJE
Blueprint	ENCFF500PCM	muscle of arm	DNase1-seq	R_ENCBS892WJE
Blueprint	ENCFF400PCJ	muscle of arm	DNase1-seq	R_ENCBS892WJE
Blueprint	ENCFF187JEI	muscle of arm	DNase1-seq	R_ENCBS892WJE
Blueprint	ENCFF762DDD	muscle of arm	DNase1-seq	R_ENCBS892WJE
Blueprint	ENCFF677QYQ	muscle of arm	DNase1-seq	R_ENCBS892WJE
Blueprint	ENCFF709WUS	muscle of arm	DNase1-seq	R_ENCBS892WJE
Blueprint	ENCFF244EEK	muscle of arm	DNase1-seq	R_ENCBS892WJE
Blueprint	ENCFF243CEU	muscle of arm	DNase1-seq	R_ENCBS892WJE
Blueprint	ENCFF502JHP	stomach	DNase1-seq	R_ENCBS578HBL
Blueprint	ENCFF044OTP	stomach	DNase1-seq	R_ENCBS578HBL
Blueprint	ENCFF726SST	stomach	DNase1-seq	R_ENCBS441WEO
Blueprint	ENCFF629RQV	stomach	DNase1-seq	R_ENCBS441WEO
Blueprint	ENCFF727OLN	stomach	DNase1-seq	R_ENCBS220QDW
Blueprint	ENCFF644PKR	stomach	DNase1-seq	R_ENCBS220QDW
Blueprint	ENCFF288CYZ	stomach	DNase1-seq	R_ENCBS220QDW
Blueprint	ENCFF970FYA	stomach	DNase1-seq	R_ENCBS246MHN
Blueprint	ENCFF141WGT	stomach	DNase1-seq	R_ENCBS291NHF
Blueprint	ENCFF667XCZ	stomach	DNase1-seq	R_ENCBS291NHF
Blueprint	ENCFF953NRZ	stomach	DNase1-seq	R_ENCBS291NHF
Blueprint	ENCFF752LNT	stomach	DNase1-seq	R_ENCBS878MRX
Blueprint	ENCFF424NPO	stomach	DNase1-seq	R_ENCBS878MRX
Blueprint	ENCFF883FMW	stomach	DNase1-seq	R_ENCBS159PIU
Blueprint	ENCFF088TXE	stomach	DNase1-seq	R_ENCBS159PIU
Blueprint	ENCFF135RGN	stomach	DNase1-seq	R_ENCBS159PIU
Blueprint	ENCFF985UXU	stomach	DNase1-seq	R_ENCBS159PIU
Blueprint	ENCFF718MLB	stomach	DNase1-seq	R_ENCBS159PIU

Blueprint	ENCFF780HTB	stomach	DNase1-seq	R_ENCBS159PIU
Blueprint	ENCFF491MFA	stomach	DNase1-seq	R_ENCBS159PIU
Blueprint	ENCFF426THC	stomach	DNase1-seq	R_ENCBS159PIU
Blueprint	ENCFF264JZE	stomach	DNase1-seq	R_ENCBS716QQK
Blueprint	ENCFF990XCE	stomach	DNase1-seq	R_ENCBS716QQK
Blueprint	ENCFF380UEX	muscle of back	DNase1-seq	R_ENCBS384LIR
Blueprint	ENCFF304VFH	muscle of back	DNase1-seq	R_ENCBS384LIR
Blueprint	ENCFF068GRT	muscle of back	DNase1-seq	R_ENCBS384LIR
Blueprint	ENCFF041HUV	muscle of back	DNase1-seq	R_ENCBS384LIR
Blueprint	ENCFF678RJO	muscle of back	DNase1-seq	R_ENCBS384LIR
Blueprint	ENCFF368HUM	muscle of back	DNase1-seq	R_ENCBS384LIR
Blueprint	ENCFF447RQF	muscle of back	DNase1-seq	R_ENCBS384LIR
Blueprint	ENCFF216MSZ	muscle of back	DNase1-seq	R_ENCBS174IGM
Blueprint	ENCFF635TUS	muscle of back	DNase1-seq	R_ENCBS345TTL
Blueprint	ENCFF972LVN	muscle of back	DNase1-seq	R_ENCBS345TTL
Blueprint	ENCFF943APV	muscle of back	DNase1-seq	R_ENCBS136SDO
Blueprint	ENCFF296VDO	muscle of back	DNase1-seq	R_ENCBS136SDO
Blueprint	ENCFF248XSX	muscle of back	DNase1-seq	R_ENCBS645HGQ
Blueprint	ENCFF527MIJ	muscle of back	DNase1-seq	R_ENCBS645HGQ
Blueprint	ENCFF844ELO	muscle of back	DNase1-seq	R_ENCBS020XIW
Blueprint	ENCFF608IJP	muscle of back	DNase1-seq	R_ENCBS020XIW
Blueprint	ENCFF225CLL	muscle of back	DNase1-seq	R_ENCBS897YOR
Blueprint	ENCFF673KNK	muscle of back	DNase1-seq	R_ENCBS897YOR
Blueprint	ENCFF282HOP	muscle of back	DNase1-seq	R_ENCBS897YOR
Blueprint	ENCFF873JMK	muscle of back	DNase1-seq	R_ENCBS479AOA
Blueprint	ENCFF961ZLA	muscle of back	DNase1-seq	R_ENCBS479AOA
Blueprint	ENCFF532NCU	muscle of back	DNase1-seq	R_ENCBS479AOA
Blueprint	ENCFF347UTJ	muscle of back	DNase1-seq	R_ENCBS479AOA
Blueprint	ENCFF446XVP	muscle of back	DNase1-seq	R_ENCBS479AOA
Blueprint	ENCFF914LBJ	muscle of back	DNase1-seq	R_ENCBS479AOA
Blueprint	ENCFF055BON	muscle of back	DNase1-seq	R_ENCBS479AOA
Blueprint	ENCFF683XJX	muscle of back	DNase1-seq	R_ENCBS825MQT
Blueprint	ENCFF735BDG	muscle of back	DNase1-seq	R_ENCBS136EGD
Blueprint	ENCFF904JYN	muscle of back	DNase1-seq	R_ENCBS136EGD
Blueprint	ENCFF102YUC	muscle of back	DNase1-seq	R_ENCBS136EGD
Blueprint	ENCFF198YRW	muscle of back	DNase1-seq	R_ENCBS136EGD
Blueprint	ENCFF665XQC	small intestine	DNase1-seq	R_ENCBS853LFM
Blueprint	ENCFF946FXI	small intestine	DNase1-seq	R_ENCBS853LFM
Blueprint	ENCFF473FIC	small intestine	DNase1-seq	R_ENCBS853LFM
Blueprint	ENCFF373XZL	small intestine	DNase1-seq	R_ENCBS853LFM
Blueprint	ENCFF570FNC	small intestine	DNase1-seq	R_ENCBS615YKY
Blueprint	ENCFF463POG	small intestine	DNase1-seq	R_ENCBS529UES
Blueprint	ENCFF698BGB	small intestine	DNase1-seq	R_ENCBS529UES
Blueprint	ENCFF098RZZ	small intestine	DNase1-seq	R_ENCBS529UES
Blueprint	ENCFF404NLX	small intestine	DNase1-seq	R_ENCBS623YHX
Blueprint	ENCFF670PIU	small intestine	DNase1-seq	R_ENCBS133LAN
Blueprint	ENCFF774AET	small intestine	DNase1-seq	R_ENCBS133LAN
Blueprint	ENCFF173RST	muscle of leg	DNase1-seq	R_ENCBS611ZBY
Blueprint	ENCFF289VMW	muscle of leg	DNase1-seq	R_ENCBS517FUR
Blueprint	ENCFF401RGR	muscle of leg	DNase1-seq	R_ENCBS517FUR
Blueprint	ENCFF050CPH	muscle of leg	DNase1-seq	R_ENCBS023IXF
Blueprint	ENCFF396CDS	muscle of leg	DNase1-seq	R_ENCBS023IXF
Blueprint	ENCFF018ZYC	muscle of leg	DNase1-seq	R_ENCBS947JRD

Blueprint	ENCFF667LLC	muscle of leg	DNase1-seq	R_ENCBS947JRD
Blueprint	ENCFF843QNZ	muscle of leg	DNase1-seq	R_ENCBS947JRD
Blueprint	ENCFF892QTV	muscle of leg	DNase1-seq	R_ENCBS011TVS
Blueprint	ENCFF941COX	muscle of leg	DNase1-seq	R_ENCBS011TVS
Blueprint	ENCFF005KIH	muscle of leg	DNase1-seq	R_ENCBS011TVS
Blueprint	ENCFF968NLM	muscle of leg	DNase1-seq	R_ENCBS011TVS
Blueprint	ENCFF308AWG	muscle of leg	DNase1-seq	R_ENCBS011TVS
Blueprint	ENCFF419QCQ	muscle of leg	DNase1-seq	R_ENCBS011TVS
Blueprint	ENCFF987FBY	muscle of leg	DNase1-seq	R_ENCBS011TVS
Blueprint	ENCFF103FXL	muscle of leg	DNase1-seq	R_ENCBS011TVS
Blueprint	ENCFF916CUH	muscle of leg	DNase1-seq	R_ENCBS099OIO
Blueprint	ENCFF792OYK	muscle of leg	DNase1-seq	R_ENCBS099OIO
Blueprint	ENCFF786MHH	muscle of leg	DNase1-seq	R_ENCBS099OIO
Blueprint	ENCFF241GTO	muscle of leg	DNase1-seq	R_ENCBS099OIO
Blueprint	ENCFF481YWO	muscle of leg	DNase1-seq	R_ENCBS099OIO
Blueprint	ENCFF505FYZ	muscle of leg	DNase1-seq	R_ENCBS099OIO
Blueprint	ENCFF269DFT	muscle of leg	DNase1-seq	R_ENCBS099OIO
Blueprint	ENCFF535RGT	muscle of leg	DNase1-seq	R_ENCBS099OIO
Blueprint	ENCFF175SLR	muscle of leg	DNase1-seq	R_ENCBS099OIO
Blueprint	ENCFF101OMS	muscle of leg	DNase1-seq	R_ENCBS099OIO
Blueprint	ENCFF361UWR	muscle of leg	DNase1-seq	R_ENCBS143XQJ
Blueprint	ENCFF742WIO	muscle of leg	DNase1-seq	R_ENCBS143XQJ
Blueprint	ENCFF272WWY	muscle of leg	DNase1-seq	R_ENCBS143XQJ
Blueprint	ENCFF808QSC	muscle of leg	DNase1-seq	R_ENCBS143XQJ
Blueprint	ENCFF128KEC	muscle of leg	DNase1-seq	R_ENCBS984JKS
Blueprint	ENCFF201FIA	muscle of leg	DNase1-seq	R_ENCBS984JKS
Blueprint	ENCFF611HRT	muscle of leg	DNase1-seq	R_ENCBS984JKS
Blueprint	ENCFF365KQE	muscle of leg	DNase1-seq	R_ENCBS984JKS
Blueprint	ENCFF365NYW	muscle of leg	DNase1-seq	R_ENCBS984JKS
Blueprint	ENCFF743SDH	large intestine	DNase1-seq	R_ENCBS997WGU
Blueprint	ENCFF004QQU	large intestine	DNase1-seq	R_ENCBS997WGU
Blueprint	ENCFF927LGL	large intestine	DNase1-seq	R_ENCBS588ZWT
Blueprint	ENCFF144ZQA	large intestine	DNase1-seq	R_ENCBS445IVN
Blueprint	ENCFF895VUK	large intestine	DNase1-seq	R_ENCBS445IVN
Blueprint	ENCFF181QVU	large intestine	DNase1-seq	R_ENCBS699KFK
Blueprint	ENCFF271BCH	large intestine	DNase1-seq	R_ENCBS867ILV
Blueprint	ENCFF122XCL	large intestine	DNase1-seq	R_ENCBS867ILV
Blueprint	ENCFF528OCE	large intestine	DNase1-seq	R_ENCBS383OVQ
Blueprint	ENCFF070ZPW	large intestine	DNase1-seq	R_ENCBS383OVQ
Blueprint	ENCFF942PFR	left lung	DNase1-seq	R_ENCBS078XUR
Blueprint	ENCFF639RTQ	left lung	DNase1-seq	R_ENCBS574MIZ
Blueprint	ENCFF641TVF	left lung	DNase1-seq	R_ENCBS859ASH
Blueprint	ENCFF666OGL	left lung	DNase1-seq	R_ENCBS143LJK
Blueprint	ENCFF461HIP	left lung	DNase1-seq	R_ENCBS143LJK
Blueprint	ENCFF244DOB	left lung	DNase1-seq	R_ENCBS143LJK
Blueprint	ENCFF497CUV	left lung	DNase1-seq	R_ENCBS143LJK
Blueprint	ENCFF551FHH	left lung	DNase1-seq	R_ENCBS143LJK
Blueprint	ENCFF977WQN	left lung	DNase1-seq	R_ENCBS143LJK
Blueprint	ENCFF925NBM	left lung	DNase1-seq	R_ENCBS143LJK
Blueprint	ENCFF999PLN	left lung	DNase1-seq	R_ENCBS143LJK
Blueprint	ENCFF135VOG	left lung	DNase1-seq	R_ENCBS143LJK
Blueprint	ENCFF450MOY	left lung	DNase1-seq	R_ENCBS516MKG
Blueprint	ENCFF962CEA	left lung	DNase1-seq	R_ENCBS516MKG

Blueprint	ENCFF191FJK	left lung	DNase1-seq	R_ENCBS117CVU
Blueprint	ENCFF145NEL	left lung	DNase1-seq	R_ENCBS117CVU
Blueprint	ENCFF347TBD	left lung	DNase1-seq	R_ENCBS117CVU
Blueprint	ENCFF272FPH	kidney	DNase1-seq	R_ENCBS478OZL
Blueprint	ENCFF083NYS	kidney	DNase1-seq	R_ENCBS478OZL
Blueprint	ENCFF916YMJ	kidney	DNase1-seq	R_ENCBS263ZZU
Blueprint	ENCFF799XLW	kidney	DNase1-seq	R_ENCBS263ZZU
Blueprint	ENCFF618FCN	kidney	DNase1-seq	R_ENCBS263ZZU
Blueprint	ENCFF970TXW	kidney	DNase1-seq	R_ENCBS434EOI
Blueprint	ENCFF009RHR	kidney	DNase1-seq	R_ENCBS434EOI
Blueprint	ENCFF196NVH	kidney	DNase1-seq	R_ENCBS434EOI
Blueprint	ENCFF753ELF	kidney	DNase1-seq	R_ENCBS034SKE
Blueprint	ENCFF286XQY	kidney	DNase1-seq	R_ENCBS034SKE
Blueprint	ENCFF416YBE	right lung	DNase1-seq	R_ENCBS917VNB
Blueprint	ENCFF120FQU	right lung	DNase1-seq	R_ENCBS917VNB
Blueprint	ENCFF427YAL	right lung	DNase1-seq	R_ENCBS993PWO
Blueprint	ENCFF346VUR	right lung	DNase1-seq	R_ENCBS993PWO
Blueprint	ENCFF776KRS	right lung	DNase1-seq	R_ENCBS467PJB
Blueprint	ENCFF922IPA	right lung	DNase1-seq	R_ENCBS467PJB
Blueprint	ENCFF257CYL	right lung	DNase1-seq	R_ENCBS421OZO
Blueprint	ENCFF514LUU	right lung	DNase1-seq	R_ENCBS421OZO
Blueprint	ENCFF083OYH	right lung	DNase1-seq	R_ENCBS421OZO
Blueprint	ENCFF723TEH	right lung	DNase1-seq	R_ENCBS122USS
Blueprint	ENCFF973AFO	right lung	DNase1-seq	R_ENCBS122USS
Blueprint	ENCFF702CZA	thymus	DNase1-seq	R_ENCBS484BGT
Blueprint	ENCFF714BGJ	thymus	DNase1-seq	R_ENCBS484BGT
Blueprint	ENCFF284FZH	thymus	DNase1-seq	R_ENCBS948PMG
Blueprint	ENCFF496OMB	thymus	DNase1-seq	R_ENCBS948PMG
Blueprint	ENCFF901HTM	thymus	DNase1-seq	R_ENCBS948PMG
Blueprint	ENCFF939NEZ	thymus	DNase1-seq	R_ENCBS054CPR
Blueprint	ENCFF518NCM	thymus	DNase1-seq	R_ENCBS054CPR
Blueprint	ENCFF806QFV	thymus	DNase1-seq	R_ENCBS054CPR
Blueprint	ENCFF155HAH	thymus	DNase1-seq	R_ENCBS198CXJ
Blueprint	ENCFF681JOT	thymus	DNase1-seq	R_ENCBS198CXJ
Blueprint	ENCFF707FJO	thymus	DNase1-seq	R_ENCBS198CXJ
Blueprint	ENCFF870IEV	thymus	DNase1-seq	R_ENCBS198CXJ
Blueprint	ENCFF328VXJ	thymus	DNase1-seq	R_ENCBS198CXJ
Blueprint	ENCFF625QAW	thymus	DNase1-seq	R_ENCBS198CXJ
Blueprint	ENCFF278ZNT	heart	DNase1-seq	R_ENCBS172XKB
Blueprint	ENCFF210JWW	heart	DNase1-seq	R_ENCBS172XKB
Blueprint	ENCFF943TNE	heart	DNase1-seq	R_ENCBS172XKB
Blueprint	ENCFF151KDO	heart	DNase1-seq	R_ENCBS172XKB
Blueprint	ENCFF416ZBD	heart	DNase1-seq	R_ENCBS172XKB
Blueprint	ENCFF106CPX	heart	DNase1-seq	R_ENCBS407ALA
Blueprint	ENCFF923BVC	heart	DNase1-seq	R_ENCBS407ALA
Blueprint	ENCFF007SPW	heart	DNase1-seq	R_ENCBS407ALA
Blueprint	ENCFF311OBU	heart	DNase1-seq	R_ENCBS407ALA
Blueprint	ENCFF452AYP	heart	DNase1-seq	R_ENCBS407ALA
Blueprint	ENCFF450AJZ	renal cortex interstitium	DNase1-seq	R_ENCBS620YJZ
Blueprint	ENCFF761BFW	renal cortex interstitium	DNase1-seq	R_ENCBS448HVV
Blueprint	ENCFF553VIY	renal cortex interstitium	DNase1-seq	R_ENCBS026RJE
Blueprint	ENCFF971PIK	renal cortex interstitium	DNase1-seq	R_ENCBS026RJE
Blueprint	ENCFF697SCS	renal cortex interstitium	DNase1-seq	R_ENCBS026RJE

Blueprint	ENCFF289UKR	renal cortex interstitium	DNase1-seq	R_ENCBS026RJE
Blueprint	ENCFF206TOI	adrenal gland	DNase1-seq	R_ENCBS232ONZ
Blueprint	ENCFF129QFX	adrenal gland	DNase1-seq	R_ENCBS232ONZ
Blueprint	ENCFF461SIX	adrenal gland	DNase1-seq	R_ENCBS660CJK
Blueprint	ENCFF294INJ	adrenal gland	DNase1-seq	R_ENCBS660CJK
Blueprint	ENCFF414WFK	adrenal gland	DNase1-seq	R_ENCBS200XAZ
Blueprint	ENCFF149EPY	adrenal gland	DNase1-seq	R_ENCBS200XAZ
Blueprint	ENCFF529NSR	adrenal gland	DNase1-seq	R_ENCBS200XAZ
Blueprint	ENCFF934GEX	adrenal gland	DNase1-seq	R_ENCBS200XAZ
Blueprint	ENCFF564QHA	adrenal gland	DNase1-seq	R_ENCBS200XAZ
Blueprint	ENCFF702FIW	adrenal gland	DNase1-seq	R_ENCBS200XAZ
Blueprint	ENCFF916IPH	adrenal gland	DNase1-seq	R_ENCBS200XAZ
Blueprint	ENCFF217BAD	adrenal gland	DNase1-seq	R_ENCBS119WRO
Blueprint	ENCFF783HCO	adrenal gland	DNase1-seq	R_ENCBS119WRO
Blueprint	ENCFF828MHF	adrenal gland	DNase1-seq	R_ENCBS119WRO
Blueprint	ENCFF111FYY	adrenal gland	DNase1-seq	R_ENCBS119WRO
Blueprint	ENCFF045ZNO	renal pelvis	DNase1-seq	R_ENCBS262QPN
Blueprint	ENCFF785AHD	renal pelvis	DNase1-seq	R_ENCBS262QPN
Blueprint	ENCFF568EUW	renal pelvis	DNase1-seq	R_ENCBS142XDW
Blueprint	ENCFF779WIH	renal pelvis	DNase1-seq	R_ENCBS142XDW
Blueprint	ENCFF287NFY	renal pelvis	DNase1-seq	R_ENCBS142XDW
Blueprint	ENCFF678HJR	renal pelvis	DNase1-seq	R_ENCBS785KTZ
Blueprint	ENCFF539CMH	renal pelvis	DNase1-seq	R_ENCBS785KTZ
Blueprint	ENCFF128PNW	renal pelvis	DNase1-seq	R_ENCBS785KTZ
Blueprint	ENCFF315MZE	renal pelvis	DNase1-seq	R_ENCBS785KTZ
Blueprint	ENCFF048UBW	left kidney	DNase1-seq	R_ENCBS610XAP
Blueprint	ENCFF467BCS	left kidney	DNase1-seq	R_ENCBS610XAP
Blueprint	ENCFF629QCK	left renal cortex interstitium	DNase1-seq	R_ENCBS376PWL
Blueprint	ENCFF776RFT	left renal cortex interstitium	DNase1-seq	R_ENCBS376PWL
Blueprint	ENCFF711KKQ	left renal cortex interstitium	DNase1-seq	R_ENCBS674SNK
Blueprint	ENCFF159XPF	left renal cortex interstitium	DNase1-seq	R_ENCBS674SNK
Blueprint	ENCFF072IHB	left renal cortex interstitium	DNase1-seq	R_ENCBS281CNH
Blueprint	ENCFF024ZWA	left renal cortex interstitium	DNase1-seq	R_ENCBS281CNH
Blueprint	ENCFF155MWH	left renal cortex interstitium	DNase1-seq	R_ENCBS636QOC
Blueprint	ENCFF874CGL	left renal cortex interstitium	DNase1-seq	R_ENCBS636QOC
Blueprint	ENCFF083MLN	left renal cortex interstitium	DNase1-seq	R_ENCBS636QOC
Blueprint	ENCFF785JRW	left renal cortex interstitium	DNase1-seq	R_ENCBS636QOC
Blueprint	ENCFF991TVK	left renal cortex interstitium	DNase1-seq	R_ENCBS636QOC
Blueprint	ENCFF417LLB	left renal cortex interstitium	DNase1-seq	R_ENCBS636QOC
Blueprint	ENCFF584SLG	left renal cortex interstitium	DNase1-seq	R_ENCBS636QOC
Blueprint	ENCFF954SPU	left renal cortex interstitium	DNase1-seq	R_ENCBS636QOC
Blueprint	ENCFF423HOT	left renal pelvis	DNase1-seq	R_ENCBS055ULH
Blueprint	ENCFF601LYG	left renal pelvis	DNase1-seq	R_ENCBS055ULH
Blueprint	ENCFF544DPT	left renal pelvis	DNase1-seq	R_ENCBS055ULH
Blueprint	ENCFF946SSJ	left renal pelvis	DNase1-seq	R_ENCBS257BTU
Blueprint	ENCFF657QAX	left renal pelvis	DNase1-seq	R_ENCBS257BTU
Blueprint	ENCFF639WUS	left renal pelvis	DNase1-seq	R_ENCBS257BTU
Blueprint	ENCFF431EYP	left renal pelvis	DNase1-seq	R_ENCBS226ZND
Blueprint	ENCFF065NFR	left renal pelvis	DNase1-seq	R_ENCBS226ZND
Blueprint	ENCFF767VEQ	left renal pelvis	DNase1-seq	R_ENCBS226ZND
Blueprint	ENCFF771XJT	left renal pelvis	DNase1-seq	R_ENCBS226ZND
Blueprint	ENCFF768GNX	left renal pelvis	DNase1-seq	R_ENCBS754ANY
Blueprint	ENCFF061DNQ	left renal pelvis	DNase1-seq	R_ENCBS754ANY

Blueprint	ENCFF806MUH	left renal pelvis	DNase1-seq	R_ENCBS754ANY
Blueprint	ENCFF052JNW	left renal pelvis	DNase1-seq	R_ENCBS754ANY
Blueprint	ENCFF909ULM	left renal pelvis	DNase1-seq	R_ENCBS754ANY
Blueprint	ENCFF770YNO	left renal pelvis	DNase1-seq	R_ENCBS754ANY
Blueprint	ENCFF445HLC	left renal pelvis	DNase1-seq	R_ENCBS754ANY
Blueprint	ENCFF027YDE	right renal pelvis	DNase1-seq	R_ENCBS145EYH
Blueprint	ENCFF423KHY	right renal pelvis	DNase1-seq	R_ENCBS145EYH
Blueprint	ENCFF245ELJ	right renal pelvis	DNase1-seq	R_ENCBS935VKR
Blueprint	ENCFF863PCQ	right renal pelvis	DNase1-seq	R_ENCBS935VKR
Blueprint	ENCFF009UUH	right renal pelvis	DNase1-seq	R_ENCBS935VKR
Blueprint	ENCFF530YSE	right renal pelvis	DNase1-seq	R_ENCBS855RFN
Blueprint	ENCFF892SRC	right renal pelvis	DNase1-seq	R_ENCBS855RFN
Blueprint	ENCFF889RBT	right renal pelvis	DNase1-seq	R_ENCBS855RFN
Blueprint	ENCFF974YUN	right renal pelvis	DNase1-seq	R_ENCBS827OFK
Blueprint	ENCFF795TJS	right renal pelvis	DNase1-seq	R_ENCBS827OFK
Blueprint	ENCFF138BJK	right renal pelvis	DNase1-seq	R_ENCBS827OFK
Blueprint	ENCFF733MIZ	right renal pelvis	DNase1-seq	R_ENCBS827OFK
Blueprint	ENCFF554OEC	right renal pelvis	DNase1-seq	R_ENCBS827OFK
Blueprint	ENCFF719GVR	right renal pelvis	DNase1-seq	R_ENCBS827OFK
Blueprint	ENCFF694DBL	right renal pelvis	DNase1-seq	R_ENCBS827OFK
Blueprint	ENCFF260GSJ	spinal cord	DNase1-seq	R_ENCBS373RUA
Blueprint	ENCFF369QCM	spinal cord	DNase1-seq	R_ENCBS373RUA
Blueprint	ENCFF222EIH	spinal cord	DNase1-seq	R_ENCBS300UPT
Blueprint	ENCFF854UHG	spinal cord	DNase1-seq	R_ENCBS300UPT
Blueprint	ENCFF073KRY	right renal cortex interstitium	DNase1-seq	R_ENCBS100DZU
Blueprint	ENCFF709HXO	right renal cortex interstitium	DNase1-seq	R_ENCBS100DZU
Blueprint	ENCFF102SFO	right renal cortex interstitium	DNase1-seq	R_ENCBS100DZU
Blueprint	ENCFF391HSJ	right renal cortex interstitium	DNase1-seq	R_ENCBS183TBX
Blueprint	ENCFF855JXV	right renal cortex interstitium	DNase1-seq	R_ENCBS183TBX
Blueprint	ENCFF934XHE	right renal cortex interstitium	DNase1-seq	R_ENCBS818WCN
Blueprint	ENCFF455IUH	right renal cortex interstitium	DNase1-seq	R_ENCBS818WCN
Blueprint	ENCFF863QQW	right renal cortex interstitium	DNase1-seq	R_ENCBS818WCN
Blueprint	ENCFF948AVM	right renal cortex interstitium	DNase1-seq	R_ENCBS818WCN
Blueprint	ENCFF882KUN	right renal cortex interstitium	DNase1-seq	R_ENCBS818WCN
Blueprint	ENCFF705NSS	right renal cortex interstitium	DNase1-seq	R_ENCBS818WCN
Blueprint	ENCFF838LOC	right renal cortex interstitium	DNase1-seq	R_ENCBS818WCN
Blueprint	ENCFF422YYG	spleen	DNase1-seq	R_ENCBS599HUG
Blueprint	ENCFF525TWK	psoas muscle	DNase1-seq	R_ENCBS008QPC
Blueprint	ENCFF457YXQ	psoas muscle	DNase1-seq	R_ENCBS008QPC
Blueprint	ENCFF940GXC	psoas muscle	DNase1-seq	R_ENCBS008QPC
Blueprint	ENCFF136KBO	psoas muscle	DNase1-seq	R_ENCBS008QPC
Blueprint	ENCFF065TLM	muscle of trunk	DNase1-seq	R_ENCBS992XAC
Blueprint	ENCFF979HOG	ovary	DNase1-seq	R_ENCBS645JEU
Blueprint	ENCFF659QQK	ovary	DNase1-seq	R_ENCBS645JEU
Blueprint	ENCFF827YHI	ovary	DNase1-seq	R_ENCBS341OKA
Blueprint	ENCFF331JVF	ovary	DNase1-seq	R_ENCBS341OKA
Blueprint	ENCFF780LKZ	pancreas	DNase1-seq	R_ENCBS507RPJ
Blueprint	ENCFF890UCG	pancreas	DNase1-seq	R_ENCBS507RPJ
Blueprint	ENCFF985FNS	pancreas	DNase1-seq	R_ENCBS914JTX
Blueprint	ENCFF457ONU	pancreas	DNase1-seq	R_ENCBS914JTX
Blueprint	ENCFF109QYQ	testis	DNase1-seq	R_ENCBS796DWQ
Blueprint	ENCFF025KGA	testis	DNase1-seq	R_ENCBS796DWQ
Blueprint	ENCFF249RTG	forelimb muscle	DNase1-seq	R_ENCBS988KQJ

Blueprint	ENCFF949DLJ	hindlimb muscle	DNase1-seq	R_ENCBS105LQM
Blueprint	ENCFF135DMC	hindlimb muscle	DNase1-seq	R_ENCBS105LQM
Blueprint	ENCFF256YZN	H1-hESC	DNase1-seq	R_ENCBS559QNR_ENCBS568FYY_ENCBS945MCY
Blueprint	ENCFF217JMU	H1-hESC	DNase1-seq	R_ENCBS559QNR_ENCBS568FYY_ENCBS945MCY
Blueprint	ENCFF390EVX	H1-hESC	DNase1-seq	R_ENCBS559QNR_ENCBS568FYY_ENCBS945MCY
Blueprint	C0011IH1.ERX197160.H3K27ac.bwa.GRCh38.20150528.bw	CD14-positive, CD16-negative classical monocyte	H3K27ac	C0011IH1
Blueprint	S00C0JH1.ERX547233.H3K27ac.bwa.GRCh38.20150528.bw	macrophage_-_T_6days_B-glucan	H3K27ac	S00C0JH1
Blueprint	S00XUNH1.ERX941029.H3K27ac.bwa.GRCh38.20150529.bw	Acute Myeloid Leukemia	H3K27ac	S00XUNH1
Blueprint	C0010KH1.ERX197174.H3K27ac.bwa.GRCh38.20150528.bw	CD14-positive, CD16-negative classical monocyte	H3K27ac	C0010KH1

Supplementary Table S1: Data IDs of the raw data files processed in this study.

Gen	Considered REM	Deleted Region	gRNA binding sites
KLF2	chr19:16330707-16330756	chr19:16330698-16330768	agaccagcgagcatcagcct agctgatttgtcaggtgcca
NOS3	chr7:150988705-150989104	chr7:150988600-150989134	gtgggatcagacaccacgaa atgcacacaccccaggccca
AC020916	chr19:13846946-13847045	chr19:13846916-13847171	tgggtggtgagtcattcctg ggctcagtccataacacagc

Supplementary Table S2: position of the considered REMs, the position of the deleted genomic region per gene and the gRNA sequences

98°C	98°C	66-69°C	72°C	72°C
30sec	10sec	30sec	20sec	2min
	30 cyles			

Supplementary Table S3: PCR schedule

	Primer 1	Primer 2
KLF2	AGAGGGTCTCCCTCGATGAC	CTAGTGTAGACCCGGTGGGA
NOS3	GCCCGGGACTTCATCAACCA	TCCTGTGCAGACCTGCAGTC
AC020916	CCTAGCAGCCAGTTACCCAA	TCCCGGCAAGTTAGAAAGCT

Supplementary Table S4: PCR primers

Gene	Ratio to Beta-Actin	Experiment	ID
KLF2	0.250981	KO	B1
KLF2	0.161633	KO	B2
KLF2	0.257367	KO	B3
KLF2	0.63829	CTRL	CTRL1
KLF2	0.877955	CTRL	CTRL2
KLF2	0.716675	CTRL	CTRL3
AC020916	1.150943	KO	C1
AC020916	1.409244	KO	C2
AC020916	1.850856	KO	C3
AC020916	0.362736	CTRL	CTRL4
AC020916	1.359852	CTRL	CTRL5
AC020916	0.2635523	CTRL	CTRL6
NOS3	0.390649	KO	D1
NOS3	0.425132	KO	D2
NOS3	0.364058	KO	D3
NOS3	0.326564	CTRL	CTRL7
NOS3	0.594601	CTRL	CTRL8
NOS3	0.622969	CTRL	CTRL9

Supplementary Table S5: Quantification results

	Blueprint	Roadmap
**STITCHIT**	162,453,061	165,805,230
**Unified Peaks**	165,590,118	273,259,579
**GeneHancer**	137,722,391	155,548,667

Supplementary Table S6: Total genomic space covered by the suggested regulatory regions shown for each dataset.

Supplementary Table S7 is an external excel sheet that is available as a separate file.

**STITCHIT**					
**chr**	**start**	**end**	**Reem ID**	**coefficient**	**OLS p-value**
chr5	138442350	138442489	S4	0.12347378	0.13772486
chr5	138449000	138449499	S7	0.06534721	0.52094523
chr5	138450250	138450399	S10	-0.03976734	0.65534283
chr5	138454490	138456489	S9	0.061487	0.55857386
chr5	138457200	138457409	S6	0.07735484	0.40351668
chr5	138465700	138465949	S2	0.21150264	0.05986547
chr5	138469650	138470349	S10	0.45303257	0.00021482
chr5	138475650	138476489	S5	0.11132624	0.22930495
chr5	138486600	138486749	S3	-0.15254059	0.15120576
chr5	138486950	138487099	S8	-0.06192892	0.56018674
**Unified Peaks**					
**chr**	**start**	**end**	**Reem ID**	**coefficient**	**OLS p-value**
chr5	138443499	138444336	U11	-0.02633325	0.84467668
chr5	138446871	138447822	U10	0.04075988	0.76654673
chr5	138454281	138454728	U4	0.19843172	0.11272934
chr5	138454842	138455474	U7	0.07477763	0.58821766
chr5	138462649	138471619	U1	0.4100908	0.00147926
chr5	138471717	138473719	U9	-0.04813992	0.77152777
chr5	138473888	138474432	U5	0.11320651	0.45458859
chr5	138475851	138476436	U3	0.21427014	0.12540462
chr5	138476499	138477251	U6	0.10563739	0.4268543
chr5	138483081	138483386	U8	0.00735699	0.95430046
chr5	138486580	138487561	U2	-0.25307334	0.09496258

Supplementary Table S8: Candidate regulatory sites for EGR1.

	Blueprint	Roadmap
**Activators**	SNAI2, RHOXF1, RUNX2, NKX2.5.VAR.2., NR2F6.VAR.2., MNT, RFX1, REL, NR4A2, MAX, RARB, NFIC, RXRA::VDR, PKNOX1, NKX2.8, PKNOX2, PPARG, DDIT3::CEBPA, RUNX3, NR1H3::RXRA, YY2, RARA, HNF4A, NFE2, TP53, TGIF2, YY1, ZNF143, TGIF1, NR2F6, HES2, MSC, RFX4, PAX2, RXRA, NKX2.3, OTX1, SMAD3, RXRG, TBX21, RXRB, GFI1B	RUNX2, ETV4, ETS1, ELF1, PPARG, FLI1, RUNX3, CREB3, EHF, FEV, RELA, REL, ETV3, RXRA, AR, ERG, XBP1
**Repressors**	ELK3, ETV4, ERF, ETS1, ELF5, ELF1, FEV, FLI1	FOSL2, NFE2, FOSL1, NFE2L2, JUND, RHOXF1, TP53, JUNB

Supplementary Table S9: TFs enriched in REMs acting exclusively as activators or repressors.

Adipose_Subcutaneous.tsv.gz	3761037 KB	4/28/2020	8:00:00 AM
Adipose_Visceral_Omentum.tsv.gz	3722944 KB	4/28/2020	8:00:00 AM
Adrenal_Gland.tsv.gz	3460912 KB	4/28/2020	8:00:00 AM
Artery_Aorta.tsv.gz	3555967 KB	4/28/2020	8:00:00 AM
Artery_Coronary.tsv.gz	3558142 KB	4/28/2020	8:00:00 AM
Artery_Tibial.tsv.gz	3534092 KB	4/28/2020	8:00:00 AM
Brain_Amygdala.tsv.gz	3324281 KB	4/28/2020	8:00:00 AM
Brain_Anterior_cingulate_cortex_BA24.tsv.gz	3397718 KB	4/28/2020	8:00:00 AM
Brain_Caudate_basal_ganglia.tsv.gz	3552232 KB	4/28/2020	8:00:00 AM
Brain_Cerebellar_Hemisphere.tsv.gz	3571733 KB	4/28/2020	8:00:00 AM
Brain_Cerebellum.tsv.gz	3673725 KB	4/28/2020	8:00:00 AM
Brain_Cortex.tsv.gz	3572746 KB	4/28/2020	8:00:00 AM
Brain_Frontal_Cortex_BA9.tsv.gz	3499897 KB	4/28/2020	8:00:00 AM
Brain_Hippocampus.tsv.gz	3450621 KB	4/28/2020	8:00:00 AM
Brain_Hypothalamus.tsv.gz	3533843 KB	4/28/2020	8:00:00 AM
Brain_Nucleus_accumbens_basal_ganglia.tsv.gz	3571953 KB	4/28/2020	8:00:00 AM
Brain_Putamen_basal_ganglia.tsv.gz	3364430 KB	4/28/2020	8:00:00 AM
Brain_Spinal_cord_cervical_c-1.tsv.gz	3402918 KB	4/28/2020	8:00:00 AM
Brain_Substantia_nigra.tsv.gz	3333448 KB	4/28/2020	8:00:00 AM
Breast_Mammary_Tissue.tsv.gz	3878078 KB	4/28/2020	8:00:00 AM
Cells_Cultured_fibroblasts.tsv.gz	3293987 KB	4/28/2020	8:00:00 AM
Cells_EBV-transformed_lymphocytes.tsv.gz	3253180 KB	4/28/2020	8:00:00 AM
Colon_Sigmoid.tsv.gz	3616741 KB	4/28/2020	8:00:00 AM
Colon_Transverse.tsv.gz	3815598 KB	4/28/2020	8:00:00 AM
Esophagus_Gastroesophageal_Junction.tsv.gz	3566549 KB	4/28/2020	8:00:00 AM
Esophagus_Mucosa.tsv.gz	3619435 KB	4/28/2020	8:00:00 AM
Esophagus_Muscularis.tsv.gz	3586728 KB	4/28/2020	8:00:00 AM
Heart_Atrial_Appendage.tsv.gz	3436472 KB	4/28/2020	8:00:00 AM
Heart_Left_Ventricle.tsv.gz	3157537 KB	4/28/2020	8:00:00 AM
Kidney_Cortex.tsv.gz	3316194 KB	4/28/2020	8:00:00 AM
Liver.tsv.gz	3193315 KB	8/26/2020	8:00:00 AM
Lung.tsv.gz	3975800 KB	4/28/2020	8:00:00 AM
Minor_Salivary_Gland.tsv.gz	3660276 KB	4/28/2020	8:00:00 AM
Muscle_Skeletal.tsv.gz	3202926 KB	4/28/2020	8:00:00 AM
Nerve_Tibial.tsv.gz	3935598 KB	4/28/2020	8:00:00 AM
Ovary.tsv.gz	3634439 KB	4/28/2020	8:00:00 AM
Pancreas.tsv.gz	3329924 KB	4/28/2020	8:00:00 AM
Pituitary.tsv.gz	3885080 KB	4/28/2020	8:00:00 AM
Prostate.tsv.gz	3891108 KB	4/28/2020	8:00:00 AM
Skin_Not_Sun_Exposed_Suprapubic.tsv.gz	3856136 KB	4/28/2020	8:00:00 AM
Skin_Sun_Exposed_Lower_leg.tsv.gz	3859784 KB	4/28/2020	8:00:00 AM
Small_Intestine_Terminal_Ileum.tsv.gz	3811791 KB	4/28/2020	8:00:00 AM
Spleen.tsv.gz	3761414 KB	4/28/2020	8:00:00 AM
Stomach.tsv.gz	3611468 KB	4/28/2020	8:00:00 AM
Testis.tsv.gz	5309149 KB	4/28/2020	8:00:00 AM
Thyroid.tsv.gz	3980170 KB	4/28/2020	8:00:00 AM
Uterus.tsv.gz	3563847 KB	4/28/2020	8:00:00 AM
Vagina.tsv.gz	3652081 KB	4/28/2020	8:00:00 AM
Whole_Blood.tsv.gz	3115773 KB	4/28/2020	8:00:00 AM
BLUEPRINT_ge_T-cell.all.tsv.gz	2300691 KB	12/4/2020	6:10:00 AM
BLUEPRINT_ge_monocyte.all.tsv.gz	2169918 KB	12/4/2020	6:10:00 AM
BLUEPRINT_ge_neutrophil.all.tsv.gz	1745342 KB	12/4/2020	6:11:00 AM

Supplementary Table S10: eQTL files obtained from EMBL's eQTL catalogue.

References

[1] Martens JHA, Stunnenberg HG. BLUEPRINT: mapping human blood cell epigenomes, *Haematologica*. 2013;98(10):1487-1489. doi:10.3324/haematol.2013.094243.

[2] Roadmap Epigenomics Consortium, Kundaje A, Meuleman W, et al. Integrative analysis of 111 reference human epigenomes, Nature. 2015;518(7539):317-330. doi:10.1038/nature14248.

[3] Patro R, Duggal G, Love MI, Irizarry RA, Kingsford C. Salmon: fast and bias-aware quantification of transcript expression using dual-phase inference, Nature methods. 2017;14(4):417-419. doi:10.1038/nmeth.4197.

[4] Harrow et al., GENCODE: the reference human genome annotation for the ENCODE Project, Genome research. 2012;22;0;1760-74; doi:10.1101/gr.135350.111.

[5] Langmead B, Salzberg SL. Fast gapped-read alignment with Bowtie 2, *Nature methods*. 2012;9(4):357-359. doi:10.1038/nmeth.1923.

[6] Li H, Handsaker B, Wysoker A, et al. The Sequence Alignment/Map format and SAMtools, Bioinformatics. 2009;25(16):2078-2079. doi:10.1093/bioinformatics/btp352.

[7] Ibrahim MM, Lacadie SA, Ohler U. JAMM: a peak finder for joint analysis of NGS replicates, Bioinformatics. 2015;1;31(1):48-55. doi:10.1093/bioinformatics/btu568.

[8] Fidel, R., Ryan, DP., Gruening, B., et al., deepTools2: A next Generation Web Server for Deep-Sequencing Data Analysis, Nucleic Acids Res, 2016, 44:W160-W165.

[9] Garret, E.S. and Parmigiani, G., POE: Statistical Methods for Qualitative Analysis of Gene Expression, In: Parmigiani G., Garrett E.S., Irizarry R.A., Zeger S.L. (eds) The Analysis of Gene Expression Data. Springer 2003, ISBN 978-0-387-95577-3.

[10] Terzi E. Problems and algorithms for sequence segmentations, Helsinki, 2006, ISBN: 952-10-3520-X.

[11] Grünwald P. D., The minimum description length principle, MIT press 2007, ISBN: 9780262072816.

[12] Nguyen H.-V., Vreeken J. Flexibly Mining Better Subgroups. SDM, 585—593, SIAM, 2016

[13] Schmidt et al. Combining transcription factor binding affinities with open chromatin data for accurate gene expression prediction, NAR.2017;45;1:54–66.doi: 10.1093/nar/gkw1061

[14] Fishilevich, S., et al., GeneHancer: genome-wide integration of enhancers and target genes in GeneCards, Database (Oxford). 2017 Jan 1;2017. doi: 10.1093/database/bax028.

[15] Quinlan, AR. and Hall, IM., BEDTools: a flexible suite of utilities for comparing genomic features, Bioinformatics. 2010. 26(6):841-842.

[16] Andersson, R. et al, An atlas of active enhancers across human cell types and tissues, Nature, 2014. Doi: 10.1038/nature12787
